# Posters

**DOI:** 10.1155/1993/79836

**Published:** 1993


PO001

### THE PROTECTIVE ROLE OF MANNITOL IN EXPERIMENTAL ISCHEMIA AND REPERFUSION-INDUCED LIVER INJURY

S Sokmen,A Mentes,I Mutaf

O Bayındır,G Yuce,Y Yuzer

Surgical Unit,Aegean University Medical School,
Izmir,Turkey

There is now a large data base incriminating the oxygen free radical system as an important mechanism of ischemic liver injury.The purpose of this study was to examine the effectiveness of mannitol over well-known free radical scavengers in preventing liver damage from controlled hepatic ischemia and reperfusion.Regional hepatic ischemia was induced by occluding the vessels to the left and median lobes of the liver for one hour.The reperfusion was established by removing the occlusion at the end of the hour and the animals were sacrificed after 24hours of reperfusion.There were two control groups;sham-op. controls(n:7)and non-treated ischemic controls(n:8),and other four study groups;mannitol pretreated,superoxide dismutase('SOD')treated, verapamil treated and SOD+Verapamil treated(n:9,10,10,7).The liver ATP level was significantly higher(p 0.01) than ischemic controls, however it was indifferent compared to sham operated rats or rats protected by control scavengers.The plasma lipid peroxidase level was significantly lower in the mannitol pretreated group compared to ischemic controls(p 0.01),but similar to verapamil and SOD+Verapamil treated groups.We conclude that mannitol serves comparibly to other known oxygen radical scavengers in preventing the liver cell from toxic damage after ischemia and reperfusion injury of the liver.

PO002 **ULTRASTRUCTURAL DYNAMIC CHANGES IN REGENERATING HEPATIC CELLS FOLLOWING PARTIAL HEPATECTOMY IN RATS**

Han Shu-Yuan, M.D., Huang Zhi-Qiang MD
Yu Gue

4-5, 401, No. 3 Xicui Road, Fuxing Men
Wai, Beijing 100039, CHINA

The paper presented herein is to study characteristics of ultrastructural changes in the regenerating hepatic cells following hepatectomy. Healthy Wistar rats of either sex weighing 200-270g were used. Rats underwent partial hepatectomy were performed under anesthesia with sodium pentobarbital and an amount of 40% of the liver tissue were removed. At time intervals of 10,15,20,25, 30 and 35 days post resection liver tissues were removed and prepared for electromicroscopy. The treatment group animals received ATP and 654-2 at the 3rd and 7th post-operative day. In another group animals were rendered cirrhotic by hypodermic injection of CCl4 and feed with 5% alcoholic solution. Serial studies of ultrastructural changes of liver cells after partial hepatectomy revealed evidence of enhaced cellular metabolism namely : - increase and aggregation of roughened endoplasmic reticulum; swelling of mitochondria with accumulation of glycogen particles; and nuclear enlargement. But in the cirrhotic group ultrastructural changes were mainly hypertrophy of collengenous fibrials with little functionl liver cells. It also revealed that 654-2 and ATP post-operatively can promote liver cell regeneration. It is therefore suggested that fractional resection of liver and treatment with 654-2 and ATP may be beneficial in treating begign hepatic lesions, but cautions must be paid in handling cirrotic patients.

PO003

### RESECTION OF HEPATO-CELLULAR CARCINOMAS WITH CIRRHOSIS - A MULTICENTRIC STUDY ABOUT 153 PATIENTS

AURC - ARC - P Vicq - D Houssin - J Pitre

Val de Grace Hospital, 74 Boulevard de Port Royal - 75230, Paris, Cedex O5, France

Hepato cellular carcinoma with cirrhosis is mainly treated by surgical resection, when possible. In order to identify the prognostic factors of the resection of hepato cellular carcinomas in cirrhosis, the results of 153 resections, performed between January 1984 and December 1988 in 18 French centres, were analysed, under the control of the French Associations for Surgical Research.

Among post-operative complications (61 per cent) the most frequent were ascites (35,3 per cent) and liver failure (19 per cent). Operative mortality was 20 per cent. One, 3 and 5 year survival rates were 52,7 per cent, 30, 1 per cent, and 17,9 per cent, respectively. The survival rate was significantly higher in patients with a curative resection, of Pugh's class A or with a tumour less than 3 centimetres in diameter. After curative resection, actuarial survival rates without recurrence were 65 per cent, 24,4 per cent and 16,7 per cent after 1, 3 and 4 years. In this case, the survival rate was significantly related to the number of resected nodules and the size of the tumour, but not to the presence of a capsule surrounding the tumour.

PO004

### ENTERAL CHOLESTYRAMINE PREVENTS FALL IN RENAL BLOOD FLOW FOLLOWING SURGERY IN OBSTRUCTIVE JAUNDICE.

A Houdijk, P van Leeuwen, M Boermeester and R Wesdorp.

Department of Surgery, Free University Hospital,
Amsterdam, The Netherlands.

Acute renal failure (ARF) is common after surgery in obstructive jaundice and associated with systemic endotoxemia and reduced renal blood flow (RBF). We hypothesized that the reduction in RBF is directly related to gut derived endotoxin.

To test this hypothesis, male Wistar rats (n = 20 per group, weight 250-300 g.) received the endotoxin binder cholestyramine (CHOL, 150 mg/day) or saline (SAL) twice daily in the same volume. This treatment started on Day 1 and was continued until the end of the experiment. On Day 7 groups were randomized to receive a SHAM operation (SH) or bile duct ligation (BDL). This resulted in 4 groups (n = 10 each) i.e. SH-SAL, SH-CHOL, BDL-SAL and BDL-CHOL. Subsequently rats were subjected to a surgical trauma by performing a xyphoidectomy on Day 20 and blood flow measurements were done the following day using the microsphere technique.
A portion of the data of the RBF through both kidneys is shown and expressed in mlmin$^{-1}$ (mean ± SEM).

| Groups | SHAM (n = 20) | BDL-SAL (n = 10) | BDL-CHOL (n = 10) |
|---|---|---|---|
| RBF | 14.78 ± 0.63* | 11.24 ± 0.95 | 14.58 ± 0.73# |

*,# $p<0.01$ vs BDL-SAL. Students T-test using Bonferroni correction.

Surgery in saline treated BDL rats resulted in a significant decrease in RBF if compared to both SHAM groups (cholestyramine had no effect in the SHAM group). This decrease was not seen when BDL animals were treated with cholestyramine before surgery. Similar results were seen when flow per gram tissue was calculated in the different groups.

In conclusion: these data demonstrate that the reduction in RBF following surgery in rats with obstructive jaundice is prevented by enteral treatment with cholestyramine. Endotoxemia is probably the most important etiologic factor in the development of postoperative ARF in obstructive jaundice.

PO005 NUCLEOLAR ORGANIZER REGIONS IN THE DIFFERENT HEPATIC LOBES AFTER END-TO-SIDE PORTACAVAL SHUNT IN THE RAT.

MA Aller, JL Arias, L Lorente, G Vallejo, LJ Santín, JI Trobo, J Arias

Psychobiology Lab. University of Oviedo and Surgery I Dept. Complutense University Madrid. Spain

The activity of protein synthesis in the hepatic lobes in control and end-to-side portacaval shunt rats is studied in this experimental work. To do so, we haven chosen one of the components of the hepatocytic nucleolus, the nucleolar organizer regions, which are argyrophilic (Ag-NOR). The results show an increase in the hepatocytic nuclei in the caudate and middle lobes in portacaval shunt rats in relation to control rats; the middle lobe increase is statistically significant ($p < 0.05$). The Ag-NOR area is greater in the right lateral and caudate lobe in the control rats and this parameter increases in the middle and caudate lobes in portacaval shunt rats than in the control rats. The Ag-NOR area per nucleus is superior in the right lateral and caudate lobes in both the control and portacaval shunt rats. This parameter increases in the left lateral, middle and caudate lobes in portacaval shunt rats in relation to the control rats. The number of Ag-NORs per nucleus increases in all the hepatic lobes after the portacaval shunt and this increase is statistically significant ($p < 0.05$) in the middle lobe. In the parameters studied, the differences between the hepatic lobes of the control rats as well as their variations after a portacaval shunt make it possible to hypothesize the existence of a hepatic lobular functional heterogeneity in relation to the protein synthesis, which changes after the deprivation of portal flow produced by an end-to-side portacaval shunt.

PO006

### INTRAMUCOSAL pH AND OXYGEN EXTRACTION IN THE GASTRO-INTESTINAL TRACT FOLLOWING MAJOR LIVER RESECTION IN THE RAT

R Andersson, X D Wang, Q Wang, S Bengmark

Dept of Surgery, Lund University, Lund, Sweden

Abdominal sepsis and enteric bacterial translocation has been demonstrated following major liver resection. The present study aimed at exploring potential changes in homeostasis that might influence on enteric bacterial ecology.

Intestinal oxygen delivery, blood gas analysis of arterial, caval and portal vein blood, as well as direct intraluminal pH of the gastrointestinal tract (GIT), and indirect measurements of intramucosal pH in the GIT were evaluated after 70 % and 90 % hepatectomy in the rat.

A temporary increase in arterial pH occurred 2 h after 70 % hepatectomy. Intramucosal pH of the GIT decreased from 2 h and on following 90 % hepatectomy, reaching statistical significance at 4 h. Intraluminal pH of the GIT increased 2 and 4 h after 70 % and 90 % hepatectomy, as compared with animals with sham operation. Systemic oxygen extraction increased immediately following hepatectomy. GIT oxygen extraction gradually increased in 70 % hepatectomized animals, while a decrease was seen following 90 % hepatectomy.

We conclude that major liver resection induces alterations in systemic and GIT homeostasis, which may contribute to explain disturbances noted in enteric bacterial ecology, the increase in bacterial adherence onto the intestinal surface and the increased translocation of enteric bacteria from gut following major liver resection in the rat.

PO007

### BACTERIAL TRANSLOCATION, INTESTINAL ULTRASTRUCTURE AND PERMEABILITY IN THE EARLY STAGE FOLLOWING MAJOR LIVER RESECTION IN THE RAT

XD Wang, H Pärsson, R Andersson, V Soltesz, K Johansson, S Bengmark
Depts of Surgery, Medical Microbiology and Zoology, Lund University, Lund, Sweden

Enteric bacterial translocation has been demonstrated following major liver resection, the extent being proportional to the amount of liver removed. The process and routes of bacterial translocation from the gut after major liver resection, however, remain unclear.

In the present study, enteric bacterial translocation, enterocyte ultrastructure in the ileum and colon, the process and routes of invading bacteria normally preserved in the gut and the permeability of the cellular membrane system (CMS) and blood tissue barrier (BTB) were evaluated in rats receving sham operation, 70 % and 90 % hepatectomy.

The incidence of bacterial translocation to MLN was 80-100 % in rats with 70 % (6 h) and 90 % (2-4 h) hepatectomy, and 80-100 % to the systemic circulation 2-4 h following 90 % hepatectomy, while only 20 % to the portal vein. An increase in bacterial adherence onto the intestinal surface, damage of the permeability of the CMS and BTB, and pathological alterations in the ileum and colon developed, correlating with the extent of liver removed and the time that had passed following subtotal hepatectomy. Most translocating bacteria appeared in morphologically intact enterocytes, with increased membrane permeability, in the presence of antigen-presenting cells, and in the submucosal lymphatics, but also some bacteria were seen within damaged enterocytes from the 90 % hepatectomy-4 h group.

Our results indicate that an altered permeability of the CMS is one of the earliest changes in challenged enterocytes, and enteric bacteria translocate through both morphologically normal and abnormal enterocytes, mainly into the lymphatics, either being "carried" by antigen-presenting cells or actively invading by themselves.

PO008

### THE RELATIONSHIP BETWEEN ENTERIC BACTERIAL OVERGROWTH AND GASTROINTESTINAL MOTALITY FOLLOWING SUBTOTAL LIVER RESECTION OR PORTAL VEIN OBSTRUCTION IN THE RAT

X D Wang, W Guo, Q Wang, R Andersson, E Ekblad
V Soltesz, S Bengmark

Depts of Surgery, Histology and Clinical Microbiology,
Lund University, Lund, Sweden

Intestinal transit time is an important indicator of gastrointestinal tract function. The relationship between enteric bacterial overgrowth and intestinal transit time following subtotal liver resection has not been elucidated.

In the present study, intestinal morphology, immunocytochemistry of the enteric nervous system, enteric bacterial growth in the small intestine and colon as well as intestinal transit time were determined in rats subjected to sham operation, 90 % hepatectomy or portal vein obstruction.

Histo-pathological alterations and E.coli overgrowth in the intestine were observed 2 h after 90 % hepatectomy, while a delayed intestinal transit was noted already 1 h following 90 % hepatectomy. Intestinal transit time was significantly delayed in rats subjected to 90 % hepatectomy as compared with both animals with sham operation and portal vein obstruction. No difference in the intestinal transit time was noted between rats with portal vein obstruction and sham operation.

It is concluded that the delayed intestinal transit following 90 % hepatectomy may contribute to enteric bacterial overgrowth and concomitant bacterial translocation from the gut.

PO009

### ENTERAL ADMINISTRATION OF EHEC PREVENTS BACTERIAL TRANSLOCATION FROM THE GUT FOLLOWING SUBTOTAL LIVER RESECTION IN THE RAT

X D Wang, W Guo, Q Wang, R Andersson, V Soltesz, S Bengmark

Depts of Surgery and Medical Microbiology, Lund University, Lund, Sweden

We have previously demonstrated that bacterial translocation from the gut, resulting in intraabdoinal sepsis or bacteremia, occurs following major liver resection in both patients and animals. The aim of the present study was to evaluate the influence of a water soluble ethylhydroxyethyl cellulose (EHEC).

EHEC was administered 1 and 12 h prior to 90 % hepatectomy in the rat. 90 % hepatectomy resulted in 80-100 % enteric bacterial translocation to MLNs or blood 2 and 4 h after operation while translocation did not occur in rats undergoing sham operation or in animals with 90 % hepatectomy and EHEC pre-treatment ($p < 0.01$). Bacterial overgrowth, increased bacterial adherence on the intestinal surface, as well as diminished intestinal and mucosal mass were observed in animals with subtotal liver resection alone, but not in those with enteral administration of EHEC. A delay in intestinal 2 h transit time was present in both groups receiving subtotal liver resection, with or without EHEC. EHEC inhibited bacterial growth and DNA synthesis, and altered bacterial surface properties following 1 h interaction with bacteria.

In conclusion, EHEC seems to alter enterobacterial capacities of metabolism, proliferation and invasion by direct effects on bacterial surface characteristics, and possesses a trophic action on the intestine rather than enhancing intestinal motility following subtotal experimental liver resection.

PO010

### EFFECT OF CRYOPRESERVATIVE DURATION ON HEPATOCYTE VIABILITY

C Lin, WX Zhang, JN Lü, JH Wang, KY Hou, XS Zhou

Department of Surgery, Third Hospital
Beijing Medical University
Beijing, China

The experiment aims at studying the effect of cryopreservative duration on hepatocyte viability(VIA). Hepatocytes isolated from each of 10 Wistar rats were divided into 5 groups: cryopreserving for 1 month(CP1), 2 months (CP2), 3 months(CP3), 6 months(CP6), and fresh preparation (FP). In CP1, CP2, CP3, and CP6, the hepatocytes were frozen decrementally in cryopreservation solution, ultimately stored at -196°C, and rapidly thawed at 40°C when they became due. The VIA of hepatocytes was identified by trypan blue exclusion test. Then the percentage of viable cells was calculated. Glucose-6-phosphatase (G6Pase) and glycogen (GLY) were detected by histochemistry. Glutamic oxaloacetic transaminase(GOT), alkaline phosphatase (AKP), lactic dehydrogenase (LDH) and total protein (TPr) in homogenate of viable cell suspension ($2x10^6$ cells/ml) were measured. The difference of cell VIA between FP (92.7±3.7%) and any of the CP groups (CP1 71.2±11.2%, CP2 75.7±13.9%, CP3 69.7±13.6%, CP6 73.5± 13.5%) was significant ($p<0.05$), but the differences among CP groups was not significant($p>0.05$). No significant differences of G6Pase, GLY, GOT, AKP, LDH, and TPr contents revealed among all groups. Electron microscopy revealed the membranes and organellae of viable cryopreserved cells were as perfect as fresh cells.

CONCLUSION: After 1 month cryopreservation, the VIA of hepatocytes decreased significantly, but no longer decreased in subsequent freeze. The structure and G6Pase, GLY, GOT, AKP, LDH, and TPr contents of viable cells in 5 groups are the same. The hepatocytes cryopreservation for long duration is practical.

PO011

### VASOPRESSIN AND ORGANIC NITRATES: EFFECTS ON PORTAL HAEMODYNAMICS

**J. Yates, D.M. Nott, H. Kynaston,. L. Jinlai, D. Billington, R. Shields and S.A. Jenkins**

**Department of Surgery, Royal Liverpool University Hospital, Liverpool, U.K.**

Nitrates have been reported to improve the efficacy of vasopressin in controlling variceal bleeding. Since there is a paucity of data on the haemodynamic effects of such combination therapy during hypovolaemia, we have undertaken such a study in portal hypertensive rats.

Portal hypertensive rats (partial portal vein ligation) were bled at a constant rate until the systolic blood pressure was 50 mm Hg. After a period of stabilisation groups of rats received continuous infusions of vasopressin (0.8 $\mu$U/g/h), isorbide mononitrate (10 $\mu$g/kg/min) a combination of vasopressin and isorbide or saline. Portal pressure and arterial blood pressure were measured continuously and collateral blood flow (consecutive intrasplenic injection of $^{99}$Tc-methylene diphosphonate and $^{99}$Tc-albumin microspheres) every 5 min throughout the study.

Haemorrhage decreased portal pressure but increased collateral blood flow in all four groups of rats ($p < 0.001$ ANOVA). Administration of vasopressin, saline or a combination of isorbide and vasopressin had no effect on portal pressure or collateral blood flow during hypovolaemia. In contrast, isorbide alone increased portal pressure (6.5 $\pm$ 0.4 to 8.3 $\pm$ 0.4 mm Hg; $p < 0.02$) but decreased collateral blood flow (62.0 $\pm$ 14.5 to 34.5 $\pm$ 10.9%, $p < 0.02$) in hypovolaemic rats.

Since the efficacy of vasoactive drugs in controlling variceal bleeding depends upon their ability to reduce collateral blood flow, isorbide alone would appear to be more effective than either vasopressin alone or combination therapy during hypovolaemia.

PO012

### HYPOVOLAEMIA AND REPERFUSION IN PORTAL HYPERTENSION: EFFECTS OF SOMATOSTATIN, OCTREOTIDE AND VASOPRESSIN.

**J. Yates, D.M. Nott, H. Kynaston, N. Davies, L. JinLai, D. Billington, R. Shields & S.A. Jenkins**

**Department of Surgery, Royal Liverpool University Hospital, Liverpool, U.K.**

The majority of patients receiving vasoactive drugs to control variceal bleeding are hypovolaemic and in the process of being resuscitated during therapy. However, since there is a paucity of data on the effects of vasoactives on portal haemodynamics during hypovolaemia and reperfusion we have undertaken such a study in portal hypertensive rats.

Hypovolaemic, portal hypertensive rats (partial portal vein ligation) received infusions of somatostatin (0.4 μg/kg/h) octreotide (0.4 μg/kg/h) vasopressin (0.08 μU/g/h) or saline. Reperfusion was commenced 15 min after the start of drug administration. Portal pressure and arterial blood pressure were measured continuously and collateral blood flow (consecutive intrasplenic administration of $^{99}$Tc-methyl diphosphonate and $^{99}$Tc-albumin microspheres) at 5 min intervals throughout the study. Portal pressure was decreased and collateral blood flow increased following haemorrhage ($p < 0.001$ ANOVA). Administration of vasopressin during hypovolaemia had no effect on portal pressure but collateral blood flow was increased. In contrast portal pressure increased during infusions of somatostatin and octreotide whereas collateral blood flow was decreased ($p < 0.01$). During reperfusion collateral blood flow was increased in all rats except those receiving octreotide (76.7 ± 7.3 to 40.1 ± 9.2% $p < 0.01$). The efficacy of vasoactive drugs is related to their ability to reduce blood flow through the collateral circulation including the varices. The results of this study suggest that of the three vasoactives studied, only octreotide is capable of reducing collateral blood flow during haemorrhage and resuscitation.

PO013 **REDUCTION OF LIVER TUMOUR GROWTH AND BLOOD FLOW BY OCTREOTIDE**

**N. Davies, H. Kynaston, J. Yates, S.A Jenkins & B.A. Taylor**

**Department of Surgery, Royal Liverpool University Hospital, Liverpool U.K.**

Octreotide inhibits the growth of atypical liver tumour in rats. The aim of this study was to investigate the effects of octreotide on the growth of and blood flow to experimental liver metastases derived from a colonic adenocarcinoma.

A colonic adenocarcinoma cell line (WB2054M) syngeneic to F1 hybrid rats was used to induce liver tumour. $4x10^6$ cells were injected into the portal vein of 36 F1 rats. 12 rats were treated with octreotide (2 $\mu$g bd) for 4 weeks and 12 treated with saline as a control. At 4 weeks the rats were killed and the Percentage hepatic replacement by tumour was calculated. In the remaining group of 12 rats with liver tumour, tumour blood flow was determined before and after an intravenous infusion of octreotide (0.05 ug\min for 10 minutes) using a dual radio labelled microsphere technique.

There was a significant reduction (Mann Whitney) in the percentage hepatic replacement in the octreotide treated rats, median 1.3% (range 0.6-4.7%), compared to controls 43.9% (30.1-55.2%0. There was also a significant reduction in tumour blood flow (ml\min\g) following octreotide infusion; pre 0.37 (0.1-0.97), post infusion 0.14 (0.04-0.45).

The results of this study indicate that octreotide inhibits the growth of hepatic tumour derived from a colonic adenocarcinoma. Furthermore, octreotide reduced tumour blood flow, suggesting that this may, at least in part, be its mechanism of action in inhibiting growth.

### EXPERIMENTAL LIVER METASTASES AND THE RETICULOENDOTHELIAL SYSTEM.

**N. Davies, H. Kynaston, J. Yates, S.A. Jenkins, B.A. Taylor**

**Department of Surgery, Royal Liverpool University Hospital, Liverpool, U.K.**

It has been suggested that stimulation of hepatic reticuloendothelial system (RES) activity may be the mechanism whereby octreotide inhibits the growth of hepatic metastases. In order to investigate this hypothesis, further we have assessed the effects of octreotide and gadolinium chloride (GAD), a known inhibitor of the RES, on the growth and development of heptic tumour.

Two group of 12 rats (BDIX) received intravenous GAD (5mg\Kg) and 2 groups saline (controls). All animals then received an intraportal injection of $1x10^7$ K12\Tr cells. Rats from each group received either octreotide 2$\mu$g, or saline s.c. b.d. for 4 weeks and the percentage hepatic replacement by tumour determined.

At 4 weeks the % hepatic replacement of liver by tumour in the four groups was:- saline and saline(control) median 17.5% (range 5.7-24.2), saline and octreotide 0.6% (0-2.5), GAD and saline 42% (21.2-68) and GAD and octreotide 11.2% (1.9-32.4).

These results indicate that RES blockade with GAD significantly (Mann Whitney U $p<0.01$) increases tumour growth compared to controls. Octreotide significantly inhibited tumour growth, but is more effective in the absence of GAD. It appears that RES activity is important in the growth of liver metastases. The efficacy of octreotide may be partly dependant on a functioning RES system, but other mechanisms are also operating.

PO015

### TARGETING OF 5-FLUOROURACIL TO THE LIVER:EVALUATION OF A NOVEL DISACCHARIDE ADDUCT

**N. Davies, J. Yates, H. Kynaston, B. Taylor, T. Cooke, H. Hamad,C.H. Bamford, S.A. Jenkins**

**Departments of Surgery and Bioengineering, Royal Liverpool University Hospital, Liverpool, U.K.**

Targeting of 5-fluorouracil (5FU) to the liver of patients with hepatic metastases may maximise tumour kill, minimise systemic side effects and possibly improve survival. The aim of this study was to compare the uptake of 5FU and a novel 5-FU disaccharide (galactose and fructose) adduct by normal liver tissue and hepatic tumour in rats.

Hepatic tumour was induced in BD1XZ rats by an intraportal injection of $1 \times 10^7$ K12/TR adenocarcinoma cells. Groups of rats with hepatic tumour received 5mg $^{14}$C-FU or $^{14}$C-5FU adduct (22mg) containing the same concentration of 5FU and blood samples removed at 10 min intervals. One hour after administration of the 5FU or adduct the rats were killed, the visceral organs removed, weighed and the radioactivity measured in a scintillation counter.

The hepatic uptake of 5FU and the adduct was not significantly different between the two groups of animals ($p > 0.05$ Student's t test). However, the uptake of adduct by tumour tissue was four times greater than 5FU ($4226 \pm 190$ v $1003 \pm 190$ cpm/g; $p < 0.001$). Conversely the maximal blood level of the adduct ($57.9 \pm 0.65$ cpm/ml) was significantly less than 5FU ($212.7 \pm 2.9$ cpm/ml).

The results of this study indicate that coupling of 5FU to a simple carbohydrate containing a galactose moiety selectively targets the cytotoxic to hepatic adenocarcinoma and may therefore potentiate tumour kill and improve survival.

PO016

### TREATMENT OF ADVANCED PRIMARY LIVER CANCER (APLC) BY HEPATIC RESECTION COMBINED WITH LAK CELLS ADMINISTRATION

S He, G Xu

The First University Hospital, West China University of Medical Sciences, Chengdu, Sichuan, China 610041

As of 1992 ten patients with APLC were treated by hepatic resections combined with administrations of hLAK cells and rIL-2 in our hospital, of which each course lasted 6-12 days. The hLAK cells ($1.5\text{-}2.5 \times 10^{10}$) were transfused to the patients via peripheral vein (6 cases), peripheral vein and hepatic artery (2 cases) and hepatic artery by selective catheterization (2 cases) pre-, intra- and post-operatively. rIL-2 ($2\text{-}3 \times 10^5$ units) was given IV during the course of treatment. Moreover, small doses of chemotherapy (cyclophosphamide and Mitomycin C) were used at the same time. The results indicated: up to December 1992 (over 10 months after treatment), for all of the patients, no recurrences or/and metastases were found; symptoms were relieved; liver functions improved; cellular immune functions (lymphocyte transformation rate, E rosette test and ratio of T cell subpopulations) and level of humeral immune functions (IgA, IgG and IgM) elevated to even higher values than that pre-operatively ($p < 0.01$). Reduction of AFP was observed in 8 cases. Of the 10 cases, erythema was observed in only one case. No other toxicities were observed.

Conclusions: the treatment of APLC by hepatic resection combined with administration of LAK cells was safe and feasible.

PO017 **EVALUATION OF DES-GAMMA-CARBOXY PROTHROMBIN (PIVKA-II) AND OTHER TUMORAL MARKERS IN THE DIAGNOSIS OF HEPATOCELLULAR CARCINOMA**

GL Grazi, A Mazziotti, C Legnani, R Miniero, E Jovine, A Gallucci, G Palareti, G Gozzetti

2° Department of Surgery, University of Bologna, S. Orsola Hospital, Bologna, ITALY

Screening programs for hepatocellular carcinoma (HCC) are based on repeated US scans and evaluation of $\alpha_1$-fetoprotein (AFP), but many tumors are still missed in early stages: efforts are made to improve the detection rate of HCC and thus its resectability. ***MATERIAL AND METHODS*** Over a 2 years period, 178 pts. were tested for AFP, serum ferritin (SF), tissue polipeptide antigen (TPA), carcinoembrionic antigen (CEA) and des-gamma-carboxy prothrombin (DCP) (*E-1023, Eitest Mono P-II, Eisai Co., Ltd., Tokyo*). Ninety two (51.7%) had HCC (of these 75 were cirrhotics) and the remaining 86 (48.3%) liver cirrhosis (24 pts.), liver metastases (19 pts.), benign liver lesions (19 pts.), non-HCC tumors (14 pts.) or others miscellaneous diseases (10 pts.). Effectiveness tests were assessed for each marker and crosstabulated with clinical data to evaluate differences between groups. ***RESULTS*** AFP and DCP levels were higher in the HCC group ($p<.05$). TPA, CEA and SF were not helpful in discriminate HCC. The combined use of AFP and DCP improved the sensibility rate of almost 20% (Tab. 1).

| Table 1 | Level | Sensib. | Specif. | Neg.Pred.Val. | Accuracy |
|---|---|---|---|---|---|
| AFP (ng/dl) | 20 | 50.5% | 100.% | 66.5.% | 74.5% |
| CEA (ng/dl) | 7 | 24.4% | 74.4% | 48.3% | 48.8% |
| SF (ng/dl) | 140 | 60.9% | 65.9% | 62.7% | 63.3% |
| TPA (U/l) | 95 | 69.2% | 64.2% | 68.2% | 66.6% |
| DCP (AU/l) | .09 | 53.8% | 85.9% | 63.9% | 69.3% |
| *AFP and/or DCP* | | *70.7%* | *86.0%* | *68.9%* | *78.0%* |

Sixteen HCCs (17.3%) were AFP+/DCP- and 19 (20.6%) AFP-/DCP+: in these cases DCP was of more value in non cirrhotic and in Child A cirrhotic pts. when compared with AFP ($p<.05$). The DCP sensibility rate diminished with the worsening of the liver function ($p<.05$). ***CONCLUSIONS*** DCP is as accurate as AFP as a tumoral marker of HCC, especially for pts. with preserved hepatic function, still amenable with surgery. The serum positivity for almost one of the two markers is the modality of choice in the serological diagnosis of HCC.

PO018

### BILIARY CYSTADENOMA AND CYSTADENOCARCINOMA

G Mangiante, C Iacono, F Nifosi', N Cracco, E Facci, P P Aurola, E G Serio

Istituto di Patologia Chirurgica
University of Verona, Italy

BCA and BCAC are rare,but interesting neoplasms of the liver for the possibility of the former to evolve into malignant lesion.BCA is a single round multilocular tumor:the loculs lined by a mucin-secreting epithelium with papillary projections are filled by a mucinous fluid.The presence of a cellular mesenchymal layer supporting the epithelium differentiates BCA in 2 groups:BCA with-BCMS-or without-BCWMS-mesenchymal stroma.BCMS seems to more prone to undergo malignant change than BCWMS.

BCAC shows thick papillary projections with invasion of the papillary stroma by neoplastic cells,in a fashion that closely resembles degenerated polips of the colon.Both BCA and BCAC are large and usually symptomatic tumors.For the diagnosis the most useful tools are US and CT scan,but may be difficult to differentiate BCA/BCAC preoperatively from the other cystic lesions of the liver(i.e.,solitary cyst,echinococcal cyst,cystic tumors metastases,etc. ):fine needle citology can be helpful.Between 1970 to december 1990 8 cystic biliary neoplasm(7 BCA,1 BCAC have been diagnosed at Institute of Clinica Chirurgica of the University of Verona.All but one the patients were females:the data are summarized in TAB.1.

TAB.1

| case | 1 | 2 | 3 | 4 | 5 | 6 | 7 | 8 | 9 | 10 |
|---|---|---|---|---|---|---|---|---|---|---|
| sex | F | F | F | F | F | F | M | F | F | F |
| age | 33 | 27 | 32 | 29 | 72 | 37 | 71 | 67 | 72 | 28 |
| site | LL | 4 S | 2-3S | RL | 4 S | RL | 5 S | 7 S | 6 S | RL |
| simpt | PAIN | PAIN MASS | PAIN | ABD MASS | PAIN | PAIN | NONE | PAIN | PAIN | PAIN |
| surg proc | LH | Sct | LL | RH | [redacted] | RH | VR | Sct | VR | RH |
| size | 8 cm | 13 cm | 7 cm | 18 cm | 6 cm | 14 cm | 2 cm | 6 cm | 5 cm | 14cm |
| f-up | 218m m fu | 85 m n rec | 81 m n rec | 65 m n rec | 50 m no ch | dead 38 m | 24 m n rec | 23 m n rec | 15 m n rec | 12m nrec |
| hist. | BCMS | BCMS | BCMS | BCWMS | BCWMS | BCAC | BCWMS | BCWMS | BCMS | BCAC |

LEGEND:LL left lobe,RL right lobe,S segment,LH left hemihepatectomy,LL left lobectomy,Sct segmentectomy.

The possibility of BCA/BCAC should be always ruled out in every cystic lesion of the liver:in every duobt lesion surgical exploration is indicated and at least a biopsy should be performed.

The complete excision of the lesion is the best therapeutical choice.Neoplasms can be excised with enucleation,but a correct oncological procedure require wide resection in normal parenchyma since no BCA should be considered benign before thorough hystological sampling.Only in older and poor risk patients not radical procedure,as in our case n.5,could be justified.

PO019

### BIOCHEMICAL VARIATIONS AFTER HEMIHEPATIC VASCULAR OCCLUSION FOR LIVER RESECTION IN CIRRHOSIS

L Dominioni, S Cuffari, A Benevento, L Colombo
M Besozzi, R Dionigi

Department of Surgery, University of Pavia-Varese, Ospedale Multizonale di Varese, Italy

During hepatic resection in the cirrhotic liver a prolonged interruption of liver blood flow may produce different degrees of parenchymal damage; the safe limit of duration of liver ischemia in cirrhosis has not yet been defined conclusively.
To minimize both intraoperative bleeding and biochemical disturbances after interruption of blood flow to the liver, the technique of selective hemihepatic vascular occlusion (HVO) has been developed.

AIM OF THE STUDY. To assess the variations of cirrhotic liver function occurring during and after HVO we recorded sequentially the biochemical parameters of hepatic function, including enzymes, serum bilirubin and acute phase proteins in five patients with liver cirrhosis who underwent hepatic resection for hepatocarcinoma with the HVO technique and in five patients with Pringle manouver (PM).

RESULTS. Average duration of liver ischemia was 45 $\pm$ 5 minutes for HVO technique and 32 $\pm$ 8 minutes for PM.
The biochemical alterations observed after HVO included an early postoperative increase of enzymes serum levels, which peaked on postop. day 2 and reversed by postop. day 6. A similar transient postoperative increase of serum acute phase proteins was detected, associated with a modest increase of serum bilirubin. In patients operated with PM, AST levels were significantly higher from the 1st to the 7th day; a similar pattern was recorded for bilirubin.

CONCLUSIONS. The data observed indicate that in patients with HCC in liver cirrhosis the technique of liver resection with HVO is well tolerated and causes modest and reversible changes of biochemical indices of hepatic function.

PO020

### Treatment of hepatocellular carcinoma in cirrhotic patients by chemoembolization with Mitomycin C.

F.Montalto, G.Colella, M.Tommasini,*
R.La Nocita, G.Di Tolla, R.Doci

National Cancer Institute - Milan
* Dpt. of Internal Medicine - Milan

88 cirrhotic patients affected by unresectable hepatocellular carcinoma were submitted to chemoembolization with Mitomycin C at National Cancer Institute of Milan - Italy.
In 39 pts (1986-88) we used Mitomycin C microcapsules while in 49 pts (1989-91) the treatment was performed with fluid Mitomycin C in Lipiodol suspension with or without Gelfoam.
65 were males and 23 females with a median age of 61 years (range 27-79); 72 pts were Child A and 16 Child B; 59 and 29 were Okuda I and II respectively. The median diameter of the HCC was 6 cm (range 1-13 cm). 30 pts had a single neoplastic lesion, 51 multiple and 7 had diffuse HCC.
No related treatment mortality or major toxicity was observed: only minor complications were detected (ascitis, pain, temperature).
Overall objective response was 30%: complete response in 8 pts (9%) and partial response in 19 pts (21%) with a median duration of response of 26 and 9 months respectively.
Actuarial survival was calculated: overall 1-year and 2-year survival were 58% and 44% respectively. 2-year survival of pts Child class A was 51% while 2-year survival of pts Okuda stage I was 47%.
Chemoembolization with Mitomycin C can be considered a safe and useful treatment for unresectable HCC in cirrhosis.

PO021

### SURGICAL RESECTION FOR SMALL HEPATOCELLULAR CARCINOMA

I Nagashima, S Inoune, T Nagao, N Kawano, T Muto

The First Department of Surgery, University of Tokyo, Tokyo, Japan

Surgical resection could be curative in selected patients with a single small hepatocellular carcinoma (sHCC), but the cirrhotic patients often have the risk of tumour recurrence or death from underlying liver dysfunction. Orthotopic liver transplantation (OLT) might be a rational treatment for those patients. We retrospectively reviewed twenty three patients with a single sHCC undergoing hepatic resection at our department between 1979 and 1991. 17 patients of 23(74%) were cirrhotic and the other 6 had chronic hepatitis. Three (13%) died from hepatic failure in hospital after surgery. One, 3 and 5 year survival rates of the other 20 patients were 90,79 and 61% respectively. As for prognostic factors influencing long-term survival, the presence of vascular invasion of the distance of free surgical margin (more than 1 cm or not) was not significant. The only significant factor was the severity of co-existing liver disease. One, 3,5 year survival rates of severe cirrhotic patients (Risk Score* > 2.0;n=6) were 44,22,0% respectively. On the other hand, those of the others (Risk Score < 2.0;n=14) were 100,80,69% respectively. We would suggest sHCC patients with severe cirrhosis (Risk Score > 2.0) should undergo OLT even though they might be cured by hepatic resection.

* Risk Score (RS) = $\Sigma$ (Si X Wi) / $\Sigma$ Wi

| score(Si) | 0 | 1 | 2 | 3 | 4 | weight (Wi) |
|---|---|---|---|---|---|---|
| ascites | / | 1 | 2 | / | 3 | 2 |
| Alb(g/dl) | -4.0 | -3.5 | -3.0 | -2.5 | - | 2 |
| ICGR15(%) | - | 10- | 25- | 40- | 55- | 3 |
| PT(%) | -100 | -80 | -60 | -40 | - | 2 |
| Plt($10^4$/$mm^3$) | -20 | -15 | -10 | -5 | - | 1 |

ascites; 1:none, 2:easily-controlled, 3:poorly-controlled

PO022 **FOCAL NODULAR HYPERPLASIA AND HEPATOCELLULAR ADENOMA:PREOPERATIVE DIFFERENTIAL DIAGNOSIS**

G.MANGIANTE,C.IACONO,G. PRATI,C. BENASSUTI,E. MONTRESOR e G. SERIO
ISTITUTO DI PATOLOGIA CHIRURGICA
UNIVERSITY OF VERONA ( ITALY )

We reviewed the pre-operative data on 20 cases of FNH (out of a sample of 41)and 8 cases of HCA (out of a sample of 16), investigations in all cases included US, CT, Tc-colloid and Tc-DISIDA scintigraphy, andblood-chemistry tests ( AST, ALT, ALP, BIL,CHE, GGT, AFP, CEA) . The definitive diagnosis was confirmed by histology of surgical specimens or,in the case of FNH only,by wide-wedge surgical biopsy.US proved particularly reliable in differentiating between the two forms,when the central scar,the echogenicity similar to that of normal parenchyma, and a hypertrophic supply artery to the mass were clearly discernible; these elements however, were detectable only in 3 cases. Ct diagnostic reliability corresponded to 16/20 cases of FNH as against only 2/8 cases of HCA (with a misdiagnosis of FNH in 1 case and of possible malignancy in 5). In each case of FNH, Tc-coll revealed no changes in uptake of the lesion compared to the surrounding liver, whereas Tc-DISIDA failed to show a hyperconcentration in the elimination phase in one case only. As regards HCA, Tc-coll revealed a cold lesional area in 6 cases, a low-uptake area in one case and a normal uptake area in another. Tc-DISIDA showed increased accumulation of contrast medium in the elimination phase in one case only.The perfusional phases of the scintigraphic procedures were hypervascularized in all HCA cases and in 16/20 FNH cases.Laboratory test results were normal in all cases,apart from increased GGT in 12/20 and increased ALP in 8/20 FNH cases.We observed no increases in serum tumor markers in patients with FNH and HCA.A correct pre-operative diagnosis was achievable in approximately 95% of FNH cases using imaging techniques alone,eliminating the need for surgery in the case of asymptomatic lesions.Any doubtful or suspect cases of HCA call for laparotomic diagnostic investigations and,in the case of evidence of HCA,surgical removal both on account of the haemorragic potential of the lesion and in order to obtain correct histological differentiation from a well differentiated HCC.

PO023

### INTRAARTERIAL CHEMOTHERAPY IN HEPATOCELLULAR CARCINOMA

D.Kiriakopoulou, E.Tzoracoleftherakis, S.Moschos
J. Androulakis
Surgical Unit, University Hospital, The University of Patras, Patras Greece.

Results with intraarterial chemotherapy with adriamycin (ADM) in inoperable hepatocellular carcinoma (HCC) still remain doubtful although ADM was reported as having significant activity in this disease.

Twenty-five patients with HCC were studied prospectively, regarding the quality of life and the survival following intraarterial infusion chemotherapy with ADM. Age varied from 51 to 83 years (average 62,6 years). The mean Karnofsky score was 70% at presentation. The performed catheterization was the selective intrahepatic catheterization through the gastroduodenal artery. The drug was administered via implantable port subcutaneously in the right iliac fossa, as a bolus injection of ADM 40 mg/m2 at 4-week intervals.

The majority of the patients tolerated the infusion well. Access related problems and complications of the device included: port-pocket infection 2, migration of catheter and combined duodenal fistula 1 and hepatic artery aneurysm 1. Three patients died without receiving chemotherapy. Of the remaining 22 patients, 14 showed an improvement in subjective symptoms and 14 (64%) had an objective improvement. Karnofsky score was improved in 8 (36%) patients while 9 (41%) remained unchanged. The survival rate was 64% at 6 months and 14% at 12 months. The median survival was 7.14 months (1 to 14 months). There was equal survival of cirrhotic and non-cirrhotic patients.

The application of intraarterial chemotherapy in HCC didn't show any benefit in relation to other previously reported series in terms of life prolongation but confirmed the good quality of life during the treatment and the rest of patients' lives.

PO024

### OUR EXPERIENCE IN THE MANAGEMENT OF PRIMARY LIVER TUMOURS.

E.J.Hadjiyannakis, S.Drakopoulos, R.Lakoumenda, P.Alfaras, K.T.Delis, A.Papakonstantinou, S.Raptis.

A'Surgical Dept.and Transplant Unit of the GH "The Evangelismos of Athens".

There is an increase in the frequency of liver tumours being subjected to surgical treatment in the recent years, which reflects the definitive improvements both in early diagnosis and surgical technique. With the present work we venture a retrospective analysis of the hepatectomies we performed in our department for primary liver tumours, within the period Oct.1989 - Nov.1992.

Of the total of 3785 patients hospitalized and of 3174 operated upon for all causes, we encountered 15 patients (8 females and 7 males) with primary liver tumours whom following complete preoperative evaluation, we subjected to surgical treatment. In 8 cases (53%) the tumour was rightsided, whereas in 7 subjects it was located in the left lobe. Resectability rate was 86,6% with only 2 cases deemed inoperable (intaoperatively). 13 subjects (86,6%), were operated upon electively and two under emergency conditions owing to rupture of the tumour into the abdominal cavity.

In respect to the operations, a right extended hepatectomy was performed in six cases, a left extended hepatectomy in 5 occassions and a segmental resection was decided on 2 subjects. Pathological examination of the specimens disclosed HCC in 7 cases (2 on grounds of liver cirrhosis), 5 mesenchymal neoplasms (hemangiosarcoma, leiomyosarcoma, other mixed sarcomas etc), a malignant lymphoma, a primary carcinoid tumour and a cavernous haemangioma.

Our results were satisfactory in all cases but one, where the patient succumbed to sepsis complicating extensive thrombosis of the small bowel(MR=6,6%). 14 patients were discharged from the hospital in a very good overall condition, the postoperative course ranging from 8 to 36 days (mean hospital stay 17 days).

We maintain that liver surgery produces very satisfactory results especially when both the surgical principles and resectability criteria are being well considered and applied. Unless hepatic involvement of the tumour is too great to be resected with no identifiable extrahepatic metastases, and liver grafting becomes the remaining alternative, partial hepatic resection continues to constitute the only truly effective means of treating liver tumours.

PO025

### CYTOREDUCTIVE SURGERY FOR INOPERABLE HEPATOCELLULAR CARCINOMA

WY LAU, TWT LEUNG, KL LEUNG, S HO,
N LEUNG, M CHAN, J LIN, AKC LI

Department of Surgery, The Chinese University of Hong Kong, Prince of Wales Hospital, Shatin, Hong Kong

From 1986 to 1992, a prospective study was done on 22 patients who were shown to have inoperable hepatocellular carcinoma on preoperative investigations. These patients were assessed to have good surgical risks, good liver function and no extra-hepatic spread of the disease. The tumours were inoperable because of extensive bilobar involvement of the liver. One patient was found to have an operable tumour intraoperatively and resection with curative intent was done instead.

Twenty one patients underwent cytoreductive surgery with liver resection, cryosurgery, microwave tissue coagulation and/or absolute alcohol injection. In-hospital mortality was 9.5%. One patient died of multi-organ failure 1 day after surgery because of reactionary haemorrhage which required a second operation to stop the bleeding, and another patient died 8 days after surgery because of liver failure. Other morbidity included intra-peritoneal abscess (5 patients), hyperbilirubinaemia which improved gradually (2 patients), bleeding from stress ulcers (2 patients), reactionary haemorrhage (1 patient) and chest infection (1 patient).

The symptomatic relief and quality of survival were excellent. The median survival of patients after cytoreductive surgery was 12 months and the survival was much better than those of 234 patients who received chemotherapy during the same period of the study (log rank test, $p = 0.0003$). There was no statistical difference between the survival curves of those patients who received (12 patients) and those who did not receive (7 patients) adjuvant chemotherapy or radiation therapy after cytoreduction.

We believe cytoreductive surgery contributed to the improved quality and quantity of survival in our patients.

PO026

### SURGICAL APPROACH TO HEPATOCELLULAR CARCINOMA: A SINGLE INSTITUTIONAL EXPERIENCE

G Colella, R Doci, F Montalto, M Cataldi, S Andreola, F Bozzetti, L Gennari

National Cancer Institute, Milan, Italy

From 1980 to 1992, 273 patients with hepatocellular carcinoma were observed at the National Cancer Institute of Milan. One hundred patients (80 males and 20 females) underwent hepatic resection (resectability rate 27%) of which 41 were major and 59 minor hepatic resections. HBsAg was positive in 24% of patients and AFP patients was pathologic in 50%. Concomitant liver cirrhosis was present in 49 patients and chronic hepatopathy in 25. All except three (Child B) were classified as Child A. No intra-operative death was observed. The morbidity rate was 32% (including hospital mortality 16%). Short term prognosis was mainly determined by the extension of hepatic resection, while no difference in morbidity and mortality rate was observed between the two groups of functionally normal and cirrhotic-hepatopathic livers. Excluding 16 hospital deaths, actuarial survival and free of disease survival of 84 patients at 3 years were 53% and 29% respectively. At 5 years they were 29% and 21% (median survival 37 months, median interval free of disease 16 months). Monovariated analysis with long-rank test for age, sex, HBsAg, AFP, type of surgery, cirrhosis, intra-operative blood loss, mono-plurifocality, diameter of tumour, capsule, micro and macro vascular invasion, margin of infiltration showed no significant difference for all patterns except capsule, sex and AFP. The site of relapse was hepatic in 72%, hepatic and extrahepatic in 17%, extrahepatic in 11% of patients. It is necessary to improve on the development of effective prevention and treatment against recurrent tumours.

PO027

### ENUCLEATION OF GIANT, SYMPTOMATIC HEPATIC HEMANGIOMAS WITH PRINGLE MANEUVRE: A SAFE AND BLOODLESS PROCEDURE.

F Mosca, PC Giulianotti, E Scarcello, J Romagnoli, S Signori, R. Solari, F Filipponi, G Di Candio, F D'Elia .

Institute of General and Experimental Surgery, University of Pisa; Pisa (Italy).

Hepatic Hemangiomas (HHe) are the most frequent benign lesions of the liver. Less than 1/3 of the patients with HHe are symptomatic, usually with the largest lesions referred to as "giant", (GHHe) (max diameter > 4 cm). From 12/01/87 to 12/1/92 fifteen patients, thirteen females (86,7%) and two males (13,3%), with GHHe underwent surgery: maximum diameter of the lesion was 9.3 $\pm$ 2.3 cm. Mean age of patients was 50.6 years (range 31-69). Symptomatic patients were 86.7% (13/15 pts), their major complaint being pain in right hypocondrium or epigastrium associated in 56.3 % of cases with non specific gastrointestinal symptoms. In 1/13 patient the lesion became symptomatic three years after diagnosis (initial max diameter: 6 cm preoperative diameter: 12 cm). Two/15pts with asymptomatic GHHE underwent surgery because of rapidly growing lesions. Forty percent of cases (six females) showed multiple HHe besides GHHe, their diameter being < 2 cm. Another patient showed four HHe in the right lobe, two of which were GHHe (max diameter 5 and 6 cm). All patients underwent Ultrasonography associated with AngioCT (9 pts), with Angiography (2 pts), with CT + Angiography (3 pts), with MRI (1 pt). None of the patients showed abnormality of blood tests. In 66.7% of cases simple enucleation of the HHe was performed (10/15 pts), associated in three cases with colecystectomy and in one case with colecystectomy + right adrenalectomy (pheocromocytoma). In 33.3% of cases (5/15 pts) liver resection was performed: right hepatectomy (3/15 pts), left lateral segmentectomy (1/15 pt), right lateral segmentectomy (1/15 pt). Mean blood loss (MBL) was 403.8 cc (15 pts). In the last nine cases the Pringle maneuvre was adopted, mantaining clamping for a mean time of 11 minutes (range 6-15). In this group seven enucleations and two liver resections (one right lateral segmentectomy and one right hepatectomy) were performed. MBL were 155 ml and 400 ml for enucleation plus the Pringle maneuvre ( mean diam of GHHe 9.5 cm) and resection plus the Pringle maneuvre mean diam of GHHe 12.7 cm), respectively. Total MBL (8pts) was 231.2 ml. MBL in 3 pts undergoing simple enucleation without the Pringle maneuvre was 441.5 mlMBL in 3 pts undergoing liver resection without the Pringle maneuvre was 1080 ml. Mean hospitalization of all patients after surgery was 9.8 days. Postoperative complications included one case of biliary fistula after right hepatectomy, which resolved spontaneously one month after surgery, and only minor problems (two right pleural effusions) after enucleation (2 cases). No death has occured up to date.Surgical treatment of GHHe should be limited to symptomatic and rapidly growing lesions with high risk of rupture.Benefit of resection must be outweighed against risk of operative morbility and mortality. Surgical therapy must be therefore the simplest and safest one. Enucleation is performed by digitoclasia along the natural pseudocapsular plane, clipping and sectioning afferent vessels. No biliary duct is encountered in the right surgical plane. In our experience enucleation is the first choice operation. This procedure, especially when combined with the Pringle maneuvre, is very quick, allows minimum blood loss, and is associated with a very low incidence of postoperative complications.

PO028

### SURVIVAL IN COLORECTAL CARCINOMA AND SYNCHRONIC HEPATIC METASTASES

JJ González, A Martínez, LG Flórez, J Aza

Departamento de Cirugía, Hospital Central, Universidad de Oviedo, Oviedo, Spain

The liver is a major site of metastatic spread of primary colorectal cancer.

Between January 1985 and June 1991, 810 patients with colorectal cancer were treated in our Department. 125(15.4%) of them had, at least, synchronous liver metastases, and are the object of this review. Eight patients with incomplete charts were excluded.

Sixty-six were men and 51 women. Average age was 67.21 +/- 10 (range 18-91) years. Primary tumour was localized mainly on the rectosigmoid region 53(45.3%) and rectum 31(26.5%). Liver involvement included, right lobe only, in 35(29.9%) and was bilobar in 74(63.2%) cases. There were solitary metastases in 28(23.9%) and more than four in 76(63.9%) patients.

Preoperative diagnostic work up disclosed metastases in 52(44.4%) patients, specially in the last years when the ultrasonography was developed in our hospital.

Primary tumour was resected 85(72.6%) times, while the hepatic lesions were usually untreated.

Median survival was 6 months. One and two years actuarial survival rates were 25% and 9% respectively, and only one patient lives five years later.

A significant survival advantage of patients younger than 65 years old, was noted. Resection and stage of primary tumour, normal alkaline phosphatase values, right and below four hepatic metastases are significant determinants of favorable prognosis.

Knowledgement of natural history and survival factors have therapeutic implications.

PO029 PROLONGED SURVIVAL TIME AFTER SYSTEMIC FLUOROURACIL THERAPY vs. NO TREATMENT OF PATIENTS WITH LIVER METASTASES OF COLORECTAL ADENOCARCINOMA

J.Hajdú, I.Bartha, E.Kertész[x], Zs.Kanyári, L.Bokor, A.Németh and L.Damjanovich

1st Dept. Surgery and [x]Dept. Radiology, University Medical School of Debrecen

Debrecen, P.O.B. 27 HUNGARY H-4012

Up to the recent years the treatment of liver metastases was the field of negligence in our institution. Between January 1991 and December 1992 thirtysix patients were admited to the clinic and followed up because of secondary liver disease of colorectal origin. Unfortunately 18 patients (median age 67ys, range 29-80) have had no treatment for their metastatic liver disease except for symptomatic therapy. In the same period of time in the case of an other 18 patients I.V.600 mg/m$^2$/d Fluorouracil /5-FU/ monochemotherapy was estabilished for 5 days and repeated on every 28 days. This regiment was continued at least for six month or until sings of toxicity or death. No severe side effect was observed and reduction of dose with 50% were indicated only in two cases. From the 18 patients of the chemotherapy group one underwent left sided hemihepatectomy and an another excision of two small liver metastases. In this retrospective non-randomized study the authors analysed the data of survival in the 5-FU and in the untreated group of patients, respectively. There has been no significantly differences between the age and sex distributions. From those cases having had no chemotherapy only two survived for more than 12 month, and their median survival was poor, 5 (range 2-15) month. In contrast in the chemotherapy group six patients were alive in the end of the study 6-24 (median 12) month after the tumour spred to liver had been recognised. Moreover a prolonged median survival time 9 (range 2-24) month was observed after 5-FU treatment. The difference in survival proved to be statistically significant (Student's t test, $p< 0.01$).

The authors emphasise the need of systhemic Fluorouracil chemotherapy instead of negligence to improve the survival time with better quality of life for patients suffering from liver metastases of colorectal cancer. They also consider to conduct a prospective randomized trial on larger number of cases to analyse the superiority of Ca-folinate modulated 5-FU over 5-FU alone in this metastatic disease.

PO030 **DETECTION OF LIVER METASTASES FROM COLORECTAL CANCER BY CT DURING ARTERIAL PORTOGRAPHY, INTRAOPERATIVE US AND PALPATION DURING LIVER VASCULAR EXCLUSION**

MJ Boudet, G Zeitoun, P Soyer, JM Hay, Y Flamant

Service de Chirurgie, Hôpital Louis Mourier
178, rue des Renouillers 92701 Colombes Cedex, France

Liver metastases are with local recurrence the major complications of colorectal cancer. When liver metastases occur, precise ev aluation (amount, location, size) is warranted. CT-scan during arterial portography (CTAP) seems to be the best preoperative imaging technique for detection of liver metastases. Intraoperative ultrasonography (US) has been advocated to enhance the accuracy of preoperative evaluation.
In our experience, intraoperative finger palpation of the liver when liver vascular exclusion (LVE) is performed detects with excellent accuracy liver metastases.
The aim of this study was to compare preoperative CTAP, intraoperative US, and finger palpation under LVE in patients operated on for liver metastases of colorectal cancer.

**Patients and methods**

From october 1990 to july 1992, ten patients were operated for liver metastases from colorectal adenocarcinoma; there were 7 males and 3 females, with a mean age of 59,3 ± 7,4 years (range: 43 to 70 years). Metastases were synchrone for four patients, and occurred for the others six at a mean of 27,5 ± 13,4 months after initial surgery. All patients had a simple CT-Scan and a CTAP, 8 had a preoperative ultrasonography (US) and 7 had a magnetic resonance imaging (MRI). Intraoperative US was routinely performed ; all livers were palpated before liver vascular exclusion (LVE) and 5 were palpated once again after LVE.

**Results**

Intraoperative US when compared with CTAP detected : the same metastases in 8 cases, more metastases in 2 cases, three new metastases in 2 cases. Finger palpation before LVE was able to detect these three metastases. However, 2 metastases detected by CTAP and intraoperative US were not by finger palpation before LVE. Nevertheless, these metastases were found after LVE.

**Conclusion**

The CTAP shows a greater number of metastases than other preoperative explorations. In this study, there were no false positives, and 2 false negatives when compared to intraoperative US. We showed that CTAP and finger palpation before and after LVE, gave the same results than intraoperative US. Further evaluations are however needed.

PO031

### LIVER-METASTASIZING ABILITY : ADHESION AND GROWTH OF CELLS ON CONTACT WITH ENDOTHELIAL OR HEPATOCYTE CELL MONOLAYER CULTURES

J P Bail, M T Foultier, Th Patrice

Experimental Micro-Surgical Unit, CHRU of Brest, 29200 Brest,France

The specific role of hepatocytes and or endothelial cells on the metastatic cells adhesiveness and growth remain unknown.

After passages *in vivo,* in the rodent model a colonic adenocarcinoma line (DHD/K 12-PROb - Prob- ), two variants of cells were isolated : The third selection tumour was chosen as the metastatic variant (Prob mp1); cells derived from spontaneous lung metastases bearing primary tumour isografts were re-implanted subcutaneously ("seed selection"). The second selection tumour was chosen as the liver affinity variant (Prob h2); cells derived from hepatic tumour obtained after repeated intraportal vein injections ("soil selection").

After intracaecal injections, only the seed selection tumour permitted to obtain a local tumour with liver and pulmonary metastases. The adhesion and the growth of different cells on contact with endothelial or hepatocyte cell monolayer cultures were compared; it resulted that: 1°) the growth of the tumoural cells were not dependent on the cell supports. 2°) The adhesiveness of the seed selection tumours decreases on contact with endothelial cells and the adhesiveness of the soil selection increases on contact with hepatocyte cells.

Comparison with data in the literature, our results obtained suggest that metastasis is a dynamic phenomenon and the cell adhesiveness varies with cell position to give a metastasis or not. However, the hepatocyte influence is important because the process of cell maturation along the acinus results in a matrix of varying composition.

PO032

### RESULTS OF LIVER RESECTIONS FOR COLORECTAL METASTASES. A 10 YEAR EXPERIENCE

G Gozzetti, A Mazziotti, E Jovine, GL Grazi, A Frena, A Gallucci, M Morganti

2° Department of Surgery, University of Bologna, S. Orsola Hospital, Bologna, ITALY

Results of hepatic resections for colorectal liver metastases (LM) are generally disappointing due to the high incidence of recurrence and the low survival rates. The use of new diagnostic intraoperative tools is proposed for a better patient staging and possibly improving survival. ***MATERIAL AND METHODS*** Over the past 10 years, 116 pts. have been submitted to surgery because of LM. Intraoperative echography (I.O.E.) was performed in 76 cases (65.5%). Thirty-four (29.3%) pts. were excluded from resection because extrahepatic spread (23.5%) or intrahepatic diffusion (76.4%); in 9 (34.6%) of the latter cases, exeresis was contraindicated only after I.O.E. Hepatic resection has been carried out in 82 (70.6%) pts. A total of 13 (15.9%) wedge resections, 40 (58.8%) segmentectomies and 29 (35.4%) major hepatectomies were carried out. Survivals after liver resections were computed by means of the life tables method. Uni- and multivariate statistical analysis, carried out by means of the Mantel-Cox method and the Cox proportional hazards regression model respectively, have been performed to discriminate variables related with better survival. ***RESULTS*** Only one patient out of 82 died within 30 days (operative mortality 1.2%). Thirty-seven (45.1%) pts. died during follow-up and 45 (54.9%) are alive after a mean period of 22.4 ± 3 months (ranging from 2 to 82.3). A recurrence appeared in 34 (41.4%) cases. Actuarial survivals have been 41.4% and 29% after 3 and 5 years respectively. Eight variables have showed to have positively influenced long term results: 1) absence of symptoms; 2) year of operation > 1988; 3) tumor diameter < 5 cm.; 4) parenchymal involvement < 25%; 5) use of I.O.E.; 6) segmentectomy as the operation performed; 7) CEA value ≤ 37 ng/ml; 8) SGOT value ≤ 15 U/l ($p<.05$ for each variable). Out these 8 variables, when introduced in the Cox model, only the use of I.O.E. revealed to have an independent influence on results ($p<.05$), with survival being 60.0% and 48.6% after 3 and 5 years respectively when it was use during liver resection. ***CONCLUSION*** Liver resection represents the only therapeutical choice for pts. with colorectal LM. The extensive use of I.O.E. in revealing intrahepatic occult lesions and guiding anatomical segmentectomies with a free liver margin, has led in recent years to further improve pts. survival.

PO033

### SURGICAL TREATMENT OF HEPATIC METASTASES FROM COLORECTAL CANCER.

R.Doci, P.Bignami, F.Montalto, S.Fissi, M.Cataldi, L.Gennari

National Cancer Institute
Milan - Italy

Two hundred eight patients (pts) with metastases from colorectal cancer confined to the liver underwent hepatic resection from 1980 to 1992. In 30% metastases were synchronous with primary that was removed simultaneously with metastases in 33 pts. Major resections were performed in 88 pts. The extent of surgery was in accordance with the extent of liver disease. Overall morbidity was 34% and mortality 2.4%; major complications were observed in 18% pts. Both mortality and morbidity were significantly associated to extent of resection and to synchronous intestinal surgery. In 19 pts the margin of hepatic resection was infiltrated and in 23 it was less than 1 cm. Five-year actuarial survival rate of radically resected pts was 33% and their median survival was 35 months. Disease-free survival at 5 years was 20% and median disease-free survival was 16 months. Among different prognostic factors Dukes' stage of primary ($p=.003$), percent of hepatic replacement ($p=.04$), multiplicity of metastases ($p=.01$) significantly affected survival. The first site of relapse was the liver in 43%, extrahepatic sites in 40% and combined in 17%. These results confirm the role of surgery in treatment of hepatic metastases from colorectal cancer and pick up some important prognostic factors. However, none of them should be considered as an absolute contraindication to resection.

PO034 TREATMENT OF LIVER METASTASES

J Wechsler, I Capov, J Vreticek, V Vraspir

1st Clinic of Surgery, St. Anne's Hospital,
Masaryk University, Brno, The Czech Republic

The liver is the most frequent target organ of metastases from carcinomas of the digestive tract and other organs. Possibilities of early detection followed by effective treatment improve the prospects for patients with liver metastases.

In a two-year period of 1992-93, 15 patients with metastatic liver disease (9 women, 6 men; mean age, 54.5 years; range, 26-79 years) were operated on at the 1st Clinic of Surgery in Brno. Diagnosis was made on the basis of 1) sonography (15 cases), 2) computer tomography (12), 3) laparoscopy (2), 4) enhanced CEA levels (2), 5) explorative laparotomy (1).

Tumours were synchronous in 4 and metachronous in 11 patients. Metastases were colorectal in 13 and non-colorectal (hypernephroma) in 2 patients. Of all the patients, 2 died in one month and another 2 in 3 months after operation. The extent of surgery was: hemihepatectomy, bisegmentectomy, segmentectomy, and extra-anatomical resection in 2,3,4 and 6 patients, respectively. In all of them extra-hepatic propagation of the tumour was excluded. Indications for surgery were: (i) absolute (peritonitis or hemoperitoneum after rupture of metastasis); (ii) relative (metastases of digestive tract carcinoma, endocrine tumours or hypernephroma); (iii) occasional (sarcoma, seminoma).

In future the following algorhythm of treatment in hepatic metastases will be used: 1) pre-operation cytostatic treatment to assess tumour sensitivity to cytostatics in vivo; 2) surgery (resection); 3) specific immunotherapy (production of autologous xenogeneic vaccine from tumour cells): 4) application of cytostatics to the arteria lienalis; 5) application of Interleukin 2 to the arteria lienalis.

PO035 **LIVER RESECTION USING INFLOW OCCLUSION**

JEJ Krige, PC Bornman, J Terblanche

Dept of Surgery, University of Cape Town
and MRC Liver Research Centre, Cape Town
South Africa

36 consecutive patients ( 19 women, 17 men; mean age; 48.9, range 18-72 years ) who underwent 38 hepatic resections using inflow occlusion with normothermic liver ischaemia were evaluated prospectively. Indications for resection were colorectal metastases (23), haemangioma (4), cholangiocarcinoma (3), hepatoma (3), testicular $2^{o}$, primary sclerosing cholangitis, Caroli's disease, hepatic adenoma, tuberculoma (1 each). 2 patients had repeat resections for recurrent colorectal metastases. Formal hilar dissection was used in 8 and inflow occlusion with portal triad clamping on 30 occasions. In 3 patients total hepatic isolation with caval clamping was used as well. 4 or more hepatic segments were resected on 21 occasions. Mean total inflow occlusion was 66.1 minutes, range 31-130 with <60 minutes in 14 patients, 60-90 min in 16 and >90 min in 6 patients. Mean longest single inflow period was 44.5 minutes, range 20-80. Mean operative blood loss was 1200 ml, range 150-1700 ml. 21 patients did not require a blood transfusion. In 15 patients receiving a transfusion, mean blood replacement was 2200 ml, range 500-7000ml. In 17 resections of <4 segments, mean blood loss was 692 ml of whom 2 required a transfusion. In 21 resections of 4 or more segments, mean blood loss was 1563 ml and 13 required a transfusion. 22 patients had no post-operative complications. Complications in 14 patients included bile collections (5), subphrenic collections (3), small bowel obstruction (3), bleeding (1), pneumonia (1) and urinary tract infection (1). One patient died post-operatively of multi-organ failure and coagulopathy. Mean hospital stay was 13.5 days (range 5-34 days). Significantly more complications occurred in patients receiving a transfusion (4/22 vs 8/14, $x^2=4.2$, $p=0.03$). No significant difference in the incidence of complications was found with regard to extent of resection (>4 segments 10/21 vs <4 2/15. $x^2=3.2$, $p=0.07$) age or duration of ischaemia. Inflow occlusion is well tolerated and facilitates liver resection especially in non-anatomical resections.

PO036 **A TWO CLAMP TECHNIQUE TO AVOID VASCULAR STENOSIS IN LIVER TRANSPLANT.**

J CALLEJA, JR POLO, JL GARCIA SABRIDO, J FERREIROA, E VALDECANTOS.

HOSPITAL "GREGORIO MARAÑON" MADRID, SPAIN

The most common vascular problems after liver transplant occur in arterial anastomoses. However, portal and caval anastomotic stenoses have been also described, especially in children. Starlz el al published in 1984 a technical modification in small vascular anastomosis, the so called "grow factor technique" in wich a continuous monofilament suture is tied to "a considerable distance" from the vessel wall in order to permit a maximal distension in the anastomotic line (Starlz TE, Iwatsuki S, Shaw BW Jr: A grow factor in fine vascular anastomoses. Surg Gynecol Obstet 1984, 159:164-165). The distance to which the knot is to be tied has been difficult to determine in our hands. If the suture is tied too far away from the vessel, additional stiches have to be placed to create a watertight anastomosis. We describe here a technical variation to avoid pursstring effect and anastomotic stenosis. After a countinous monofilament polypropylene suture is placed, a second clamp is applied to the donor vessels. The proximal clamp is then released allowing the distension of the anastomotic line to its maximal diameter. If the suture is then tied gently over the vessel, the blood pressure will avoid a pursestrig effect. From april 1990 to december 1992 92 adult liver transplant were done in our Liver Transplant Unit. The vascular patency was checked by sistematic echo-doppler or by angiography in cases of class III or IV malfunction, postoperative ascitis, gastroesophageal bleeding, or inconclusive echo-doppler studies. In the first 22 cases in wich the "growth-factor effect" was used we found two portal stenosis and an arterial thrombosis. In the 70 following transplants, the two-clamps technique was applied to arterial and portal anastomosis and no vascular stenosis or thrombosis were discovered to date. As the study is not prospective nor ramdomized , no statistical conclusion must be drawn, but we believe that this technique is a useful tool in termino-terminal anastomosis between vessels with elastic wall.

PO037

### LIVER TRANSPLANTATION FOR HEPATOCELLULAR CARCINOMA

K C Tan, N D Heaton, V Vougas, M Rela, E Gane, J Wendon and R Williams

King's College Hospital, London

When orthotopic liver transplantation (OLT) was first considered for patients with hepatocellular carcinoma (HCC) the indications were those tumours deemed unresectable by conventional menas. These were large tumours associated with decompensating cirrhosis. Thus it was not surprising that until recently OLT for HCC had been beset with high recurrence rates and poor results.

From past experience at King's we made a determined effort to transplant only cirrhotic patients with (HCC) <6 cm in size. Between October 1989 to December 1991 20 such patients were transplanted. They were followed until December 1992, a median follow up of 30 months and a minimal follow up of 12 months.

The aetiology were HBV in 8, HCV in 4, Alcohol in 4 and one each of alpha-1-antitrypsin deficiency and cryptogenic cirrhosis. Seventeen patients were in Child-Pugh grade B or C, only 3 were grade A. the size of the tumours were <4cm in 12 patients, 4-6cm in 6 and >6cm in 2.

Two patients with recurrent tumours died. three patients died of sepsis, 2 of recurrent hepatitis B and 1 in the immediate post-operative period of cardiac tamponade. Twelve (60%) are alive at least one year since their transplant with a median follow up of 30 months.

OLT is certainly indicated in carefully selected cirrhotic patients with small HCC.

PO038 **BILIARY ANASTOMOSIS FOLLOWING LIVER TRANSPLANTATION**

K Dawson, R Novell, A Burroughs, B Davidson, K Rolles

Liver Transplantation Unit, Royal Free Hospital and School of Medicine, London, U.K.

Methods of bile duct anastomosis were reviewed in a consecutive series of 123 liver transplants performed in 118 patients. The 30 day mortality was 12/118 (10%). Mean survival was 13 months with a range from 1 day to 36 months. Methods of bile duct repair included gall bladder conduit (N=2), primary roux loop repair (N=12), direct end to end biliary anastomosis (N=106). Indications were standard but PBC, Hepatitis B and C, alcoholic cirrhosis, cryptogenic cirrhosis and primary liver cell canceraccounted for 75% of cases. 16/106 duct to duct anastomoses were splinted with a T-tube whilst 90/106 were not. Biliary leak occured in 1/14 (7%) roux loop repairs, 4/16 (25%) splinted duct to duct anastomoses and 10 of 90 (11%) unsplinted duct to duct anastomoses. Strictures developed in 11% of unsplinted duct to duct anastomosis and no other group. Strictures occurred early and were successfully dilated ( 8 E.R.C.P., 2 PTHC). None have had to be dilated on more than one occasion and none have recurred. There is no significant difference in leak rate or stricture formation between any of the methods of repair. Direct duct to duct anastomosis is a satisfactory method of biliary repair following liver transplantation. Routine use of T-tubes to protect this anastomosis does not result in a significantly lower instance of either leakage or stricture formation. Re-transplantation and biliary leak is best treated by roux loop repair.

PO039

### EFFECTS OF TRANSPLANTATION OF HEPATOCYTES CULTURED WITH INSULIN ON ACUTE LIVER FAILURE INDUCED BY 90% HEPATECTOMY IN THE RAT

XD Wang, R Andersson, X Zhao
Depts of Surgery, Lund University, Lund, Sweden, Harbin Hospital, China

Treatment of acute liver failure is still a major clinical problem. Co-transplantation of hepatocytes with pancreatic islets have previously been demonstrated to reduce the mortality rate among rats with acute liver failure induced by 90 % hepatectomy.

In the present study, isolated hepatocytes were cultured with or without insulin for 72 h prior to transplantation and the influence on the survival rate was evaluated in rats subjected to 90 % hepatectomy. Intrasplenic hepatocyte transplantation performed 1 or 3 days before 90 % hepatectomy reduced the mortality rate. Hepatocytes cultured without insulin transplanted at the same time as the 90 % hepatectomy did not improve the survival rate. However, hepatocytes cultures with insulin, transplanted immediately following hepatectomy, significantly increased survival. An acute failure phase was defined the first 3 days following 90 % hepatectomy, followed by an acute compensatory phase during the 4th to 10th postoperative days and then a late "enhanced" phase followed.

Thus, the findings in the present study indicate that insulin may play an important role in the stimulation and regeneration of transplanted hepatocytes.

PO040 IS NEOPLASTIC GROWTH ASSOCIATED TO CHRONIC HBV INFECTION A PROGNOSTIC FACTOR OF HEPATITIS-B RECURRENCE AFTER LIVER TRANSPLANTATION?

EUROPEAN COOPERATIVE STUDY GROUP *

In order to assess whether the presence of hepatocellular carcinoma (HCC) arising on top of HBV cirrhosis is a determinant or not of viral recurrence after OLT, 170 OLT for HBV chronic infection were collected from 10 different European Liver Transplant Units *. 45 pts carried an HCC (HBV+cancer) while 125 cases were HBV cirrhosis alone. Overall HBV recurrence rate was higher in HBV+cancer group when compare to HBV group (48% vs 28%, p=0.01). Looking at the pre-OLT viral status (Tab.) the presence of neoplastic growth did not affect significantly the HBV recurrence with the exception of DNA-positive pts.

| HBV status at OLT | HBV + cancer B-rec./pts (%) | HBV B-rec./pts (%) | p |
|---|---|---|---|
| DNA - / HBe - | 9/29 (31%) | 12/46 (26%) | N.S. |
| DNA - / Delta + | 3/5 (60%) | 13/61 (21%) | N.S. |
| DNA + / HBe + | 10/11 (91%) | 7/14 (50%) | 0.03 |

An independent factor affecting HBV recurrence was perioperative anti-viral prophylaxis. In fact, in pts with high anti-HBs post-OLT titer, the HBV recurrence rate was significantly lower than in pts without prophylaxis (28% vs 65%, p=0.0005).
Furthermore, at a preliminary analysis the HBV recurrence in the transplanted liver seems to be primarily affected by pre-OLT viral replication.

*

| | | | | | |
|---|---|---|---|---|---|
| INCT | Milano | Italy | KKU | Huddinge | Sweden |
| OM | Milano | Italy | ICC | Bologna | Italy |
| ONG | Milano | Italy | UP | Padova | Italy |
| UCL | Bruxelles | Belgium | CTUK | Munich | Germany |
| CHU | Strasbourg | France | UZ | Gent | Belgium |

PO041

### ORTHOTOPIC LIVER TRANSPLANTATION (OLT) FOR SECONDARY BILIARY CIRRHOSIS (SBC) DUE TO TRAUMATIC BILE DUCT STRICTURE

M Rela, N Heaton, V Vougas, J Wendon, R Williams and KC Tan.

Department of Liver Transplant Surgery and the Institute of Liver Studies, King's College Hospital, London.

Majority of traumatic bile duct strictures (90-95%) are correctable by surgical reconstruction. However, a small proportion particularly with extensive injury associated with vascular compromise restricture despite repeated attempts at reconstruction. These patients suffer from persistent jaundice, and recurrent cholangitis and ultimately develop SBC. Biliary tract surgery in patients with SBC and portal hypertension carries a high morbidity and mortality.

We present our experience with 4 patients who had OLT for SBC due to traumatic bile duct strictures. The aetiology of the primary bile duct injury in the 4 patients were; post-operative in 2, blunt abdominal trauma in 1 and shrapnel injury in 1. All four patients had failed biliary reconstruction and three had 4 or more attempts at stricture repair. All four suffered from persistent jaundice and had portal hypertension. One patient had recurrent haematemesis due to oesophageal varices and one suffered from chronic encephalopathy (Grade 1-2). Liver biopsy in all four patients confirmed the presence of SBC.

Surgery was technically difficult due to the presence of extensive vascular adhesions and lack of tissue planes due to the previous operations. Three patients are alive and well with normal liver function tests. One patient's post-operative course was complicated by multiple duodenal perforations and she died 3 weeks post-transplant due to multiorgan failure secondary to sepsis.

OLT is a useful option in the management of patients with established SBC secondary to recurrent bile duct strictures. Conventional surgery in these patients is associated with high incidence of postoperative morbidity and mortality.

PO042

### A PROSPECTIVE, RANDOMISED, DOUBLE BLIND CLINICAL TRIAL OF HIGH SODIUM VERSUS HIGH POTASSIUM LIVER PRESERVATION SOLUTION.

S.C. Hardy, T.R. Kurzawinski, B. Fuller, A. Burroughs,
K. Cheetham, K. Rolles.
Liver Transplantation Unit, Royal Free Hospital and School of Medicine, London, U.K.

High potassium "intracellular" liver preservation solution is used to prevent ionic shifts during cold ischaemic storage. High sodium lactobionate solution (with raffinose) suppresses hypothermia-induced cell swelling and offers potential advantages in preventing potassium induced vasconstriction and hyperkalaemia on reperfusion. No prospective double blind, randomised clinical studies comparing these two solutions have been reported.

The aim of this study was to evaluate the efficacy of a high sodium liver preservation solution compared with the high potassium preservation solution used widely in clinical practice.

Thirty-six patients randomised into two groups were included in the study. Eighteen patients (nine male, nine female, mean age 44, range 22-62) were transplanted with a liver preserved with high sodium solution (Group I) and eighteen (eleven male, seven female, mean age 44, range 22-28) with a liver preserved using the standard high potassium solution (Group II). The indications for OLT were similar in both groups. The quality of liver preservation was assessed by post reperfusion liver biopsy, maximum serum concentration of bilirubin, AST, ALT and minimum platelet count within the first 48 hours post transplantation. In addition peri-operative blood loss and potassium requirements were assessed. Number of histologically confirmed acute rejection episodes were recorded and current graft survival was calculated. Mann Whitney U test was used for statistical analysis and median values are shown.

Cold ischaemic times (705 mins vs 684 mins) were similar in both groups (NS, $p>0.05$) as were the time required to fashion the three venous anastomoses before reperfusion of the liver (50 mins vs 50 mins, NS $p>0.05$). Histological evidence of damage related to preservation was present in both groups. Maximum levels of bilirubin (109 $\mu$mol/l vs 112 $\mu$mol/l), AST (944 U/l vs 596 U/l), ALT (543 U/l vs 429 U/l) within 48 hours since transplantation were similar in both groups (NS, $p>0.05$). There was no significant difference in minimum platelet count (94 x $10^{-9}$/l vs 58 x $10^{-9}$/l, NS $p>0.05$). Number of acute rejection episodes was 2.5 (range 0-4) in group I and 1 (range 1-3) in group II (NS, $p>0.05$). Current graft survival was 16 in group I and 15 in group II.

We conclude that this study has not shown any significant differences between high sodium and high potassium solutions in preservation related graft damage and long term outcome of OLT.

PO043 E.R.C.P. FOLLOWING LIVER TRANSPLANTATION

M Osborne, K Dawson, J Dooley, A Burroughs,
K Rolles, B Davidson

Hepatobiliary and Liver Transplantation Group
The Royal Free Hospital, London, UK.

From December 1988 to July 1992, 123 liver transplants were performed in 118 patients at a single centre with a thirty day mortality of10%. The preferred biliary anastomotic technique was end to end duct anastomosis without T-tube. This paper documents our experience with E.R.C.P. in post-transplant patients to investigate possible bile duct problems.

25 patients had E.R.C.P.s post liver transplant with 4 patients having two studies and 21 patients having a single study. All were performed on patients with an end to end bile duct anastomosis. Time post transplant was 5 - 560 days (median 30). The indications for liver transplantation in this group were similar to those for the whole series. 6 studies were performed as an emergency with the rest performed semi-electively on routine lists. The indications included clinical evidence of leakage or stricture, abnormal LFTs, evidence of cholangitis or ultrasound evidence of dilated ducts.

The results included failure (2); stricture (8); biliary leak (4); combined stricture and biliary leak (2) and a normal study in 9. 5 patients who had biliary leaks were converted to a roux loop anastomosis. 6 patients who had post liver transplant E.R.C.P. have died between 18 days and 16 months post-operatively and there were no complications causing significant morbidity or mortality following E.R.C.P.

E.R.C.P. is a reliable and effective way of diagnosing and dealing with biliary problems post liver transplant.

PO044 **CHOLEDOCHO-CHOLEDOCHOSTOMY WITHOUT T TUBE OR STENT (CD-CD) IN LIVER TRANSPLANTATION**

**E Moreno González, A Alvarado, I García, C Loinaz, R Gómez, I G-Pinto, , R Jiménez, V Maffettone.**

***Department of General and Digestive Surgery - Unit of Liver Transplantation, Hospital "12 de Octubre", Complutense University of Madrid, Madrid, Spain***

The biliary tract anastomosis has been considered the "Achille's heel" of the liver transplantation, with morbility rate between 8-86% in the literature. The Choledocho-choledochostomy (CD-CD) with T tube is more frequently used; however, the complications T tube related are higher. At present, some patients may be benefit of the anastomosis without T tube. Between April 1986 to November 1992, 293 liver transplants (239 adults and 49 children and 5 intraoperative deaths) had been performed. The biliary anastomosis were: CD-CD T = 143, CD-CD = 77, H-J (hepatico-jejunostomy) = 51, H-J with Stent = 14 and CD-CC-J (choledocho-cholecysto-jejunostomy) = 3. The anastomosis was performed with interrupted single layer suture with polyglactil acid (Vycril) 5/0. When used, 8-12 Fr diameter T tube, is placed. We report the complications of CD-CD T and CD-CD biliary anastomosis. The diagnosis of complications was performed by biochemical and radiological (US, HIDA and ERCP) features.

| | CD - CD T | | CD - CD | |
|---|---|---|---|---|
| | A | C | A | C |
| PATIENTS | 134 | 9 | 71 | 6 |
| - LEAKAGE | 5 | 1 | 0 | 0 |
| - LEAKAGE POST T TUBE EXTRAC. | 2 | 0 | 0 | 0 |
| - FISTULA | 1 | 0 | 2 | 0 |
| - OBSTRUCTION | 1 | 1 | 0 | 0 |
| - STRICTURE | 1 | 0 | 7 | 0 |
| - STRIC.+STONES | 1 | 0 | 1 | 0 |
| - ANAST. DEHISC. | 0 | 0 | 2 | 0 |
| - BILOMA | 2 | 0 | 2 | 0 |
| | 15 (10,4 %) | n.s 0,076 | 14 (18,1 %) | |

The CD-CD diminishes the operative time, avoid T tube complications, had not morbi-mortality advantages vs T tube use, may be delay radiological diagnosis of complications. The choledocho-choledochostomy is our first choice method of biliary reconstruction; the use of T tube in our hands is a more secure type of anastomosis.

PO045

### BRAZIL - INITIAL REPORT OF THE UNIVERSITY OF CAMPINAS (UNICAMP) PILOT LIVER TRANSPLANTATION PROGRAMME

L S Leonardi, G Berenhauser-Leite

Liver Transplant Unit, University of Campinas, Campinas, São Paulo, Brazil

Between sept. 1991 and oct. 1992, 5 orthotopic liver transplantation were performed on 5 patients.

| Tx | Date | Disease | Sex | Age Years | Total Bilirubin mg/dl | Serum Albumin. g/dl | Prothromb. time |
|---|---|---|---|---|---|---|---|
| 1 | 12/09/91 | CHA | M | 34 | 1,6 | 2,7 | 20 |
| 2 | 07/09/92 | PBC | F | 53 | 12 | 1,9 | 14 |
| 3 | 23/09/92 | PBC | F | 33 | 3,2 | 3,7 | 18 |
| 4 | 29/10/92 | PBC | F | 49 | 27 | 3,5 | 14,5 |
| 5 | 12/11/92 | CAH | M | 45 | 2,8 | 3,4 | 19,5 |

CAH - Chronic Active Hepatitis (HCV)
PBC - Primary Biliary Chirrhosis

Four patients had portal hypertension with variceal bleeding before the transplant. Two patient had important ascitis (Tx 1, Tx 5). Donor recipient matching was by size and ABO status. Standard operative techniques were used, including venous-venous bypass in 1 case (Tx 1). Average lengths and ranges of donor liver ischemia, operating time and blood replacement were 13:50 hours (range 6:18 - 24:30 hours), 16:36 hours (range 9:00 - 25:00 hours), and 24 units packed cells (range 6-45 units). Arterial reconstruction was termino-terminal in all cases. Biliary reconstruction was choledochocholedochostomy with T tube stent in 4 cases and Roux-en-Y choledochojejunostomy with straight tube stent in 1 case (Tx 5). Imunossuppression consisted of triple therapy with Azathioprine, Prednisona, and Ciclosporin. The overal biliary complications rate was 20% (1 T tube migration Tx 2). One patient (Tx 2) with previous abdominal surgery had a ilium fistula 16 days posttransplant and required reoperation (ileostomy) and dialysis for persistent renal failure during the postoperative period. 57 days posttransplant (Tx 2) this patient presented hepatic necrosis in segment VII and VIII with important hepatic disfunction without hepatic artery thrombosis. The treatment consisted in clinical support and antibiotic. One patient died (Tx 3) due to primary graft failure 2 days after the transplantation. The mean duration of Hospital stay was 32 days. Currently 4 patients are alive between 30 days and 15 months post transplant.

PO046 EARLY RESULTS OF HEPATIC TRANSPLANTATION FOR ALCOHOLIC CIRRHOSIS

B. Launois, F. Siriser, S. Landen,
E. Bardaxoglou, J.P. Campion
Department Digestive and Transplantation Surgery
Hôpital Pontchaillou, Rennes, France

The treatment of alcoholic cirrhosis by hepatic transplantation remains controversial. A balance must be found between the number of donor livers, the financial resources and the long-term benefit in survival quality of life and relapse of alcoholism. We report our experience in 16 patients (12 males, 4 females, mean age 43.5 years, range 35-58) with alcoholic cirrhosis who underwent liver transplantation between 1987 and 1991. There was a median period of follow-up of 8.3 months (range 0-34 months). All patients had a psychiatric assessment prior to transplantation and were considered preoperatively to be abstaining and suitable for surgery. Eight patients were Child A, 2 Child B and 6 Child C. There was one intraoperative death but no other hospital mortality.

At time of followup 13 patients remained alive, the three deaths all occurring in Child C patients. No patient had required a second graft. Two of the group had reverted to alcoholism on clinical and biochemical grounds. We conclude from these early results that in carefully selected patients with alcoholic liver disease hepatic transplantation is an effective treatment with reversion to alcoholism in less than 20 % of survivors.

PO047 **INDICATIONS AND COMPLICATIONS OF HEPATICO-JEJUNOSTOMY (H-J) IN LIVER TRANSPLANTATION**

**E Moreno González, A Alvarado, I García, R Gómez, C Loinaz, I G-Pinto, R Jiménez, V Maffettone.**

***Department of General and Digestive Surgery - Unit of Liver Transplantation, Hospital "12 de Octubre", Complutense University of Madrid, Madrid, Spain***

**The hepatico-jejunostomy (H-J) had the more low rate of complications in liver transplantation, and indications for use are specific. Between April 1986 to November 1992, 293 liver transplants (239 adults and 49 children and 5 intraoperative deaths) had been performed. The biliary anastomosis were: CD-CD T (choledocho-choledochostomy with T tube) = 143, CD-CD without T tube = 77, H-J (hepatico-jejunostomy) = 51, H-J with Stent = 14 and CD-CC-J (choledocho-cholecysto-jejunostomy) = 3. The anastomosis was performed with interrupted single layer suture with polyglactil acid (Vycril) 5/0. The indications are: pediatric patients (biliary atresia and biliary tract diameter incompatibility), adults patients (first choice: biliary tract diameter incompatibility, primary sclerosing and secondary biliary cirrhosis, poor anatomical local conditions and retransplantation. Second choice: CD-CD and CD-CD T complications and retransplantation). We report the complications of H-J biliary anastomosis. The diagnosis of complications was performed by biochemical and radiological (US, HIDA, PTC and ERCP) features.**

| | H - J | | H - J St | |
|---|---|---|---|---|
| | A | C | A | C |
| PATIENTS | 29 | 22 | 4 | 10 |
| - LEAKAGE | 1 | 0 | 0 | 0 |
| - LEAKAGE POST T TUBE EXTRAC. | 0 | 0 | 0 | 0 |
| - FISTULA | 0 | 1 | 0 | 1 |
| - OBSTRUCTION | 0 | 0 | 0 | 1 |
| - STRICTURE | 1 | 1 | 0 | 0 |
| - STRIC.+STONES | 1 | 0 | 0 | 0 |
| - ANAST. DEHISC. | 0 | 0 | 0 | 0 |
| - BILOMA | 0 | 0 | 0 | 0 |
| | 5 (9,8%) | | 2 (14,2%) | |
| | 7 (11.7%) | | | |

**The H-J had a low rate of complications and we use this procedure in children preferently.**

PO048

### ASCITES FOLLOWING LIVER TRANSPLANTATION IN CHILDREN

**B Dousset°, A Valverde°, D Houssin°, O Bernard*, Y Chapuis°.**

° Clinique Chirurgicale, Hôpital Cochin, Paris, France.
*Hépatologie pédiatrique, Hôpital Bicêtre, Le Kremlin-Bicêtre.

The aim of this study was to analyze the significance and the mechanisms of ascites following orthotopic liver transplantation (OLT) in children.

**Patients and methods :** From 86 to 91, 148 liver allografts were performed in 131 children, 15 (25 grafts) of whom were excluded because of early death or regrafting. Ascites was defined when ascitic fluid output was greater than 25 ml/kg/day (or >500 ml/day) and persisted at least 72 hours after removal of the abdominal drains. Group I included 31 (25.2%) allografts with post-operative ascites . Group II was the control group of 92 liver grafts without ascites.

**Results :** Ascites presented as an exsudate with a total protein level of 3.2 +/- 1.3 g/dl and a high lymphocyte cell count (669 +/- 1104/mm3), contrasting with a low lipid content. A right pleural effusion with same composition was present in 66% of the cases. Mean duration of ascitic fluid production was 25 +/-19 days. The complications of ascites included : spontaneous bacterial peritonitis (35%), respiratory complications (65%), parietal complications (50%), coagulopathy by protein loss (50%) and renal insufficiency (Creat > 130 µmol/l, 35%). There were 9 deaths (29%) in group I against 8 (9.4%) in group II (p<0.02). Prior to OLT, the predictive factors of post-operative ascites included : serum bilirubin > 300 µmol/l (p<0.02), Prothrombin rate < 30% (p<0.05) and serum albumin < 3 g/dl (p<0.05). During the surgical procedure, the use of the "piggy-back" graft implantation in case of reduced-size graft (p<0.02) and a volume of blood transfusion greater than 60 ml/kg after graft reperfusion (p<0.01) were significantly associated with post-operative ascites. Following OLT, those patients with serum bilirubin > 150 µmol/l, AST > 2500 UI/l within the first 3 days and those developing acute rejection less than 10 days after OLT were at higher risk of post-operative ascites (p<0.001, p<0.001 and p<0.05, respectively).

**Conclusions:** (1) Post-operative ascites is associated with increased morbidity and mortality following OLT in children. (2) Post-transplant ascites is an exsudate with a predominantly lymphocytic cellular content and a low lipid concentration, originating from the liver allograft. (3) Severe pre-transplant hepatic insufficiency, supra-hepatic outflow block and early graft injury (primary graft dysfunction, acute rejection) are the main mechanisms of post-transplant ascites.

PO049

### LIVER HARVESTING WITHOUT IN SITU CANNULATION OF THE PORTAL SYSTEM

J.L. Hingot, N. Rotman, M. Julien,
P.L. Fagniez, D. Cherqui

Department of General and Digestive Surgery

Hôpital Henri Mondor 94010 Créteil, France

Since the description by Starzl et al., in 1987, of the rapid technique for multiple organ harvesting, it is generally admitted that minimal preliminary dissection of intraabdominal viscus is preferable for two reasons : (a) it is associated with an improvement in graft function, and (b) it is better accepted by the hospital personnel hosting the procedure. We report here the results of a simplified technique of liver harvesting without in situ cannulation of the portal system.

From 1989 to 1992, 89 OLTs were performed in 88 patients. Nine grafts were excluded (2 cluster grafts, 3 reduced-sized grafts from a split procedure, 1 graft harvested using cardiopulmonary bypass and 3 imported grafts). University of Wisconsin (UW) solution was used for preservation in all cases. Two groups were compared. Group 1 included 20 grafts retrieved according to the Starzl rapid technique with cannulation of the aorta and the inferior mesenteric vein. Group 2 included 60 grafts retrieved with a simplified technique. The only step of the procedure specifically performed by the liver team required 5 minutes and included inspection of the graft for quality and arterial supply, and ligation of the cystic duct. The organs were only perfused by aortic route, with 3-4 liters of UW solution after cannulation of the distal aorta and clamping of the supraceliac aorta. Immediately after harvesting, the graft was perfused on the back table through the portal vein with 1 liter of UW solution.

| | group 1 n=20 | group 2 n=60 |
|---|---|---|
| cold ischemia time (hours) * | 8.3±1,2 (5-12) | 11.7±3,2 (9-24) |
| primary non function | 0 | 0 |
| retransplant | 1 (5%) | 0 |
| SGOT day 1 | 806±195 (180-3500) | 972±134 (86-3900) |
| PT day 3 (%) | 61±4 (20-100) | 57±3,7 (35-100) |
| biliary complications | 1 (5%) | 2 (3%) |
| hospital mortality | 0 | 5 (8%) |

* $p<0.01$

Conclusions : The technique reported here provides further simplification to the multiple harvesting procedure with identical graft quality despite longer preservation times.

PO050

### HEPATIC ABSCESS

M Jekić, I Jekić

Surgical Service, Clinical Hospital Center,
Zemun-Belgrade

Hepatic abscess-amoebic or pyogenic- can be diagnosed with great accuracy by either ultrasonography or computed tomographic (CT) scanning. Ultrasound is the modality of choice and will detect almost 100% of abscesses. Confirmation of a diagnosis of amoebic liver abscess is made by indirect haemagglutination test that should be positive in almost 100% of cases. Cultures of pus from the abscess and from the blood must be obtained in cases of pyogenic liver abscess. A positive culture of pus from the abscess has been achieved in 90% of cases. Ultrasound or CT guidance is utilised in aspiration of a hepatic abscess.

In the treatment of an amoebic liver abscess, Metronidazole is the amoebicide of choice. Open drainage is contra-indicated. For cases that fail to respond to therapy with amoebicides, closed drainage guided by CT or ultrasound is performed. Secondary bacterial infection of an amoebic liver abscess is an extremely rare event.

The identification and determination of the antibiotic sensitivity of organisms responsible for pyogenic liver abscess is a crucially important step. Unless a coeliotomy is necessary to correct an intra-abdominal process or the abscess is extremely large, the initial treatment of pyogenic liver abscess is a 3 week course of appropriate antibiotics followed by a 1 month course of oral antibiotics. The majority of pyogenic liver abscesses will respond to such treatment. If drainage of a pyogenic abscess is required, the preferable technique is with a percutaneous CT-or ultrasound directed catheter. Open surgical drainage should be reserved for those cases in which a coeliotomy is required for other purposes or for the patient who has failed a course of appropriate antibiotic therapy and closed percutaneous drainage is not feasible.

We have experience with 18 patients.

PO051 # CT DIAGNOSIS OF HEPATIC ECHINOCOCCOSIS

Xu Ming-Qian, Dong Zhao-Hu

The People's General Hospital of Xinjiang Uygur

Autonomous Region, Urumqi, Xinjiang, 830001, China

In all, 354 patients with liver hydatidosis were treated by surgical operation in our hospital between 1988 and 1991. One hundred and ten hydatid disease of these patients underwent CT scanning: 102 patients had cystic hydatid disease and 8 had alveolar hydatid disease.

The diagnosis of hydatid disease is based on: (1) a history of contact with dogs or sheep in prevailing areas; (2) absence of subjective symptoms in early stage of infection and the gradual occurrence of pressure symptoms with the slow growth of hydatid cyst: (3) the formation of liver abscess as a result of complicated infection of hydatid cyst and the development of acute abdominal pain and anaphylactic shock as a consequence of rupture of hydatid cyst; (4) the typical sign on palpation of hydatid cyst projecting under the liver; (5) the acoustic images displayed by ultrasonic detection: and (6) immunological diagnosis.

These procedures, however, are difficult to find early pathological changes. The use of computerised X-ray tomography in the diagnosis of hepatic echinococcosis not only makes it possible to diagnose an asymptomatic parasite-carrier but also detects accurately images of various specific pathological pictures such as solitary cyst, multiple cyst, daughter cyst, calcification of cyst, complicated infection of cyst, complicated rupture of cyst. Degeneration of cyst was revealed on the basis of observations of morphological changes of clinical pathophysiology and of evolutional dynamics of various complications.

The pre-operative diagnostic accuracy rate in this series was 99.1%.

PO052

### EXPERIENCE WITH SURGICAL TREATMENT OF HYDATID DISEASE OF THE LIVER

Xu Ming-Qian

The People's General Hospital of Xinjiang Uygur

Autonomous Region, Urumqi, Xinjiang, 830001 China

There were 1,900 patients with hydatid disease who underwent surgical treatment in our hospital during 1953.1-1992.10. Of these, 1,408 cases were liver hydatid disease, and 1,586 operations yielded 1,846 echinococcus cysts.

The methods of removal of hydatid cyst were:

1. hepatic lobectomy (4.95%)
2. removal of intact hydatid cyst (13.14%)
3. removal of hydatid cyst by puncture (81%)
4. removal of hydatid cyst by TV laparoscopy (0.91%)

Removal of the cysts left in the liver residual ectocyst cavities as large as the cysts and a bile-containing bloody effusion forming retention cyst, usually resulted in secondary infection, causing liver abscess.

The clinical observations and experimental studies of this series revealed the mechanism of intrahepatic biliary fistula formation and the natural rule of cavity closure, allowing rational cavity management and modification of operative methods:

1. closure of ectocyst cavity by suture
2. partial resection of ectocyst with cavity open
3. closure of ectocyst filling greater omentum
4. closure of ectocyst inserting catheter drainage
5. "Marsupialization" and "Roux-en-y" drainage were abandoned

This reduced the frequency of post-operative biliary fistula and of secondary infection, and also shortened the healing period.

In our series of 1,408 cases and 1,586 operations 2 deaths occurred, operative mortality 0.14%.

PO053

### SELECTIVE HEPATIC ARTERY EMBOLIZATION IS THE PRIMARY TREATMENT OF CHOICE FOR MAJOR HEPATIC HAEMOBILIA

JEJ Krige, SJ Beningfield*, J Terblanche. Departments of Surgery, Radiology* and MRC Liver Research Centre, University of Cape Town and Groote Schuur Hospital, Cape Town, South Africa.

Fourteen patients (10 men, 4 women, mean age 36.6 years, range 18-36 years) with major haemobilia originating in either the liver (13) or gallbladder (1) were treated between 1977 and 1986. Bleeding was due to penetrating (4) or blunt (1) liver trauma, iatrogenic (3) [percutaneous transhepatic endoprosthetic stent placement (2) liver biopsy (1)], arteriovenous malformation (2) right hepatic artery aneurysm (2), pyogenic liver abscess (1) and chronic cholecystitis with gallstones (1). All patients had melaena, 5 had haematemesis and RUQ pain was present in 8 patients. Only 2 patients were jaundiced. Bleeding from the ampulla was identified in 2 patients during ERCP. Endoscopy identified fresh blood in the second part of the duodenum in 7 of 10 occasions. A liver lesion was identified in 6 of 10 patients who underwent either CT scanning or liver ultrasound. Selective hepatic angiography demonstrated an intrahepatic bleeding source in 13 patients. An arteriobiliary fistula in the gallbladder in 1 patient was not identified by angiography. Selective hepatic arterial embolization using either gelfoam pledgets or Gianturco coils controlled bleeding in 10 of 12 patients. Embolization failed in 2 patients (1 with segmental liver necrosis required a right hepatic lobectomy and a second patient underwent surgery and ligation of the left hepatic artery). Bleeding from the gallbladder in 1 patient was treated by cholecystectomy. Selective hepatic artery embolization was not attempted in 1 patient who underwent a left hepatic lobectomy. Selective hepatic artery embolization was successful in 10 of 12 patients (83%) of whom 1 patient developed subsequent complications.

Selective hepatic artery embolization provides definitive control of liver bleeding with a low incidence of complications and should be considered the primary treatment of choice for intrahepatic haemobilia.

PO054

### BILIARY FISTULA IN THE LIVER HYDATIDOSIS: COMPLICATION OF THE CONSERVATIVE SURGERY.

J.M. Jover; M.A. Delgado; J.C. Ruiz; J.L. Ramos; J. Alvarez; R. F. Lobato; M. Moreno.

Hospital Universitario de Getafe.

Madrid. Spain.

For many years hydatidosis has been considered a benign disease and therefore treated by conservative surgery nor always followed by good results.

The biliary fistula is often a complication of the conservative surgery and involve an inadequate treatment because implies removal of the contents leaving pericystium in situ.

PATIENTS AND METHODS. Three females are presented in this poster (62, 63 and 73 year-old). They had been treated out of our Department, 12 years, 4 and 6 moth ago. The first surgery was conservative in all cases (2 tube drainage and 1 partial cystopericystectomy) and the cyst was located in the right lobe.

Patients arrived to the emergency with fewer, acute colangitis and external biliary fistula. One also showed extracellular fluid volume deficit, metabolic acidosis and hipokalemia.

The diagnosis was completed by ECO, TAC and fistulography.

In all patients was necessary to complete the cystopericystectomy and the biliary fistula was isolated and ligated on hepatic tissue. Intraoperative studies with x-ray were done routinely. In a patient a sphinteroplasty was associate.

One patient died with sepsis and two remains asyntomatic.

CONCLUSION. The resective procedures give the best short and long term results in the treatment of multivesicular hydatidosis. The biliary fistula always involve an insufficient treatment and may be cause of serious complications. The treatment of choice is the close on normal hepatic tissue.

PO055

### THE USE OF VENOUS ALLOGRAFTS IN DIGESTIVE SURGERY

S Landen, G G Jamieson, B Launois

Centre for Digestive Surgery, Hospital Pontchaillou, France and Department of Surgery, Royal Adelaide Hospital, Adelaide, Australia

The use of artificial conduits (Gorotex, Dacron) as interposition or bypass grafts in the portal venous system is associated with high rates of early thrombosis. The use of homologous veins (e.g. the internal jugular vein) adds a further operation to what is already a long procedure. Preserved iliac veins, harvested from organ donors, are used infrequently in graft recipients and so provide a ready supply of venous allografts for use in other circumstances. We have used such allografts in 6 patients, four for replacement of a portal vein resected en bloc with an invading cancer (pancreatic cancer : 2, bile duct cancer : 2, all with partial liver resection) and two for complicated cases of portal hypertension. The length of preservation time for the vein graft prior to insertion ranged from 1 - 29 days with a median of 6 days. No patient was placed on immunosuppression and all patients survived operation and left hospital. In two patients, the graft apparently functioned initially but later thrombosed. In the remaining patients the clinical course suggests that the grafts have remained patent. The longest follow up at present is 13 months. If venous allografts used without immunosuppression prove to retain patency over a long term then the implications for the use of such veins are many and varied.

PO056

### DIFFUSE-FORM CAROLI'S DISEASE. A SURGICAL DILEMMA

S Landen, G J Maddern, J P Campion, M Gosselin
B Launois

Centre Hospitalier, Universitaire de Rennes, France

We report the case of a 31 year old male patient in whom the diagnosis of diffuse-form Caroli's disease associated with congenital hepatic fibrosis was made at age 12, when the patient developed his first attack of acute pancreatitis due to migration of intrahepatic bile duct calculi. Following endoscopic sphincterotomy, the patient remained asymptomatic for 5 years. In August 1990, he developed acute angiocholitis with ensuing septic shock and acute respiratory distress syndrome. At laparotomy, all superficial liver cysts were fenestrated in order to remove intrahepatic bile stones. Bile duct anomalies in the left liver lobe were minimal, but the idea of performing the right hepatectomy was abandoned because of the small size of the left lobe and the existence of associated congenital hepatic fibrosis. Resection would almost certainly have led to acute hepatic failure and encephalopathy. The patient is alive and well 8 months post-operatively, but remains at risk of developing life-threatening angiocholitis.

Caroli's disease, a rare condition with an incidence of 1 per million, is characterised by congenital dilatation of the intrahepatic bile ducts. The bile duct anomaly may, in 20% of cases, be limited to a liver segment or lobe, but in it's usual form, it affects the entire intrahepatic biliary tree. In the latter form, biliary drainage techniques have often proved ineffective in preventing recurring bouts of angiocholitis. Moreover, resection of the most severely affected liver parenchyma is seldom feasible because of associated congenital hepatic fibrosis or secondary biliary cirrhosis. In such cases, OLTX (orthotopic liver transplantation) may represent the only treatment, but optimal timing for this procedure has yet to be determined. In these typically young and otherwise healthy individuals, OLTX may be perceived by the patient and his practitioner, as an unacceptable burden. Later however, if acute angiocholitis resistant to antibiotics develops, the patient may no longer be suitable for liver transplantation.

PO057

### THE TECHNIQUES OF THE NORMOTHERMIC AND HYPOTHERMIC TOTAL HEPATIC VASCULAR EXCLUSION FOR LIVER SURGERY

J F Huang et al

Department of Hepatobiliary Surgery, First Affiliated Hospital, Sun Yat-Sen University of Medical Sciences, Guangzhou, China

The improved technique for bloodless hepatic resection using the total hepatic vascular isolation under the normothermic or hypothermic condition were reported to deal with the large hepatoma and the severe liver trauma involved in the liver hilum, the main hepatic veins and retrohepatic inferior vena cava. The original Heaney's or Fortner's methods were modified so that the improved techniques could be simpler and more practicable to perform otherwise hazardous liver resection.

Over 4 years, major hepatic surgery was successfully performed on 20 occasions with the normothermic or hypothermic total vascular exclusion techniques in our department for patients with malignant and benign liver tumour or trauma. Among 20 cases, 17 with the normothermic selective total vascular exclusion (extended right lobectomy: 5 cases; extended left lobectomy: 2 cases; right lobectomy: 5 cases; central segmentectomy: 3 cases; repair of the ruptures of the hepatic veins and the retrohepatic vena cava: 2 cases); 3 with the total vascular isolation on the in situ cold perfused liver (left trisegmentectomy 1 case, right trisegmentectomy:1 case; extended left lobectomy 1 case). The indications and the clinical experience of the application of the techniques were discussed. They may increase the repertoire of the surgeon in the management of a variety of hepatic lesions.

PO058 *BENIGN TUMORS OF THE LIVER. DIAGNOSTIC AND THERAPEUTIC APPROACH.*

*B. PRADERE, E. BLOOM, CH. JULIO, P. MAQUIN.*

*C.H.U. PURPAN - Place du Dr BAYLAC*

*31059 - TOULOUSE Cedex - FRANCE*

This study concerning 31 benign tumors of the liver was aimed to point out the pitfalls in the diagnosis and treatment of such tumors.
From 1986 to 1992, 31 benign tumors of the liver were diagnosed in 29 patients, 25 women and 4 men. These tumors were 15 hemangiomas, 12 focal nodular hyperplasias, and 6 adenomas. Six of the hemangiomas were symptomatic, as well as 3 of the focal nodular hyperplasias and 1 of the adenomas.
The diagnosis was assessed on behalf of imaging techniques: ultrasound (23), C.T. scan (26), M.R.I. (13), arteriography (7). A liver biopsy was performed in 9 patients.
In 11 patients, such diagnosis data were sufficient to avoid surgery in asymptomatic hemangiomas and focal nodular hyperplasias.
The remaining 18 patients underwent surgery. Preoperative diagnosis was confirmed in only seven patients (39%): 4 hemangiomas out of 6, 1 focal nodular hyperplasia out of 8 and 2 adenomas out of 4. Surgical procedures were 5 major hepatectomies, 3 bisegmentectomies, 7 segmentectomies and 2 tumorectomies.
This retrospective study enhances the difficulties encountered to assess an exact pre operative diagnosis in benign tumors of the liver.

PO059

### OUR EXPERIENCE IN THE MANAGEMENT OF NONPARACYTIC CYSTS OF THE LIVER

G.V.Velmachos, C.Kindylidis, G.Svoronos, A.Mystakidis, G.Garinis, N.Kitromilis, N.Lambrinaki, V.Mouzakis

Surgical Unit, SOTIRIA Gen. Hospital of Athens, Greece

Nonparacytic cysts of the liver, congenital or acquired, are relatively rare and to a great degree they arise asymptomatically.
The present study is an account of our experience in the management of our cases. The methods of present-day diagnosis and treatment are also reviewed.
Fourteen patients (4 males and 10 females) were hospitalized for nonparacytic cysts of the liver during the years 1986-1991. The age ranged from 36-76 years (mean 58.4 years). Almost all patients complained of moderate heaviness in the epigastrium or the right hypochondrium. In 5 patients (35.7%) indigestion and a history of duodenal ulcer co-existed; 1 patient had mild jaundice without fever. The blood tests, microbiological and biochemical examinations, mainly those concerning liver function, were within normal limits except one patient who had moderate diabetes mellitus. The laboratory tests for hydatidosis were negative.
Ultrasonography, computerized tomography, retrograde cholangiography and liver scanning were an essential diagnostic tool in 10 of our patients. In the remaining 4 patients, the cysts were an incidental finding. Localization of the cysts in 8 patients (57.2%) was in the left lobe and in 4 (28.6%) the cysts were found bilaterally. In 9 patients (64.2%) the cysts were solitary and in 5 patients (35.8%) multiple; their diameter ranged from 0.5-7.5 cm. Radical cystectomy was carried out in 8 cases (57.3%), marsipulization in 1 (7.1%), atypical left hepatectomy in 1 (7.1%), capitonage in 2 (14.2%) and partial Lin-Chen-Wang cystectomy in 2 (14.2%). Cytology of the cystic fluid revealed a small number of RBC's, histiocytes, some elements of bile and rare hepatic cells. Histology revealed features of nonparacytic cysts. Mean hospitalization was 11.4 days (7-22) and mortality was nil.
In concluding it can be stressed that the treatment of choice is radical cystectomy with or without hepatectomy, while in the cases in which the liver is polycystic, the Lin-Chen-Wang procedure is suggested.

PO060 ECHINOCOCCAL CYSTS

G Di Gioia, V Berta, S Cruciani,
C Raviolo, A Guarneri

Surgical Unit Stefano Garberini
San Carlo Borromeo Hospital, Milan, Italy

We review the cases of hydatidosis observed since 1967 up today. 48 patients (28 f and 20 m) presented hepatic hydatidosis, 7 patients (4 f and 3 m) had pulmonary location, 1 patient (f) had both and 1 (m) presented splenic hydatidosis. Ultrasonography and CT were main instrumental diagnostic procedures. Surgical radicality (ablation of cyst and pericystium) and conservation of hepatic and pulmonary tissue have been our aims. In hepatic hydatidosis we performed 14 total and 5 subtotal pericystectomies. 12 regulated hepatic resections and 3 pericystoresections of Bourgeon have been carried out when massive occupation of hepatic parenchyma and hypertrophy of residual tissue were present. In conservative operations, performed when not possible pericystectomy and not indicated a major resections, we report 6 partial cystic resections with open residual cavity and drainage, and 3 cystic-jejunostomy on a Roux-en-Y. Conservative surgery needs sterilization of cystic cavity, to this purpose we preferably use H2O2. In the postoperative course, we observed a higher rate of early morbidity in conservative methods than in radical ones, being complications represented by local infection, biliary fistula and cholangitis. The average hospitalization time was 13 days for radical operations and 17 days for conservative ones. However, the follow up has not shown significant differences in relapses between radical and conservative techniques. In pulmonary hydatidosis we carried out 3 wedge resections and 3 lobectomies. One patient presented multiple location, hepatic, pulmonary and splenic, treated in three different times: 1° pericystoresection of Bourgeon, 2° inf. left lobectomy and splenectomy, 3° pericystectomy for cystic relapse to the left lung. At last, we report one case of splenic hydatidosis as the only location treated with splenectomy. In conclusion, our results indicate that the operative choice for echinococcal cysts must be balanced by the aim at radicality on one hand, and by saving parenchyma on the other. In hepatic hydatidosis, pericystectomy is the first choice operation, but when multiple cysts are present, partial cystic resection and drainage, previous sterilization of cystic cavity could represent a good alternative to more aggressive hepatic resection.

PO061 RECEPTEURS HORMONAUX ET TUMEURS DU FOIE

B. DESCOTTES, D. VALLEIX, E. OSTYN,

J.C. VANDROUX, G. CATANZANO

Service de Chirurgie B - CHU -
2 avenue A. Carrel 87042 LIMOGES CEDEX

De 1988 à 1991, 105 résections hépatiques ont été réalisées pour des lésions différentes :

- métastases,

- adénomes,

- hyperplasies nodulaires,

- hémangiomes.

Les éxérèses ont consisté en :

- 64 hépatectomies droites,

- 16 hépatectomies gauches,

- 25 hépatectomies atypiques comportant 2 ou 3 segments.

Au cours de chaque éxèrèse, les marqueurs suivants :

- récepteurs à l'oestradiol,

- récepteurs à la progestérone,

- cathepsine,

- protéine S2, ont été dosés sur le foie sain et sur la lésion tumorale, bénigne ou maligne.

Les résultats préliminaires de cette étude montrent que :

- les récepteurs à l'oestradiol ne sont par différents dans le foie sain ou dans le foie tumoral,

- les récepteurs à la progestérone sont augmentés dans les métastases par rapport au foie sain et très nettement diminués dans l'adénome, mais le nombre des patients ne permet pas encore d'apprécier une différence significative,

- la cathépsine est augmentée dans l'adénome de façon non significative, par contre elle est diminuée de façon significative (P < à 0,03) dans les métastases hépatiques,

- enfin, la protéine S2 est nettement augmentée dans les métastases par rapport au foie sain ; là-encore, il faut attendre un plus grand nombre de patients pour pouvoir envisager une différence significative.

Les auteurs, au vu de ces résultats, essaient d'apporter une explication à l'enregistrement des modifications de ces différents récepteurs dans certaines pathologies tumorales.

PO062

### BRAZIL - THE POSTERIOR INTRAHEPATIC APPROACH FOR HEPATECTOMY. INITIAL EXPERIENCE AT UNIVERSITY OF CAMPINAS

L S Leonardi, J C Pareja, G Berenhauser-Leite, S C P Leão

Department of Gastroenterology Surgery, University of Campinas, Campinas-SP, Brazil

Recently was described a technique (1) for approaching the hepatic pedicle structures and their branches by an intrahepatic posterior approach that allows early delineation of the segments of the liver without the need for extrahepatic pedicle structures dissected separately.

We used this technique during the second semester of 1992, in 6 patients (table). This technique allows the surgeon to dissect and clamp the required sheath early in an operation, eliminating any guesswork involved in defining the boundaries of the segment (S) to be removed. This approach can be used in partial hepatectomy and for resections of tumours of the proximal bile duct.

We believe also that this technique is one of the factors that leads to diminished blood loss in hepatic resections. Certanly, it is a useful addition to the armamentarium of operations upon the liver.

| Case | Age | Sex | Disease | Surgery | time (h) | blood | Compl. |
|---|---|---|---|---|---|---|---|
| 1 | 60 | F | Metastasis | S.IVa + b | 6 | 5 | - |
| 2 | 66 | F | Giant Cist | R.H. | 6 | - | - |
| 3 | 57 | F | Tumour | S.IVb + V + VI | 6 | - | - |
| 4 | 37 | M | B.D.E. | H - J - A | 4 | 3 | - |
| 5 | 50 | M | Klatskin | L.H. + H-J-A | 9 | 1 | - |
| 6 | 64 | F | Klatskin | S.IVb + H-J-A + BII | 13 | 2 | W.I. |

Time = Operating time; Blood = Units packed cells;
S= Segmentectomy; R.H. = Right Hepatectomy; L.H. = Left Hepatectomy;
H-J-A = Hepaticjejunoanastomosis; BII = Billroth II Gastrectomy;
W.I. = Wound Infection; BDE = Bile duct estenosis

(1) Launois,B.; Jamieson,G.G. - Surg.Gynecol.Obstet., 1992, 174: 7-10.

PO063 A Clinical Trial Of A New Insulin Delivery Method

Osamu Sato, Takeaki Shimizu, Fujio Sugimoto, Tetsuya Takasawa

Department of Surgery, Shinrakuen Hospital, Niigata, Japan

A new technique of insulin delivery using the greater omentum was designed to exert greater metabolic effects for the patients with liver dysfunction. This new method was easy, simple, and safety as follows : The catheter using for insulin administration was inserted into the abdomen and the catheter tip was attached the greater omentum under laparotomy. A continuous infusion of insulin using syringe pump was underwent during operation. Since it might be questioned whether the omental route is suitable for insulin administration, a clinical examination was performed in 4 cases who were received colectomy as follows : The examination was a voulus administration of 20 U human regular insulin using our method. Peak levels of insulin in portal blood were achieved 5 to 15 minutes, and those were twice levels of peripheral blood. A prospective randomized trial of insulin administration in 15 patients received hepatectomy. The patients were divided into two groups : In group A, 2 to 4 units per hour of insulin were administrated using our method, and group B was not administrated. During the operation, average minimal value of arterial blood keton body ratio (AKBR) in group A was more than 0.7, but that in group B was less than 0.7. Conclusion : 1.A new method of insulin delivery using the greater omentum was simple, safety, and effective. 2.In hepatectomy to the patients with liver dysfunction, our method was useful at the aim of inhibitory effect on the suppression of AKBR. The author believes that a new technique of insulin delivery using the omental route is widespread and has proposed ways of application to the treatment of diabetes mellitus.

PO064 **THE NEW APPROACH OF MAJOR HEPATECTOMY**

E. I. Galperin, S. R. Karagulyan,

N. F. Kuzovlev, A. Tchevokin

Department of Liver Surgery of Moscow Medical Sechenov Academy

Our experience of 123 hepatectomies (HE) demonstrated that the volume of operative blood loss was one of the most decisive factor in determining prognosis in patients undergoing hepatic resection. We have elaborated a simplified technique for the exposure of hepatic pedicle (HP) and their branches by an intrahepatic finger approach (IFA). IFA consists of successive stages: 1) a superficial incision of Glisson's capsule at a point related to external landmark; 2) finger intraparenchymatous exploration for a large trunk internal landmark; 3) hooking the HP and drawing it out the visceral surface of the liver; 4) correcting the position of the clamp preliminary placed on the HP according to the borderline of liver ischemia. The IFA from the visceral surface of the liver allows the surgeon to dissect and clamp the required sheath in the beginning of the operation, it identifies clearly the anatomical segmental boundaries and minimizes the amount of devitalized hepatic parenchyma. Additional reducing of the blood leakage was achieved by using an ultrasound dissector "Dissectron" (Satelec, France). This method has been used in 16 major HE, allowing to reduce general liver ischemia to 1,5-2 minutes to perform HE with a mean blood transfusion requirement of 1 units. No transfusion was required in 4 of the 16 patients.

PO065

### CAVAL THROMBOSIS AND PULMONARY EMOBLISM COMPLICATING AN AMOEBIC LIVER ABSCESS

S Aman, M Fuad, I Mousa, F Abu-Zidan

Department of Surgery and Radiology, Mubarak Al-Kabeer Hospital, Kuwait

A thirty year old Indian male presented with abdominal pain, fever and jaundice of 3 days duration.

Ultrasound and CT scan of the abdomen revealed a hypo-echoic lesion of the caudate lobe of the liver of 5 cm in diameter. Indirect haemagglutination test showed very high titre. The patient was treated with intravenous Metronidazole and he had a reasonable clinical response. 4 days later, he developed fever, cough and chest pain. Repeat abdominal ultrasound revealed the presence of IVC thrombosis with an abscess of 4 cm diameter compressing the cava. Lung scan showed pulmonary embolism. The proposed cause of thrombosis is compression of IVC and extension of the inflammatory process.

Early aspiration of amoebic liver abscess of the caudate lobe is recommended in spite of the size of the abscess or the clinical response to medical treatment.

PO066 A MODEL OF CHOLESTASIS IN THE RAT USING A MICROSURGICAL TECHNIQUE

MA Aller, L Lorente, S Alonso, J Arias

Surgery I Dept. Hospital Universitario San Carlos. Complutense University. Madrid.Spain

An experimental model of extrahepatic cholestasis in the rat, using a microsurgical technique, is described. Sixteen days post-operatively all of the animals (n=10) were alive and had hepatomegaly, splenomegaly, splenorenal collateral circulation, jaundice and hyperbilirubinemia. An increase in the size of the portal spaces, with intense proliferation and a slight infiltrate of inflammatory cells, was observed. The proliferation of the bile ducts projected out of the portal spaces, invading the hepatic trabecula, and connected the spaces together.
The use of this technique prevents the development of hepatic cysts and other complications inherent in the surgical techniques of cholestasis, such as hepatopneumonic abscesses. Because of the lack of postoperative mortality and the non-existence of hilus biliary cysts, the microsurgical technique described for the obstruction of the extrahepatic biliary route in the rat could be a useful experimental model for the study of cholestasis in the early stage postoperatively and for secondary cirrhosis.

PO067

### CHOLEDOCHAL CYSTS IN ADULTS

**PM Hewitt, JEJ Krige, PC Bornman, J Terblanche.**

**Department of Surgery, Groote Schuur Hospital, University of Cape Town, Cape Town, South Africa.**

Choledochal cysts presenting after childhood are uncommon. Clinical features and results of treatment in 14 adults who presented between 1979 and 1992 were evaluated. The 10 women and 4 men had a median age of 26 years (14-62 years). Diagnosis was delayed (>1year) in 7 patients and missed in 2 who had biliary surgery elsewhere. In most cases symptoms were nonspecific. One patient presented with cyst perforation secondary to worm obstruction of the common bile duct (CBD) and another had pancreatitis. Of 13 patients who underwent cholangiography, 8 had the characteristic common-channel between CBD and pancreatic duct. Two had associated cholangiocarcinoma. Nine of 10 patients with Todani type-I cysts underwent cyst excision (leaving the posterior adventitial wall intact - Lilly technique) and Roux-Y hepatico-jejunostomy. One with malignancy was treated non-operatively. Three patients with type-IVa cysts had extrahepatic cyst excision with hepaticocyst-jejunostomy. Simple removal was performed in 1 case of a type-II cyst. There was no surgical mortality.

Median follow-up was 5.5years (6 months-9 years). Three patients developed hepatico-jejunostomy strictures 2, 4 and 40 months after definitive surgery: 1 had a left hepatic lobectomy after failed percutaneous removal of intrahepatic stones and another underwent revision hepatico-jejunostomy. A third patient developed sclerosing cholangitis with secondary biliary cirrhosis and in spite of revisionary surgery, died 8 years later from hepatic failure. Both patients with cholangiocarcinoma have died. Of the 11 survivors, 10 are currently well and 1 was lost to follow-up after 3yrs.

**Conclusion:** Diagnosis was delayed in most adults with choledochal cysts. Total cyst excision using the Lilly technique with Roux-Y hepatico-jejunostomy, is the treatment of choice and in the absence of cholangiocarcinoma, results are favourable. Where complete excision is impossible (type IVa) hepaticocyst-jejunostomy is recommended, but the risk of malignant transformation in the residual cyst remains uncertain.

PO068

### REDUCTION OF E. COLI ADHERENCE TO T-TUBE SLICES BY PHOSPHOLIPID TREATMENT

JL Yu, R Andersson, Å Ljungh*,

LQ Wang and S Bengmark

Departments of Surgery and Medical Microbiology*,
Lund University, Lund, Sweden

One of the main complications in the use of biliary stents is the blockage formation due to bacterial adherence. The present study aimed at modifying the surface of biliary drainage in order to reduce bacterial adherence.

T-tubes, used as a representative of biliary drain materials, was cut longitudinally into slices with 1 $cm^2$ of square surface. The slices was treated with phosphatidylcholine (PC) and phosphatidylinositol (PI) by soaking them in phospholipid-chloroform solution for 4 minutes and drying at 60°C for 24 hours at Karlshamns LipidTeknik AB (Stockholm, Sweden) and sterilised by gas at Grambro Lundia AB (Lund, Sweden. Control slices were sterilised at 120°C for 30 minutes. The adherence of cells of seven *E. coli* strains to the native T-tube slices, T-tube slices with PC or PI were studied *in vitro*, and the adherence of cells of *E. coli* strain NG7C to implanted PC- or PI-treated slices from the common bile duct in the rat. The T-tube slices were incubated with 1 x $10^7$ cfu radiolabeled *E. coli* cells/ml at 37°C for 60 min and then drained and washed thrice in 2 ml phosphate buffered saline (PBS). Adherent *E. coli* cells were quantified by radioactivity counting.

Both PC and PI adsorbed on the surface of slices prevented against adherence of *E. coli* cells of all seven strains, whereas, after implanted into the common duct in rats between 6 and 12 days, phospholipid-treated slices partially prevented bacterial adherence. Additional studies showed that phospholipid-treated slices inhibited fibronectin to deposit on the surface.

In conclusion, the results suggest that the phospholipid-treated T-tube slices reduce the adherence of *E. coli* in at least two ways, i.e. by changing surface properties *in vitro* and by reducing deposition of host-derived molecules on the phospholipid-treated surface *in vivo*. The results may be of benefit for the modification of biomaterial surfaces in the clinical situation.

PO069

### EXPERIMENTAL FOREIGN BODY INFECTION IN THE BILIARY TRACT IN RATS

JL Yu, R Andersson, LQ Wang,
Å Ljungh* and S Bengmark
Departments of Surgery and Medical Microbiology*,
Lund University, Lund, Sweden

Stent blockage and subsequent cholangitis impair its increasing use. The study aimed at evaluating the role of biliary biomaterial in infection associated with implants in the biliary tract.

T-tubes, used as a representative of biliary drain materials, were cut longitudinally into the slices with 1 $cm^2$ of square surface each. The T-tube slices were sterilised at 120°C for 30 min before used. After 5-day period of common bile duct occlusion by the use of a minioccluder surrounding the common duct followed by 7-day relief of the occlusion, male Sprague-Dawley rats were divided into three groups. Group A (n=27) was used as controls. Group B (n=36) underwent T-tube slice implantation into the dilated but patent common duct, while the animals in group C (n=27) underwent sham implantation. Between one and four weeks after implantation, the animals were challenged with an inoculum between $10^2$ - $10^5$ cfu of *E. coli*(clinical isolate) in 100 µl NaCl into the biliary tract. Spontaneous bacterobilia were identified by bile sampling before inoculation and routine bacterial cultures and animals with positive cultures were excluded from the study. Complement-mediated opsonic activity in bile and sera was analysed by use of a phagocytic bactericidal assay.

Neither positive blood culture nor spontaneous bacterobilia was found. Bile was sampled and bacterial culture were performed within 24 hours after inoculation. $10^2$ cfu injected into the common duct produced biliary infection in 34/36 animals in group B, but only in 6 of 27 animals in Groups A and C ( $p<0.01$, Fisher's exact test). $10^5$ cfu resulted in 100% bacterobilia in groups A and C. Bacterial counts in bile in Group B was 2 x $10^8$ cfu/ml(n=34) 24 hours after inoculation of $10^2$ cfu, whereas 6.3 x $10^4$ cfu in infected animals in Group A and 4 x $10^4$ cfu in Group C. Culture 72 hours after inoculation showed, that the bacterial number in bile in groups A and C significantly decreased, whereas it remained high in Group B. Complement-mediated opsonic activity in bile in Group B decreased gradually with time(24±2.8 before implantation vs. 6±2 four weeks after implantation, $P<0.01$) whereas opsonic activity in serum in all groups, and in bile in Groups A and C remained the same level as initially.

Our results suggest that, impaired local defence in bile may at least partly play an important role in bacterial infection associated with implants in the biliary tract.

PO070

### A SIMPLE PREDICTIVE SCORE FOR CHOLEDOCHOLITHIASIS: A PROSPECTIVE EVALUATION

T Montariol, C Rey, M Veyrière,

Abe Fingerhut and Association for Surgical Research of Ile de France (ARCIF)

Pre-operative selection of patients with a low risk of choledocholithiasis, with the intent of avoiding intra-operative cholangiography (especially during laparoscopic cholecystectomy), was recently proposed by Huguier and Lacaine based on a retrospective study. These authors advocated a score using five objective, easily collected variables that are independent in multivariate analysis. The following formula has been proposed: 0.04 x A + 3.1 x B + 1.2 x C + 0.7 x E with A = age, B = common bile duct diameter > 12 mm (no = 0, yes = 1), C = gallstone diameter < 10 mm (no = 0, yes = 1), D = biliary colic (no = 0, yes = 1), and E = cholecystitis (no = 0, yes = 1). According to whether the patient has a score of less or more than 3.5, two groups of patients can be selected with a low (2%) or high risk (32%) of having choledocholithiasis. In an 11 month period, we prospectively compared the pre-operative scores of 175 patients who then underwent traditional cholecystectomy with routine intra-operative cholangiography. We were able to select a low risk group (3.4% if score < 3.5) and a high risk group (25% if score > 3.5) of having choledocholithiasis. Laparoscopic cholecystectomy may be performed without any further investigations in the low risk group (with a risk of 3.5% of choledocholithiasis). In the high risk group, either laparoscopic cholangiography, endoscopic ultrasonography or cholangiography should be entertained.

PO071

### PEPTIC ULCER & GALLSTONES

Ahmed Ibn Idris

Omdurman Military Hospital, Gastroenterology Unit, Sudan

The association of cholelithiasis and peptic ulcer in this study was established by investigating gallbladder abnormalities in 330 patients with endoscopic evidence of duodenal ulcer. Ultrasonography revealed the presence of gallstones in 15 patients (3.3%).

4 patients were diagnosed as having non-functioning gallbladder. 2 of them were subjected to surgery for acute abdomen and both had gallstones. 35 patients out of 50 with gallbladder abnormalities showed a picture of cholecystitis on ultrasonography. Peptic ulcer and diseases of the biliary system are possible in association because of the direct anatomical and physiological neighbourhood and the biochemical interaction between the biliary system, stomach, and duodenum.

PO072

### PRELIMINARY EXPERIENCE OF CHEMICAL ABLATION OF THE GALLBLADDER

X S Zhou, Q Li, S Q Deng

Department of Surgery, Third Teaching Hospital,
Beijing Medical University, Beijing, 100083, China

Chemical ablation of the gallbladder was applied to 6 patients. Because of their severe accompanying diseases, merely cholecystolithotomy and cholecystostomy were performed during emergency operation. These accompanying diseases still constituted risk factors of cholecystectomy after their recovery from acute cholecystitis.

**METHOD** If the biliary tract was free of stone, and 6 or more weeks had passed since cholecystostomy, a microwave antenna for medical use was inserted into the cystic duct under the guidance of the cholecystoscope. The duct wall and the tissue around the orifice were coagulated by microwave heating for 10 seconds at several points and repeated to 4 times at each point. The current used to generate microwave was 50 mA. Within 20 to 36 hours after coagulation, temporary occlusion of the cystic duct was confirmed by cholecystography, and the volume of the gallbladder lumen was measured. The same volume of 95% ethanol alcohol and 5% Tetracycline were injected into the lumen and were kept there for 30 minutes successively. The injection was repeated 4 to 6 hours later. When the drainage was free of bile and less than 10 ml per day, and the cavity was substituted by a narrow fistula on cholecystography, the drainage tube was withdrawn.

**RESULT AND DISCUSSION** In all cases, the fistula closed quickly, and ultrasonography revealed that the gallbladder cavity was obliterated in 4 to 5 weeks after coagulation. One of them had an attack of right subcostal pain. The gallbladder cavity was detected by ultrasonography again in the 9th month after treatment. The occlusion of the cystic duct induced by microwave was temporary, it would be patent again in 3 days after coagulation when the edema subsided. Therefore, we performed chemical ablation within 20 to 36 hours after coagulation, and the volume of sclerosing agent was restricted to the measured gallbladder volume. Following this principle, the leakage of contrast medium into common bile duct and the evidence of bile duct stricture did not occur in any case.

PO073

### SURGICAL TREATMENT OF BILE DUCT STONES

R Madjov, P Chervenkov, N Tzolov

Department of Surgery, Med University, Varna, Bulgaria

The results of diagnosis and treatment of 547 consecutive patients with bile duct stones are analysed. 363 of them - female (mean age - 54.5) and 184 - male (mean age - 62.9). Cholangitis was found in 126 patients and stenosing papillitis in 128.

Most common signs were: pain, heaviness, fever, dyspepsia and jaundice (with mean bilirubin blood level - 94,86 mmol)1). Diagnostic methods of greatest importance were: US, cholangiography, ERCP, PTC and CT. Also intra-operative US and choledochoscopy were quite valuable for the exact diagnosis and treatment of bile duct stones and their complications.

453 of all the patients were operated on. External drainage was performed most commonly after exploration of the bile duct in 282 patients (62,25%) T-tube drainage (48,3%), T-tube + dilation p.Vateri (13,1%), transcystic drainage in 3 patients. Biliodigestive anastomosis was performed in 28,9% (most frequently choledochoduodenostomy - 119 patients). Papillosphincteroplasty - 18 patients (3,97%) and double internal drainage (papillosphincteroplasty + choledochoduodenostomy) in 19 patients (4,19%). Re-explorations were performed in 11 patients (8 for residual stones). Post-operative endoscopic papillotomy and removal of residual stone was performed in 2 patients, and trans T-tube choledochoscopy for missed stone in 2 patients. Post-operative mortality rate - 3,97% (18 patients).

PO074

### INTRAOPERATIVE SPHINCTEROTOMY AND ERCP FOR COMMON BILE DUCT STONES DURING LAPAROSCOPIC CHOLECYSTECTOMY.

M. Gagner, E. Deslandres, A. Pomp, M. Rheault
C. Potvin, R. Leduc
Department of Surgery and Medicine, Hotel-Dieu of Montréal, University of Montreal, Montreal, Quebec, Canada

Laparoscopic methods to retrieve choledocholithiasis (CBD) stones are in their early development. We designed a technique combining intraoperative Endoscopic Retrograde Cholangiopancreatography (ERCP) with laparoscopic cholecystectomy to remove CBD stones.

A pediatric 5 Fr. feeding tube is advanced through the cystic duct until it reaches the duodenum through the papilla, and serves as a leading track for sphincterotomy. The duodenoscope is advanced while the patient is in supine position. Alternatively, the papilla is canulated with the sphincterotome in a standard manner, and sterile methylene blue is injected through the sphincterotome and reflux observed at the cystic duct level.

Of 30 consecutive patients (9 males and 21 females, mean age 58) who had their stones identified by routine intraoperative cholangiography, 28 had their stones extracted after sphincterotomy with a dormia basket or flushed through the cystic duct with saline. Three patients had postoperative asymptomatic transient high serum amylase. One patient underwent laparotomy for an impacted stone in the lower common bile duct. One patient had an esophageal stenosis preventing endoscope insertion and one underwent direct CBD laparoscopic exploration. Two patients had transcystic electrohydraulic lithotripsy for impacted stones. 24 patients had multiple stones and 6 patients had a single stone. Median postopoperative stay was 2 days (range 2-6) and average endoscopic time was 22 minutes (range 15-45).

Intraoperative ERCP does not increase the risk of pancreatitis. It permits clearance of the common duct and prevents to rely on a postoperative ERCP.These results are in favor of intraoperative endoscopic sphincterotomy as an alternative method to extract choledocholithiasis during laparoscopic cholecystectomy.

PO075

### OBSTRUCTIVE JAUNDICE IN MIRIZZI SYNDROME. A NEW SURGICAL TECHNIQUE FOR AVOIDING INJURY TO THE BILE DUCTS

A B Agorogiannis, G Georgiadis and A Dovas

Surgical Unit, District General Hospital of Larisa, Larisa, Greece

The aim of this study is to present our experience upon Mirizzi syndrome and a new technique for the surgical correction of this condition. Mirizzi syndrome has been recognised as obstructive jaundice caused by a large solitary stone impacted in the cystic duct or neck of the gallbladder so as to impinge on the adjacent common hepatic duct.

Nowadays, two variants, and more recently four variants of the syndrome have been described. During the last 15 years at our Institution, we have operated on 3,300 patients with benign diseases of the biliary tract. Among them 726 (22%) had had jaundice, 660 (20%) had acute cholecystitis and 91 (2.78%) suffered with the Mirizzi syndrome. Fifty had type I Mirizzi syndrome and 41 type II. Sixty % of the patients were women and 40% were men. The mean age for the women was 58 years and for men 66 years.

Cholecystectomy on these patients is usually difficult and, because the marked inflammation around Calot's triangle, the dissection is potentially dangerous in this area. To avoid injury of the common bile duct, the Surgeon must be aware of the condition and the diagnosis has to be considered pre-operatively. To minimise dissection around Calot's triangle we dissect the gallbladder from the liver bed, to its neck, then open it by a parallel cholecystotomy, remove the impacted stone and perform cholecystectomy by leaving its neck as a cuff. Then we proceed to suture the wall of the abandoned neck, avoiding potential damage to the common bile duct and the right hepatic artery. In our 91 patients no iatrogenic damage was done pre-operatively and the post-operative mortality was 0%.

We conclude that the essentials to the management of this syndrome are: Pre-operative diagnosis of the condition by ultrasonography and ERCP. Pre-operative classification of the case. In type II syndrome, partial cholecystectomy, removal of the impacted stone and suturing of Hartmann's pouch, protects iatrogenic injury of the right and common hepatic ducts.

PO076

### ACUTE CHOLANGITIS. DEFINITION BY CLINICAL, LABORATORY AND OPERATIVE FINDINGS

A B Agorogiannis and G Georgiadis

Surgical Unit, District General Hospital of Larisa, Larisa, Greece

The aim of this study is to correlate the pre-operative clinical and laboratory findings of acute cholangitis with the operative findings in defining the severity and the prognosis of the disease.

During the last 15 years at our Institution, we operated on 3,300 patients with benign diseases of the biliary tract. Among them 602 patients had acute cholecystitis (18%), and 180 were operated on for acute cholangitis (ACHO) (5.5%). According to their clinical presentation defined by the Charcot's triad (fever, jaundice, pain or chills) or Reynold's fivefold signs (fever, jaundice, pain or chills, mental confusion and phenomena of shock), leucocytes, prothrombin time, lymphocytes and blood urea nitrogen in comparing with the operative findings of bile peritonitis, edema around the extrahepatic bile ducts, edematous wall of the bile ducts, green bile, turbid bile or pus in the main ducts, redness of the mucosa of the common hepatic duct and multiple abscesses of the liver, we calculated these as risk factors. According to the number of risk factors present in every patient with ACHO we divided our patients into 3 groups. Group A with 1-5 risk factors (52 patients). Group B with 5-10 risk factors (59 patients) and Group C with 10-14 risk factors (69 patients). To Group A belong the patients with mild ACHO. To Group B with moderate and to Group C with severe or suppurative ACHO.

We conclude that there was absolute correlation between the presence of clinical signs pre-operatively, the laboratory parameters and the operative findings as to the diagnosis, the severity and the prognosis of acute cholangitis in our cases. By calculating all the above parameters, we feel that we can define ACHO patients into the 3 categories of mild, moderate and severe form of the disease, the 3 types of parameters are absolutely correlated and also define prognostically the morbidity and the mortality of patients with ACHO. The pre-operative parameters as calculated in our cases, compared with the operative findings, are worthy of credit for the diagnosis of the disease. There was an increasing morbidity and mortality rate according to the increasing number of risk factors present in each one group of patients. The mortality in Group A was 0%. In Group B was 0% and in Group C 12%. All the patients who died had present more than 12 risk factors.

PO077

### PARTICULAR ASPECTS OF THE CALCIC BILE SYNDROME

F C Rada, I O Rada, M Mracec, P Cristodor, M Budu
V Santimbreanu, D Ciobanas

Timisoara University of Medicine and Pharmacy,
Str Siret 6, Timisoara 1900, Romania

Calcic bile (CB) syndrome refers to gallbladder mudding with $CaCO_3$. $CaCO_3$ concentrations higher than 50% in bile lead to spontaneous radio-opacity (so that radio-opaque dyes are no longer necessary for preparing the gallbladder for radiology).

We have followed 9 cases with CB, 6 of them having been confirmed by surgery, 1 during autopsy and the other 2 by radiography.

In 6 cases (women, 46-61 years old) we performed chemical analysis of the CB. This revealed the presence of $CaCO_3$ in 41-93% of the cases. In 3 cases, cholesterol calculi were mixed into the CB, 2 cases had radio-opaque bile (containing 83% and 89% $CaCO_3$) and in 1 case the CB was inconclusively radio-opaque (41% $CaCO_3$).

In 2 cases (women, 47, 52 years old) we found CB in the gallbladder basinet, while the gallbladder body was occupied by cholesterinic calculi ($CaCO_3$ 72-93%). 1 case - woman, 76 years old - died of lung cancer; autopsy revealed CB in a crescent form (Rg) at the bottom of the gallbladder and brownish bile at the top. In 1 case (woman, 53 years old) the pasty content of the gallbladder, was drained into the duodenum during surgery for para-esophageal hiatus hernia. In another case (woman, 62 years old) the gallbladder content was spontaneously evacuated into the duodenum, the phenomenon being accompanied by severe biliary colic with subicterus. In the last case (unoperated, man, 78 years old), the radiologic image of the gallbladder varied according to the position of the patient. All our patients had free cystic ducts. Gallbladders showed aspects of chronic inflammation. Echography revealed an echodense structure of the gallbladder with posterior cone of shadow. Spontaneous radio-opacity - without previous administration of radio-opaque dyes - is a sign of diagnostic certainty.

$CaCO_3$ appears to the bile by the combination of $Ca^{2+}$ ions and $HCO_3^-$ ions when pH is 8 or above. Gallbladder evacuation disorders lead to $CaCO_3$ storage, its density being higher than the density of bile.

PO078

### GALLBLADDER LITHIASIS: OPEN VS. LAPAROSCOPIC CHOLECYSTECTOMY. IMPORTANCE OF THE CHOLANGIOGRAPHY

A Principe, E Jovine, ML Lugaresi, M Morganti, A Mazziotti, G Gozzetti

2° Dpt. of Surgery, Univ. of Bologna, S. Orsola Hospital, Bologna, ITALY

Cholecystectomy represents the gold standard in symptomatic gallbladder lithiasis. in the past open cholecistectomy (OC) as achieved an acceptable morbility and mortality rates. Laparoscopic cholecistectomy (LC) has largely placed conventional surgery in the elective treatment of symptomatic cholelithiasis. During OC injuries to the bile ducts and a retained common bile duct stone, occurred an average of 0.5 % in literature. It is not yet possible to know what proportion of these complication will be appeare laparoscopic technique because of early trials. Pre- or operative cholangiography prevent or detect these complications. **PATIENTS AND METHODS** From November 1981 to November 1992, 831 patients underwent cholecystectomy. Have been considered 2 groups: 718 pts. treated with OC (a) and 113 with LC (b). **RESULTS** On group a in 32 pts. operative cholangiography detected choledocal lithiasis but in 118 cases it was not possible to perform any type of this investigation. In the 172 complicated forms occurred 3 jatrogenic lesions (1.7 %) and 5 deaths (2.9 %); in elective surgery 1 residual lithiasis (0.1 %) and 2 deaths (0.3 %). The mean hospital stay in elective and emergency surgery was respectively 7 and 14 days. On group b cholangiography pre- or operative has been always done and the LC, always elected, was useful without main complications or deaths. The mean hospital stay was 2.7 days. **CONCLUSIONS** a) LC is preferable in elective surgery of cholelithiasis. b) pre- or operative cholangiography to prevent residual stones or detect jatrogenic lesions in LC is mandatory and the omission is hazardous because the visual perspective of the operating surgeon as been changed.

PO079 BILE DUCT EXPLORATION FOR CHOLEDOCHOLITHIASIS

E.Yettimis, E.Kalokerinos, P.Vachliotis, E.Kostopanayiotou, H.Tsipras, P.Kapsambelis, Th. Polimeropoulos
Athen's General Hospital
Athen's Greece

This is a retrospective interpretation of our experience in biliary tract surgery in patients with cholelithiasis. During the last 12 years 2344 pts were operated for cholelithiasis and 563 pts of them (24,0%) underwent exploration of the biliary tract (12 pts were explored by choledochoscopy though the dilated cystic duct). 57,5% of the patients were females. The indications for bile duct exploration were: dilatation of the extrahepatic biliary tract (25,4%), multiple small gallstones (24,7%), x-ray diagnosis of choledocholithiasis (20,2%), obstructive jaundice (17,4%), acute gallstone pancreatitis (7,6%), acute cholangitis (3,2%), palpable choledocholithiasis (1,4%). In 288 pts (51,2%) choledocholithiasis accompanied cholelithiasis, in 6,2% of the pts choledocholithiasis wasn't accompanied by gallbladder lithiasis and in 61 pts (10,8%) choledocholithiasis was present in pts subjected to cholecystectomy in the past. In 32% of our pts the bile duct exploration proved negative for stones despite cholelithiasis. A T-tube was placed in 57,2% of the pts and 35,3% underwent a choledochoduodenostomy, 2% had a Roux-en-Y choledochojejunostomy, 2,2% sphincterotomy-sphincteroplasty and 2,3% had primary closure of the common bile duct. In 6 pts the choledochoscopy though the wide cystic duct didn't prove lithiasis and the operation was limited to cholecystectomy. The immediate postoperative complications associated to the operation, were 12,1%. Most important of them were : T-tube related complications (22 pts), retained stones (21 pts), bile leakage (13 pts), postoperative mild pancreatitis (9 pts) and subhepatic collection (3 pts). 10 pts (1,8%) were reoperated for postoperative problems. The total postoperative mortality was 2,3% (13 pts). It is concluded: (a) Bile duct exploration needs special knowledge and attention because of possible complications related to the procedure (b) T-tube intubation is associated with multible problems and whenever is used, care must be undertaken to exclude retained stones with cholangiography and/or choledochoscopy. (c) Choledochopeptic anastomoses is a useful and safe procedure to treat elderly patients with common bile duct stones.

PO080

### TRANSDUODENAL SPHINCTEROPLASTY. IS STILL INDICATED?

Kr Daskalakis, E Mouloudaki, P Vernicos, G Diamantopoulos

3d Surgical Department, Evangelismos Hospital, Athens, Greece

After the wide use of endoscopic sphincterotomy, transduodenal sphincteroplasty became less popular. Despite this, transduodenal sphincteroplasty is a very good procedure to produce free common bile duct drainage, if clear indications are present.
In the 3d Surgical Department of "EVANGELISMOS" Hospital, were performed 31 transduodenal sphincteroplasties in the last 8 years (1985-1992). Eighteen patients were male and 13 female. Their age ranged between 22 to 78 years. In 17 patients of this series transduodenal sphincteroplasty was the initial treatment and in 14 it was reoperation after cholecystectomy with or without common bile duct exploration in the past.
The indications for this procedure were: Common bile duct stones because of hemolytic disease in 2 patients, stenosis of the papilla in 3, acute suppurative cholangitis with impacted stones in 5, obstructive jaundice with impacted stones in 12, residual or recurrent ductal calculi in 7 and rupture of hydatid cysts into the biliary tract in 2.
One patient died in the immediate postoperative period because of acute necrotizing pancreatitis (Mortality: 3,2%). This patient reoperated on, necrosectomy and bursa lavage was performed, but he died in the 20th postoperative day from multiple organ failure.
The immediate postoperative complications were acute necrotizing pancreatitis in one and respiratory infection in another one patient (Morbidity: 6,4%). The mean hospital stay was 9,1 (range: 8-20) days. The long-term results were good.
In conclusion, transduodenal sphincteroplasty is a very good but a little difficult procedure to produce free common bile duct drainage.

PO081 **Sonographic patterns and the chemical composition of gallstones.**

Vela M, Pérez-Ayuso R, Valderrama R, Bianchi L, Bru C, Targarona E, Trías M, Ros E, Terés J.

Gastroenterology, Radiology and Digestive Surgery Departments. Hospital Clínic i Provincial, Barcelona, Spain.

Complete gallstone dissolution after bile acid therapy in eligible patients (radiolucent stones ≤15mm in size in a functioning gallbladder) takes place in about 1/3 of cases, indicating that such stones are composed of pure cholesterol. A relationship between sonographic gallstone patterns and chemical stone composition has been described. Therefore, it is interesting to learn whether sonographic stone images can identify pure cholesterol gallstones and improve the efficacy of oral bile acid treatment.
**Aim**. To investigate the relationship between the sonographic pattern and the chemical composition of gallstones.
**Patients and methods**. 35 patients with symptomatic gallstones who were eligible for oral bile acid therapy and underwent cholecistectomy were studied. An abdominal sonographic examination was performed before surgery, and at the operation the stones were retrieved and their chemical composition was analized by infrared spectroscopy. Patients were divided into 3 groups: I, 15 with pure cholesterol stones (>90% cholesterol). II, 10 with pigment stones (<20% cholesterol). III, 10 with mixed cholesterol stones (>10% pigment or calcium carbonate). Sonograms were independently reported by 2 radiologists who were unaware of the chemical composition of the stones. The images were assigned to one of two patterns: 1) One or more hyperechogenic images with distal acoustic shadow. 2) One or more hiperechogenic images without distal acoustic shadow.
**Results**.

| | Sonographic pattern | |
|---|---|---|
| **Chemical composition** | **Shadow** | **No shadow** |
| Pure cholesterol | 15 | 0 |
| Pigment | 7 | 3 |
| Mixed cholesterol | 9 | 1 |

100% of pure cholesterol stones and 70% of pigment stones disclosed an acoustic shadow. 48% of the stones with acoustic shadowing were made up of pure cholesterol.
**Conclusion**. Because 3/10 pigment stones and 1/10 mixed cholesterol stones ≤15 mm do not disclose an acoustic shadow at sonographic examination, and it is known that approximately 60% and 40% of the stones who fail to dissolve with bile acids are pigment and mixed cholesterol stones, respectively, we suggest that the inclusion of the presence of a clear acoustic shadow in the gallbladder sonogram as a selection criterion for bile acid therapy could improve its efficacy by about 15%.

PO082 EMPHYSEMATOUS CHOLECYSTITIS.

C.Fotiadis, M.Safioleas, K.Kontzoglou,
S.Rossonis, J.Delladetsima, Gr.Skalkeas.

2nd Department of Propedeutic Surgery-University of Athens, LAIKON Hospital, GREECE.

A variant of acute cholecystitis is emphysematous cholecystitis. Though 120 cases only have been reported in the literature the surgeons have to think about this rare entity because of its worse prognosis than that of acute cholecystitis with a mortality rate 15% (in acute cholecystitis 4,1%) and its higher frequency of perforation of the gallbladder (4 times more often) than that of acute cholecystitis. During the last 10 year-period, three cases of emphysematous cholecystitis were treated in 2nd Department of Propedeutic Surgery of University of Athens. The age of the patients ranged from 55 to 67 years and there were two men and one woman. The general condition of the patients at their admittance was severely affected, presenting acute pain in the right subcostal region (space), vomiting and fever ranged from 38,2$^{o}$C to 39,5$^{o}$C with main laboratory finding the leucocytosis. All of the three patients had known cholelithiasis and two of them diabetes mellitus. The diagnosis was established upon the plain roentgenogram of the abdomen and the ultrasonography and in one case the CT scan of the abdomen. One of the patients was operated urgently because of his bad and aggravated general condition and the rest two patients in 48hrs period after the admission. All of the patients underwent cholecystectomy. The cultures of the gall revealed. E.coli and Cl Welchi in two cases. The postoperative recovery was uncomplicated and the patients left the hospital doing well. Conclusively, emphysematous cholecystitis requires timely diagnosis and surgical treatment promptly under antibiotic cover, in order to reduce morbitity and mortality rates.

PO083

### PRIMARY CARCINOMA OF THE GALLBLADDER REVIEW OF 143 CASES

JP Arnaud, C Casa, JCl Schall, JP Jacob
J Ronceray

C.H.U.ANGERS - Department of Visceral Surgery

4, rue Larrey 49033 ANGERX CEDEX 01 - FRANCE

The record of 143 patients with gallbladder carcinoma operatively treated between 1973 and 1990 were retrospectively reviewed. There were 115 women and 28 men, with an average age of 69.3 years (range 36-89). Gallstones were described in 73.4% of the patients. The symptoms of primary carcinoma of the gallbladder were similar to those of other forms of biliary tract disease. Abdominal pain was the most common symptom and was present in 103 of our 143 patients (72%).Jaundice was present in 83 patients (58%) and weight loss in 68 (47.5%). The pre-operative diagnosis was made in only 28.7 per cent of the cases. The duration of symptoms in our patients varied between 3 days and 17 months. Surgical procedures included cholecystectomy alone (24 patients), cholecystectomy and resection of the hepatic bed (17 patients), and exploration with biopsy or bypass (20 patients). Only 21.5% of the patients inderwent curative surgery.

In 95.8% of the carcinomas of the gallbladder, the histologic type was adenocarcinoma. Intramucosal carcinoma (T1) was proved in four patients, and, in eight patients, the tumors has invaded the muscle coat (T2). 29 patients presented a carcinoma extending to the subserosal tissue of the gallbladder wall (T3); 53 patients had involvement of adjacent organs (T4) and 49 developed metastases (T5).

Overall five year survival rate was 11%. For patients whose tumor was limited to the gallbladder wall (T1,T2,T3), the acturial 5-year survival rate was respectively 100%, 29% and 23%.For patients with T4 and T5 tumor, the 5 year survival rate was nil. The prognosis mainly depended on the tumoral extension in depth. For the early stages, the survival rate could be higher with a more aggressive surgical procedure. For the usually observed tumors which extend beyond the gallbladder, the prognosis has not been improved.

PO084

### CORRELATION BETWEEN PATTERN OF GALLBLADDER MUCOPROTEINS AND LITHIASIS CRISTALLOGRAPHY COMPOSITION

J Alvarez, R Lobato, Maillo, J Fradejas, P Artuñedo, Arizaga, M Moreno Azcoita

Hospital Universitario de Getafe, Madrid, Spain

Mucoproteins are the basement where different compounds of the bile are consolidated to form a macroscopic lithiasis. There are three patterns of mucoprotein and three cristallography compounds of the lithiasis.

OBJECTIVE: Assess if every pattern of gallbladder mucoprotein is correlated with different substances appointment (sic), to form different lithiasis.

PATIENTS AND METHODS: A prospective study in 100 patients operated on for cholelithiasis. Different histochemical methods with colour techniques were used to identify the pattern of mucoproteins. Every lithiasis was studied by means of cristallography analysis of its main compounds: cholesterol, bilirubin, calcium carbonate and proteins.

| | | |
|---|---|---|
| RESULTS: | Epithelial MP: | 1-9 (3.77 +/- 1.84) |
| | Gland MP: | 0-9 (4.77 +/- 2.18) |
| | Cholesterol: | 7-92 (54.6+/- 19.77) |
| | Bilirubin: | 5-90 (41.72 +/- 18.88) |
| | Carbonate: | 0-5 (1.11 +/- 1.61) |

CONCLUSION: In our experience an unspecific secretion of mucoprotein favours consolidating a lithiasis, and before this circumstance different compounds are aggregated.

PO085 SUBTOTAL CHOLECYSTECTOMY

C Katsohis, J Prousalidis, A Michalopoulos, E Tzartinoglou, H Aletras
1st Propedeutic Surgical Clinic,
Aristotelian University of Thessaloniki

Subtotal cholecystectomy was carried out on 32 patients from 1972 to 1991, whereas at the same period of time 1620 total cholecystectomies were performed. The indications for subtotal cholecystectomy were severe inflammation and/or severe fibrosis in 29 patients, and Mirizzi's syndrome in 3 patients. The morbidity was insignificant, but two patients, one from severe sepsis and the other from cancer, that co-existed with cholelithiasis, died. In follow up studies ranging for 1 to 9 years, there was one patient with remaining stones in the common bile duct and no patients with post cholecystectomy sequelae. Subtotal cholecystectomy is a safe and definitive operation in patients for whom the standard procedure could be dangerous. This operation is less burdensome and has fewer complications than cholecystectomy.

PO086 ULTRASONOGRAPHY OF ACUTE ACALCULOUS CHOLECYSTITIS

C Schulze[1], E Kohlberger[2], N Freudenberg[3]

Departments of Radiology[1], Surgery[2], and Pathology[3], University of Freiburg, Freiburg, Germany

Predisposing factors for acute acalculous cholecystitis (AAC) are common in critically injured patients. In a study conducted from 1987 to October 1992 AAC was found using sonography in 19 patients, most of whom were receiving ventilatory support and parenteral nutrition. The patients were examined supine and in the left lateral decubitus position with a 3.5 MHz transducer. Cholecystectomy was performed in all patients , followed by histological examination. Sonography identified AAC when one of the following criteria applied: pericholecystic fluid or subserosal oedema without ascites, intramural gas, or sloughed mucosal membrane. Sludge, gallbladder distension or gallbladder wall thickness > 4 mm only meant no immediate indication for cholecystectomy. Sonography was performed again on these patients within the next 24 h. All 19 patients managed with cholecystectomy had pathologic evidence of acute inflammation. Areas of gangrene were present in 6 cases. In 3 patients stones of up to 5 mm diameter were found in the gallbladder intraoperatively. All patients survived the operation.

The challenge in securing a preoperative diagnosis arises from the presence of other organ system injuries and the need for analgesia and sedation. Fever and leucocytosis, while commonly present, were too nonspecific to be of any great diagnostic benefit. Abdominal tenderness and liver function abnormalities were frequently absent. We conclude that ultrasound is an important modality at the patient's bedside in the prompt diagnosis and treatment of AAC.

PO087

### URGENT SURGERY IN ACUTE CHOLECYSTITIS

F. Seferiadis, K. Kindylidis, A. Mystakidis,
G. Perpyracis, D. Bastas, V. Mouzakis,
G. Velmachos
"Sotiria" General Hospital, Surgical Unit,
Athens, Greece

The management of any patient with the diagnosis of an acute biliary condition, must be argued for the individual case, guided by certain general indications. Indications for surgery during an acute attack of cholecystitis are: a sustained fever beyond 48 hours, signs of peritoneal irritation, features of cholangitis, general peritonitis, presence of heart disease.

520 cholecystectomies were performed over a 6 year period in our surgical unit of which 52 were early cholecystectomies for acute cholecystitis, cholecystostomies were carried out in 4,2%. The overall hospital stay was 16,1 days. Ultrasonography was carried out in all cases. Empyema and gangrene were found in 36,1% and calculi of the common bile duct in 16,2% of the cases.

The mortality rate for early cholecystectomy was 0,4%. There were no significant differences in postoperative complications between early and ellective surgery. But if the structures cannot be recognized because of the presence of oedema it is safer to abandon cholecystectomy in favour of cholecystostomy.

Cholecystectomy is clearly ideal provided it is safe. Experts in gallbladder surgery regard cholecystostomy with condescension because they always find it possible to remove the gallbladder during an acute attack. Thus no shame should be experienced in occasionally carrying out cholecystostomy in an emergency.

PO088

### PERI-AMPULLARY DUODENAL DIVERTICULA: ASSOCIATION WITH BILIARY TRACT AND PANCREATIC DISEASES

M Rubinic, N Ivaniš, A Depolo, M Uravić, D Štimac

University Internal and Surgical Clinic, Rijeka
Croatia

Peri-ampullary duodenal diverticula are known to be associated with an increased incidence of biliary tract and pancreatic diseases. The nature of the association remains uncertain. An analysis of 1336 ERCP had been carried out within a period of five years. The result was 89 (7,5%) duodenal diverticula, found in association with papilla Vateri. Of these 40 were in male patients of an average age of 65, and 49 in women averaging 70 years of age. The diverticula were divided into six types, so that the possibility of cannulation of the papilla opening existed in 62 (70%) of the patients.

The most frequent indication showing the need for examination was the obstruction of gallbladder drainage in 35, obstructive icterus in 13, and post-cholecystectomy stage in 5 patients, and chronic pancreatitis in 10 patients. In 52% of the patients chole or choledocholithiasis was found, in 16% chronic pancreatitis, and in 13% stenosis of the distal part of the choledochus. In 3% of the patients a tumour in association with diverticulum was found, and in 16% of the patients the findings were normal. On the basis of the results the conclusion is drawn that there is close association between biliary and pancreatic diseases and duodenal diverticula associated with papilla Vateri.

PO089

### MODE OF RECURRENCE AFTER RADICAL SURGERY FOR CARCINOMA OF THE GALLBLADDER

K Yoshida, Y Shirai, K Tsukada, K Uchida, I Kurosaki, C Kawai
Department of Surgery, The Nippon Dental University School of Dentistry at Niigata, Niigata, Japan

Purpose: To investigate more rational surgical approach for gallbladder cancer (GBC), mode of recurrence was studied based on the local pathological condition.

Patients: Prognosis and site of recurrence were studied in 92 patients who underwent radical surgery for GBC since 1982. Procedures employed included cholecystectomy (C) ± bile duct resection (BD) in 16 cases, C + wedge resection of the liver (L) in 10, C + L + BD in 39, pancreatoduodenectomy (PD) ± L in 18, extended hepatic lobectomy (EHR) ± BD in 6 and EHR + PD in 2. Lymph node dissection was done in all.
Results: Excluding one operative death, cumulative 5 - year survival rates in pT1 (16 cases), pT2 (38), pT3 (23), and pT4 (13) categories were 100%, 75.8%, 41.8% and 0% respectively. 33 sites of recurrence were identified in 26 patients. Most frequent site was hepatic hilum (11 cases) followed by peritoneum (8), liver (8), lung or bone (5) and para-aortic node (1). Most important contributing factor to hilar recurrence was cancer positive margin in cases with direct hilar invasion. In patients with definite hilar involvement, negative margin was obtained in only 2 of 16 cases even by exteded surgery. Mode of recurrence after curative resection was mainly hematogenous.

Conclusion: Even though results of curative resection of GBC is satisfactory, hilar invasion is still the barrier for surgical cure. For early detection before jaundice, routine use of ultrasonography for abdominal symptoms and macroscopic examination of the gallbladder removed for presumed benign diseases were quite useful.

PO090 SAFETY OF BILIARY PROCEDURES PERFORMED BY RESIDENTS

M A Delgado, F Canillas, M Martin, A Carabias, J M Jover, P Artuñedo, L M Diaz Jimenez, M Moreno

H. Universitario de Getafe, Ctra. Toledo, 12.5 Km, Madrid, Spain

The safety of surgical procedures performed by residents in teaching hospitals has been the subject of extensive public and medic discussions.

**Material and Methods:**

We retrospectively studied the records of consecutive patients undergoing surgical treatment for biliary diseases during 1992 (November 91 - November 92)

We analyzed diagnosis, attending surgeon, surgical time, blood units needed, urgent or not, hospital stay, type of surgery performed and complications.

**RESULTS:**

Residents performed 86(46.2%) of biliary procedures (staff 100(53.7%)), in patients with similar risks (ASAI 39.55 (St 38%); ASA II (52.3%) and ASA III 8.13% (St 9%); operative procedures done were simple cholecystectomy 80% (St 66%); laparoscopic surgery 1.16% (10%); sphincteroplasty 2.3% (85), choledochoduodenostomy 5.8% (6%); choledochotomy plus T-tube 3.4% (4%) and hepaticojejnostomy)% (2%).

Surgical time found was of 184.46 mts (191.46), length hospital stay 6.54 days (Staff 7.85) and complications 11.6% (10%):8.13% infectious (6%); residual stone 1.16% (1%); respiratory problems 1.16% (1%); laparotomic hernia 1.16% (1%) and paralytic ileo 1.16% (1%). For cholelithiasis, residents performed 35 operations (st 29), with a post-operative stay of 5.34(3.96), mean surgical time of 150.2 mt (137.4), similar surgical risks, 94.2% of cholecystectomies (72.4%), 5.9% sphincteroplasties (6.8%) and 11.4% complications (6.8%). For cholecystitis residents performed 47 procedures (65), with a medium hospital stay of 5.78 (7.43), 68.4% operated within 24 hours (56.9%) medium surgical time 163.2 mts. (155.33), similar surgical risks, 87.2% simple cholecystectomies (69.22%), 2.12% laparoscopic procedures (9.2%), 4.25% c-duodenostomy (7.25%), 6.38% choledochotomy (4.6%,) 0% sphincterotomy (6.11%) and 12.7% complications (9.2%).

**CONCLUSIONS:**

Biliary procedures are safely performed by residents, with similar surgical time, length of hospital stay, surgical procedures and complications. We can stand out (sic) less laparoscopy procedures performed by residents.

PO091 **ROUX-EN-Y HEPATICOJEJUNOSTOMY FOR BILIARY ACCESS AND PERCUTANEOUS TRANSJEJUNAL INTRAHEPATIC STRICTURE DILATATION AND STONE EXTRACTION.**

**JEJ Krige, SJ Beningfield*, PC Bornman, J Terblanche.**
**Departments of Surgery and Radiology*, University of Cape Town and MRC Liver Research Centre, Cape Town, South Africa.**

The management of benign intrahepatic strictures and stones, especially in patients with bilateral disease, is complex with a high incidence of recurrence. The efficacy of operative stricture dilatation, stone extraction and creation of a Roux-en-Y hepaticojejunostomy with an afferent access loop for subsequent radiological percutaneous transjejunal biliary intervention, was evaluated.

Twenty two symptomatic adult patients (14 women; 8 men : mean age 31 years; range 22-64 years) with benign intrahepatic strictures and stones underwent 72 postoperative percutaneous transjejunal biliary balloon dilatations between 1986 and 1992. The intrahepatic strictures and stones were secondary to iatrogenic hepatic duct injury (n=5) Caroli's disease (n=2) choledocal cyst (n=2) and primary intrahepatic stones (n=13). All patients underwent intraoperative stricture dilatation, stone extraction and construction of a Roux-en-Y side-to-side hepaticojejunostomy with a 12 cm jejunal access loop marked with silver clips and attached to the anterior abdominal wall. Subsequent postoperative percutaneous transjejunal biliary dilatation of residual or recurrent intrahepatic strictures and stone extraction was performed using a biliary guide-wire, co-axial catheter and 7 Fr Gruntzig angioplasty balloon catheter. Three patients with intrahepatic stones had in addition resection of the left lateral segment during the primary procedure.

Five patients with iatrogenic strictures underwent 22 dilatations (mean 3, range 1-6) and are asymptomatic without residual stones. Seventeen patients who had Caroli's, choledocal cysts or primary intrahepatic stones underwent 50 dilatations (mean 2.9, range 1-9). Two patients have residual intrahepatic stones and one has required a left lateral segmentectomy for removal of stones. Minor local or ductal complications related to percutaneous dilatation and stone extraction occurred in three patients without long-term sequelae.

The combined radiological and surgical approach using a Roux-en-Y hepaticojejunostomy and biliary access loop with post-operative percutaneous transjejunal biliary dilatation and stone extraction provides an effective method of treating symptomatic patients with complex residual or recurrent intrahepatic strictures and stones.

PO092 **BILIARY TRACT CARCINOMA: OUR EXPERIENCE**

G V Velmachos, F Seferiadis, D Bastas, V Mouzakis,
G Garinis, N Lambrinaki, N Kitromilis

Surgical Unit, Sotiria Gen Hospital of Athens,
Greece

Biliary tract carcinoma (BTC) is generally associated with a poor prognosis, because most patients present advanced disease, although they are diagnosed with increasing convenience and frequency. The aim of this study was to evaluate retrospectively the clinical course of our patients with BTC.

During the period 1987-90 37 patients (27 males; 10 females) affected with BTC were treated surgically at the Gen. Surg. Department of Sotiria Hospital of Athens. The mean age was 67 years (Range 45-79). The localisation of the carcinoma was in 19 (51.3%) patients the gallbladder, in 5 (13.6%) patients the hepatic hilum, in 3 (8.1%) patients the common bile duct, in 3 (8.1%) patients the choledochus and in 7 (18.9%) patients the papilla. Obstructive jaundice was the revealing symptom (72%), palpable mass (37%), pain (42%), nausea and vomiting (29%), weight loss (26%). Ultrasonography, ERCP and CT were of particular value in pre-operative diagnosis.

A surgical procedure was possible in 35 of the cases. Cholecystectomies for the gallbladder carcinoma were performed in 18 patients (two associated with wedge hepatic resection), hepatobiliary resections were performed for the tumours of the hilum in 5 patients, biliary resections were performed for tumours localised in the choledochus or the common bile duct in 4 patients, duodenocephalopancreatectomy in 2 patients for carcinoma of the papilla, side to side choledochoduodenostomy in 4 elderly patients for carcinoma of the papilla and two tumours of the common bile duct. No surgical treatment was performed in two elderly patients with severe diabetes and multiple metastases. Complications appeared in 8 patients. The post-operative mortality was 9.2%. Long-term survival was good for the gallbladder carcinoma (<2.5 years), 6-18 months for the hilar carcinoma, 18-28 months for the common bile duct and 10-22 months for the papilloma tumours.

In conclusion, the results of our study seem to show that a curative resection is rarely possible for treatment of biliary tract carcinoma, although the palliative techniques are able to improve the extension of survival and the quality of life of these patients.

PO093

### MIRIZZI SYNDROME : SURGICAL AND CLINICAL CONSIDERATIONS

M Morosini, M Granello, F Donadio

I° Department of Surgery - Martini Hospital, Turin, Italy

The so-called Mirizzi syndrome is a rare condition in the heterogeneous field of benign biliary diseases that can complicate the natural history of gallstones. In this study we report our experience in the surgical treatment of type 1 Mirizzi syndrome.

Patients and method: Between 1982 and 1991 in our department we operated on 2420 patients for benign biliary tract diseases. Among these we have encountered 19 cases of the Mirizzi syndrome, representing a prevalence of 0,78%. The mean age was 48 years - range 24-62 nearly all of them (79%) females. The most common signs and symptoms were pain (100%), fever (89%), jaundice (74%) and pruritus (63%). The diagnosis was made pre-operatively by intravenous cholangiography in 2 cases (10,5%), by ERCP in 8 (42%), by ultrasonography in 7 (37%) and in 2 the syndrome was discovered intra-operatively.

All patients underwent cholecystectomy and bile duct exploration; in 13 cases (68%) a T-tube was placed, in 4 (21%) a Roux-en-Y biliojejunostomy was performed and 2 patients required the repair of superficial erosion of the hepatic duct wall. There was no operative mortality. The morbidity was 10,5% : 1 subphrenic abscess and 1 biliary leakage.

Discussion: There are nowadays two surgical and cholangiographic variants of the syndrome: **type 1** obstruction of the common hepatic duct caused by a solitary stone impacted in the cystic duct or in Hartmann's pouch of the gallbladder and **type 2** cholecystocholedochol fistula due to a calculus that has eroded partly or completely into the common bile duct. As recently suggested by Csendes and others (1989), we believe that the original Mirizzi syndrome is only the first but probably the most important step of a complicated process that can lead to the development of a true cholecystobiliary fistula. So the diagnosis at this first stage must be very careful in order to prevent the progression of the inflammatory disease. In every way the Mirizzi syndrome still remains a difficult challenge for biliary surgeons.

PO094 **ISCHEMIC STRICTURE OF THE COMMON BILE DUCT AFTER CHOLECYSTECTOMY**

M.Sianesi , M.Zannoni , E.Zanella

Surgical Pathology,University of Parma,
Parma,Italy

The appearance of extra-hepatic obstructive jaundice in patients who undergone cholecystectomy represents a complex pathogenetic problem;the causes of obstruction beeing almost exclusively represented by residual or recurrent stones,papillitis,and iatrogenic injuries overlooked during surgery.
Apart from these conditions,generally the appearance of obstructive jaundice due to incomplete stricture of the common bile duct,in absence of liver and/or bile duct lesions present at surgery,has been repeatedly reported.
Ischemic pathogenesis of late strictures of the common bile duct following cholecystectomy have been for a long time hypotized and anatomo-surgical studies on blood supply to the extrahepatic bile ducts have undubtedly contributed to support this point of view.
Two clinical cases,in which ischemic pathogenesis of late (11 months and 5 years after surgery respectively) bile duct stricture could be suggested,are reported.
Beside an anatomo-surgical appraisal on blood supply to the common bile duct,clinical features of these lesions and their menagement are discussed,pointing out that bilio-digestive anastomosis results as the procedure of choice.

PO095

### INTRAHEPATIC ANASTOMOSES FOR MALIGNANT AND BENIGN BILIARY OBSTRUCTION

**B Launois, J M Catheline, S Landen, G J Maddern**

**Department of Digestive Surgery and transplantation Unit, Pontchaillou Hospital, Rennes, France**

The use of intrahepatic biliary anastomoses is sometimes necessary in patients undergoing resective or palliative surgery at the hepatic hilus. Over a 23 year period we have performed intrahepatic bilio-enteric anastomoses in 54 patients (15 palliative, 35 curative, 4 benign) with a median age of 55 years (range 15-82). There was a hospital mortality of 4 patients in both the "palliative" and "curative" group. A significant fall in both the serum bilirubin and alcaline phosphatase occurred after surgery ($p < 0.05$). Long term follow up was possible in 42 patients.

Recurrent stenosis due to recurrent disease occurred in 89 % of the palliative group and 69 % of the "curative" group. There were 2 stenosis in 4 patients with benign disease. Thirty-five patients developed recurrent cholangitis, 5 without apparent stenosis.

The median survival for the palliative group was 4.5 months (range 2 days-32 months) and in the curative group it was 21.7 months (range 0.5-148 months) while all patients in the benign group remain alive.

Despite the advent of modern endoscopic and percutaneous intubation techniques, intra-hepatic anastomoses after tumour resection offer the only chance of cure for obstructing hilar malignancy.

PO096 MIRIZZI SYNDROME - THE DIAGNOSIS AND THERAPY

M Ryska, J Skala, F Belina, J Dosedel

Surgery and Gastroenterology dept.,
Charles University, Prague.

In 1948 there was described uncommon cause of chole - stasis by Pablo L.Mirizzi: extraductal partial com - pression of common hepatic duct. Clinical signs are expressed by jaundice and recurrent cholangoitis.
4 types of Mirizzi syndrome are distinguished in li - terature. During last 4 years we treated 7 patients with Mirizzi syndrome. All of them had Mirizzi syndro- me diagnosed before operation.
There is very important for surgeon to determine the place of the bile duct obstruction and its nature before operation. Ultrasonography shows usually di - latation of intrahepatic bile ducts and normal cho - ledochus. ERC alone or with PTC are able to inform us exactly about stenosis as well as the character of it and about the present of cholecystobiliaric fistula.

PO097

### CARCINOMA OF THE PROXIMAL BILE DUCT (KLATSKIN) - ANALYSIS OF OUR 5-YEAR SERIES - 34 PATIENTS

S Repše, E Gadžijev, M Čalič, F Jelenc, B Štabuc

Department of Gastroenterologic Surgery, Medical Center, Zaloška 7, 61000 Ljubljana, Slovenia

The incidence of proximal bile duct carcinoma in our country is increasing. During the last decade our approach to the treatment of these patients has changed and aggresive surgical treatment has been accepted. Sceletonization-resection is justified as long as it is technically feasible and the patients do not present any signs of tumor dissemination.

During the 5-year period from Jan.1st 1987 to Dec.31st 1991, 34 patients with carcinoma of the proximal bile duct were treated at our Department (13 males, 21 females). Supposed radical or only palliative resection was possible in 27 patients (resectability rate 78%). Thus presumably radical procedure was performed in 17 patients, and palliative in 10 patients. In 7 patients resection was not possible any more: two of them had biliodigestive by-pass performed and in another two intraoperative endoprosthesis was inserted, while only exploration and biopsy were done in the remaining three patients.

We had no intraoperative deaths but postoperatively death occured in four patients (mortality rate 11,7%): two patients died after palliative resection, one after intraoperative endoprosthesis insertion and one after exploration. Mortality rate for 27 resected patients was 7,4% (2 patients).

Among the 27 patients with resection of Klatskin tumor 7 had type I, 8 type II, 3 type IIIa and 9 patients had type IIIb hilar carcinoma of extrahepatic bile duct.

In December 1992 7 of the 27 resected patients are still alive 18 to 30 months after surgery. Mean survival time for all patients after apparently radical surgery (17 pts) was 12 months and for patients after paliative

PO098 **ROLE OF LEFT HEPATICOJEJUNOSTOMY IN MALIGNANT HILAR BILIARY OBSTRUCTION**

T.K. Malik, S.Madaan, R. Malik

Deptt. of Surgery, Maulana Azad Medical College, New Delhi

Analysis of the palliative biliary - enteric bypass in 34 patients with unresectable malignant obstruction at the confluence of hepatic ducts was carried out. There were 16 men and 18 women, aged 31 - 73 years. The site of obstruction was shown on percutaneous transhepatic cholangiography or endoscopic retrograde cholangiography. Left hepatic duct was used for anastomosis by lowering the left hepatic ductal system from the undersurface of the quadrate lobe in 10 patients and the round ligament approach being used in 21 patients. In three patients, hepaticojejunostomy by the technique of longmire and sandford was preferred. Overall hospital mortality was 21% and the mean survival was 10 months. Although tumor removal with adequate biliary-enteric repair is preferred treatment, but in patients with advanced disease, palliative biliary-enteric bypass is a suitable procedure becuase besides providing the patient a better quality of life free of jaundice and recurrent cholangitis it also avoids the problems of indwelling tubes which may become blocked and require replacement.

PO099

### SURGICAL TREATMENT OF 431 CASES WITH ACUTE OBSTRUCTIVE SUPPURATIVE CHOLANGITIS

Yang Sen-hua, Yang Yi-cun, Sun Bian-sheng

Department of Surgery, Chengdu Third People's Hospital, Chengdu, Sichuan, China

From 1977 to 1989 the authors have treated 431 cases with acute obstructive suppurative cholangitis (AOSC), before 1982 71/169 cases (42.01%) died and after 1983 33/262 (12.59%) ($P<0.05$). Why did the mortality reduce significantly in the later stage? This is because since 1983 the authors emphasised: (1) Earlier operation. The mortality of AOSC increases as clinical features such as pain, jaundice, fever and shaking chills become heavier, but the manifestations of hepatocholangitis (121/431 cases - 28.07%) might be lacking in jaundice even with more serious lesions. Improper delayed operation would be associated with a very high mortality. (2) BUS monitoring peri-operatively to detect intrahepatic lesions including duct stone, ascaris and dilatation, liver and subphrenic abscesses, etc. (3) Simpler operation for decompression and drainage of the obstructed duct and infected bile is the first choice. But important co-existing lesions as before-mentioned should not be overlooked. 92 cases had their liver abscess (24), subphrenic abscess (27), abdominal abscess (33) and acute pancreatitis (8) managed. (4) Anaerobic infection occurred more frequently in bile duct stone, obstruction and infection and in elderly patients (137/431 cases - 31.79% were $\geqslant$ 60). Intravenous drip and peritoneal irrigation with Metronidazole and other broad-spectrum antibiotics were routinely given in these cases. (5) Post-operative complications comprising sepsis, shock and MOF, often the lethal causes, should be prevented and treated intensively. Supportive treatment is of course, fundamentally important.

PO100 **ACUTE CHOLECYSTITIS AS A FIRST MANIFESTATION OF AN UNSUSPECTED GALLBLADDER CARCINOMA. A LAPAROSCOPICAL DILEMMA.**

E Mª Targarona, MJ Pons, P Viella, M Trías
Service of Surgery, Hospital Clinic, University of Barcelona. Barcelona, Spain.

Laparoscopic cholecystectomy has emerged as the treatment of choice for symptomatic cholelithiasis, and the number of contraindications has sharply decreased in the last months. Nevertheless, gallbladder carcinoma (GC) remains as a formal contraindication for LC. The incidence of gallbladder carcinoma in patients operated for cholelithiasis is 1,5-2%, and 80% of early GC are incidental findings during cholecystectomy. Diagnostic of GC during LC may be impaired by decreased tactil feeling. In recent years , has been described 5 cases of incidental GC found during LC, and all patients developed parietal seeding few months after surgery. We present a case of GC that not was recognised during LC. **CASE REPORT:** A 67 y. female diagnosed 25 years before of colelithiasis, presented with an history of biliary colic pain. 2 ultrasonographic explorations performed in the last 5 months revealed the existence of 2 stones of 2 cm. with a normal gallbladder wall. The bile duct was dilated ( 10 mm). Blood hepatic test were normal. During LC, a distended and immflammatory gallbladder was found, and the diagnosis of acute cholecystitis was stablished. An intraoperative colangiography was normal. The patient was discharged at fourth day without incidences. The pathological study reported a moderatelly differentiated adenocarcinoma afecting the whole wall and perineural invasion. 5 months after, the patient developed two lumps in the umbilical and right subcostal scar, and the biopsy was compatible with adenocarcinoma deposits. **CONCLUSION:** The incidence of unsuspected GC during LC is low, but the misdiagnosis of this tumour may preclude a correct treatment or may induce the dissemination of the tumours trough the trocars tracks. Laparoscopic surgeons must to keep this possibility in mind, and if GC is suspected during LC, a frozen pathological study should be performed and the cholecystectomy converted to an open procedure.

PO101

### PLASTIC OR SELF-EXPANDING METAL STENTS FOR PALLIATION OF MALIGNANT BILIARY OBSTRUCTION ?

A Polydorou, S Rizos, A Vezakis, C Potaris, P Kondylis, B Golematis.

Dept of Surgery, Hippocration Hospital Athens, GREECE.

Endoscopic biliary drainage with endoprostheses (EP) has become a well-established palliative treatment for jaundiced patients with unresectable tumors. The main late complication is blockage of the EP by biliary sludge, occuring in 30-40% of patients. This problem remains still unsolved. The development of self-expanding metal stents allowed the insertion of large diameter EP in order to eliminate the blockage of the EP. The aim of this study is to compare small diameter plastic EP with large diameter metal EP. During the period 1990-1992, 78 patients (M/F 52/26) with mean age 66 years (range 42-91) and malignant biliary stricture were referred for endoscopic drainage. Forty eight (62%) pts received a 10 Fr Amsterdam type EP (group A) and 30 (38%) metal EP Wallstent with 10 mm maximum diameter (group B). Patients with low CBD or type I (Bismuth) strictures were included in the study. Successful EP insertion was similar in both groups [A=48 (100%), B=30 (100%)], early procedure related complications were seen in 3 (6%) (A) and 2 (7%) (B) ($p>0.05$), late EP blockage occured in 23 (48%) (A) and 7 (23%) (B) ($p<0.05$). EP blockage in group A was treated with EP change. Wallstent blockage was due to tumor overgrowth in 3 pts and tumor ingrowth in 4 pts. This complication was treated with insertion of a plastic EP through the Wallstent (5 pts) and 2nd Wallstent placement (2 pts). Mean survival for dead patients in group A=38/48 was 26 weeks and in group B=24 was 28 weeks ($p>0.05$). Cost of the initial treatment was 10 times higher in the group B due to high price of the metal EP.
The occlusion rate by biliary sludge was significantly less in group B pts and therefore repetitive EP changes can be prevented by the use of the metal EP. There was a number of EP blockage in group B due to tumor overgrowth or ingrowth. The high cost of the metal EP is a disadvantage. Randomized trials are required to evaluate the cost benefit ratio for the use of the metal EP instead of the plastic EP.

PO102

### OBSTRUCTIVE JAUNDICE: WALLSTEN METALLIC ENDOPROSTHESIS

I Pinto, A Carabias, R Tobio, M A Delgado, J L Ramos, J M Jover, F Lobato, D Jimenez, M Moreno

H. Universitario de Getafe, Madrid, Spain

### INTRODUCTION

Permanent prosthesis in bile ducts is a good palliation for malignant biliary non operative obstruction.

### MATERIAL AND METHODS

Between April '90 and August '91, 25 biliary prostheses of Wallsten were implanted in 20 patients with biliary obstruction: 11 men and 9 women. Age distribution ranged 33-84 years old (68.2). Nineteen were malignant, 10 cholangiocarcinoma, 5 pancreatic cancer and 4 nodes from metastases in the area of ampulla of Vater. One was benign in a patient with two stenoses of biliodigestive bypass. Early complications (<72 hours) in 15% cases: biliary sepsis, pancreatitis and subcapsular haematoma.

Late complications found: cholangitis without obstruction of the prosthesis (20%), prosthesis obstruction (30%). Post operative mortality represented 48% (11 patients) 2 due to cholangitis, one due to digestive haemorrhage and 4 due to evolution of carcinoma.

### CONCLUSIONS

The advantage of this new prosthesis is its large internal diameter and the fact that it can be inserted percutaneously.

PO103 THE APPLICATION OF PERORAL CHOLANGIOSCOPY IN BILIARY DISEASES

Oiu Bao-an, Ma Yuan-gui, Huo Feng, Jia Guang-yue

Department of Hepatobiliary Surgery, Naval General Hospital, Beijing, PR China

Peroral cholangioscopy (PC) was first introduced into clinical use in 1974. Due to the technical problems and anatomical difficulties, this method has not yet been widely applied.

In this report, Olympus mother-scope TJF type M20 and baby-scope CHF type B20 were used. Three cases underwent the procedures successfully in a half year period. The results of PC compared with the imaging of endoscopic retrograde cholangiopancreatography (ERCP) were reviewed as follows:

Case I ERCP confirmed the presence of left hepatic duct stones and proximal stricture which were treated by PC after endoscopic sphincterotomy (EST). The proximal stricture was dilated by baby-scope and calculi in situ were removed through the channel of the baby-scope by Dormia basket; Case II was proved to suffer with residual choledocholithiases after cholecystectomy. Following preliminary extraction of stones through duodenoscope channel after EST, PC was used and all residual calculi were removed under direct view; Case III had a history of biliary tract exploration with left hepatic duct stones suspected by ERCP and B-ultrasound imaging. An attempt to directly insert the baby-scope failed prior to EST - even under the guidewire aid. The following procedure carried out immediately after EST was quite easy. Under the direct view of the baby-scope, no calculi could be found and unnecessary laparotomy was therefore avoided.

The preliminary experiences showed that: 1. PC has a definite role in diagnostic and therapeutic biliary endoscopy; 2. EST is a necessary step and should not be omitted even though the insertion of the baby-scope could succeed in some cases; 3. The stricture decreases the effects of PC if the dilation of the stricture cannot be effected. 4. As for the shorter hospitalisation, the total expenditure is lower compared with the surgical interventions.

PO104 **PLACE OF ROUTINE LAPAROSCOPIC CHOLANGIOGRAPHY AT CHOLECYSTECTOMY : A PROSPECTIVE BICENTRIC STUDY**

C. REY* et S. MSIKA**,

*Service de Chirurgie Digestive et Viscèrale, Clinique Sainte Marie, 17 rue Saint Lazare, 27200 Vernon-France, **Service de Chirurgie Digestive et Viscèrale, Centre hospitalier Henri IV, 1 rue du Fort, 78250 Meulan-France.

Development of laparoscopic cholecystectomy has firstly reduced the proportion of routine operative cholangiography because of technical problems. The necessity to obtain a cholangiogram has not to be influenced by the procedure of cholecystectomy. The aim of this study was to appreciate the feasability and performance of laparoscopic cholangiography (LC) during the treatment of symptomatic gallstones.
Between January and December 1992, 100 patients with symptomatic gallstones, eligible for laparoscopic cholecystectomy were prospectively studied. Of these, acute cholecystitis was present in twenty six patients. All patients were operated on by laparoscopic procedure, except 2 conversions to laparotomy. Routine LC was tried in 87 of 100 cases, and was not carried out in 13, because of iodine allergy or a previous endoscopic sphincterotomy. Cholangiography was sucessfull in 72 patients (83%) and failed in 15 patients, because of narrowness of cystic duct in 10 and cystic valves in 5. According to preoperative clinical, biological, ultrasonographic and cholangiographic findings, 68 of 72 cholangiograms were normal. Four were abnormal because of an abnormal biliary anatomy (no cystic duct) in one case, a common bile duct stone in one case and cystic stones in two cases. In the two former cases, a laparotomy was decided for the treatment of anatomic biliary abnormality and common bile duct stone. In the two later cases, laparoscopic cholecystectomy was performed after removal of cystic stones.
In conclusion, LC permits to detect :1) anatomic biliary abnormalities, usually unsuspected on routine preoperative investigations, avoiding severe bile duct injuries. 2) cystic stones, whose laparoscopic treatment is generally easy.3) possibly common bile duct stones, usually identified preoperatively.
LC can be carried out simply and effectively in many cases, but failures are unpredictible. Others preoperative bile duct explorations (endoscopic retrograde cholangio-pancreatography, endoscopic ultrasonography) have to be evaluated.

PO105

### LAPAROSCOPIC CHOLECYSTECTOMY
### (The first thirty five cases)

M.Velegrakis, G.Perpirakis,Th.Kokkinakis,
G.Fasseas, A.Apalakis.

First Department of Surgery, Venizelion
General Hospital, Iraklion Crete GREECE.

From the begining of 1992, laparoscopic cholecystectomy was undertaken on 36 chololethiasic patients, which was successful in 35 of those (97.2%). Criteria for selection of this method were the absence of obstructive jaundice in patient's past history and previous operations in upper abdomen.

In one female patient the laparoscopic method was converted to open operation due do the thickness of the gallbladder wall and to the solid adhesions in Calot's triangle. Hasson's procedure was used in 8 patients because of previous sub-umbilical laparotomies and laparoscopic cholangiography was performed in 3 cases. Retrograde cholecystectomy was carried out in two cases due to difficulties in preparation of cystic duct and artery, which were ligated after the gall bladder was mobilized. Serious intraoperative complications were not observed and the mean operative time was 2 hours.

One patient required prolonged exploration due to bile leak after a presumed diathermy injury of the C.B.D. Two more complications were observed: D.V.T. in one case and sub-cutaneous emphysema in an other one. In all the patients antibiotics were administrated perioperatively, anti-thrombotic agents were also given and sub-hepatic drainage was established in 22 patients for 24 hours. In the rest of patients, the post-operative recovery was uneventful, the average time of hospitalization was two days and the patients returned to their normal activities after one week.

PO106

### LAPAROSCOPIC CHOLECYSTECTOMY IN ACUTE CHOLECYSTITIS COULD BE A SAFE PROCEDURE

Sarwat Mohammad Ali

Minia University, Cairo, Egypt

The past years have been a time of great excitement in laparoscopic surgery and laparoscopic cholecystectomy in particular. Sixty nine patients suffering from acute calcular cholecystitis subjected to laparoscopic procedures were included in this study. Patients were collected at random, 41 patients were women and 28 were men, patients aged 26-84 years with a mean age 58.5 years, laparoscopic cholecystectomy could be done in 54 cases (78.26%). Cholecystectomy was conducted safely in 42 cases (60.87%). In 18 cases laparoscopic cholangiography through cannulation of gallbladder or cystic duct was indicated and it was successful in 14 cases and laparoscopic cholecystectomy was proceeded in 12 cases (17.39%). Minor complications occurred in 9 cases and included bile leakage in 4 cases, umbilical sepsis in 2 cases and 3 cases of abdominal wall haematoma. Major complication in the form of duodenal perforation occurred in one case. In fifteen patients (21.73%) the operation was converted into open procedures because of difficult dissection or unclear anatomy in 8 cases, failed cholangiography in 4 cases, cholangiographic evidenced of common bile duct stones in 2 cases, cystic artery bleeding in one case and perforated duodenum in one case. No mortality occurred in this group of patients.

PO107 **GALLBLADDER AND COMMON BILE DUCT STONES RESULTS OF THE COMBINED ENDOSCOPIC AND LAPAROSCOPIC TREATMENT.**

S Rizos, A Polydorou, G Lakiotis, B Kekis, A Liakos, P Basioukas.

Dept of Surgery, Ag. Anargiri Hospital Kifisia, Athens, GREECE.

The aim of this study is to evaluate the efficacy and safety of the combined endoscopic and laparoscopic treatment of patients with gallbladder and common bile duct stones. For the last 18 months until August 1992, 355 laparoscopic cholecystectomies (LC) were performed. Twenty two patients who had a history of jaundice and abnormal LFTs or dilated CBD on U/S underwent an ERCP before the LC. Fifteen patients had CBD stones which were removed in 14 (93%) patients after a successful endoscopic sphincterotomy. LC was done 1-3 days later. One (7%) patient with large CBD stones (>2.5 cm) required laparotomy with CBD exploration. Intraoperative cholangiography was attempted in 114/355 (32%) patients and was successful in 112/114 (98%). Unsuspected CBD stones were found in 16 (14%) patients. Stones were removed intraoperatively using a small choledochoscope through the cystic duct in 1 (6%) patient. ERCP and EST were performed in 15 (94%) patients with successful stones removal in all patients. CBD access was possible in 3 (20%) patients after a needle-knife papillotomy. Nasobiliary drain was used in 7 patients after the ERCP. One patient (3%) developed mild pancreatitis which was treated conservatively. There were no deaths.
Pre- or post-operative ERCP, EST and CBD clearance combined with Laparoscopic Cholecystectomy is a safe and effective treatment in patients with gallbladder and CBD stones.

PO108 LAPAROSCOPIC CHOLECYSTECTOMY : RESULTS IN 780 CASES

S Rizos, G Alexiou, A Polydorou,

B Kekis, A Liakos

Dept of Surgery, St. Anargiri Hospital,
2nd Dept. of Surgery, Air Force General
Hospital, Athens, GREECE.

Laparoscopic cholecystectomy (LC) has rapidly become an accepted, safe, minimally invasive alternative to open cholecystectomy for the treatment of symptomatic cholelithiasis. We present the results of two major centers where LC is performed routinely the last 2 years. During the period 1991-1992, 780 patients with symptomatic cholelithiasis were referred for LC (M/F 267/513, mean age 52±11 years, range 18-79). The stantard technique using 4 trocars was followed. The operation was completed successfully in 752/780 (96%) patients and was converted to open laparotomy in 28/780 (4%) patients. Reasons for convertion were: technical dificulties in 15 pts, CBD injury in 2 pts, adhesions in 6 pts, bleeding in 1 pt, bilioenteric fistulae in 3 pts, anatomic variations in 1 pt. All patients were given prophylactic antibiotics (Amoxycilin/clavulanic acid). Intraoperative cholangiography was used selectively in 243 (32%) pts. CBD stones were found in 23/243 (10%) pts. Stones were removed intraoperatively through the cystic duct using a small choledochoscope in 1 pt. ERCP, Endoscopic Sphincterotomy (EST) and stone removal were performed in the rest 22 pts 2-5 days after the LC successfully. Preoperative ERCP, EST and CBD clearance were done in 31 pts in whom the diagnosis of CBD stones was made before the operation based in U/S or IV cholangiogram. Mean hospital stay was 2.4±1.3 days. No deaths were reported. Procedure related complications were seen in 17 (2.2%) pts (1 bile leak, 1 subhepatic abscess, 2 trocar injuries resulting sucutaneous hematoma, 7 wound infection, 4 subcutaneous emphysema, 2 CBD injuries). Reoperation for treatment of the complications was necessary in 3/752 (0.4%) pts. Laparoscopic cholecystectomy is a minimal access safe surgical procedure which results in significantly decreased hospital stay, requirement for pain medication, faster return to normal activity and excellent cosmetic results. Combined endoscopic and laparoscopic techniques are also safe and effective in the treatment of patients with gallbladder and CBD stones.

PO109

### LAPAROSCOPIC CHOLECYSTECTOMY - INTRAOPERATIVE DIFFICULTIES AND POSTOPERATIVE COMPLICATIONS

K. Zieniewicz, M. Krawczyk , B. Najnigier

W. Patkowski, P. Nyckowski

Dept. of General Surgery & Liver Diseases ,

Medical Academy of Warsaw , Poland

The aim of the study is the review of intraoperative difficulties and postoperative complications of laparoscopic cholecystectomy [ LCh ].
We have performed 225 LCh in 177 women [ 78,7 % ] and 48 men [ 21,3 % ], aged 16 to 93 years, mean 42,6 years. In addition in 4 cases [ 1,8 % ] the operation was begun with laparoscopic technique and later on was converted to open cholecystectomy. The average operation time was 95 min. No complications of pneumoperitoneum were observed. We did not noticed any complications of introducing the trocars. In 15 cases [ 6,7 % ] the gallblader was opened intraoperatively, including 10 cases [ 4,5 % ] where the stones falled out to peritoneal cavity. The intraoperative bleeding requireing additional clip application was noticed in 4 patients [ 1,8 % ]. In a single case [ 0,4 % ] a few hours lasting bile leakage was observed through the thin drain left in the gallblader bed. It stopped spontaneously. In the other single case the cystic duct in the place of the connection with common hepatic duct was punctered with the hook. The conversion to open cholecystectomy was done and T - tube was inserted in the place of injury . The suppuration of the umbilical wound was diagnosed in 2 cases [ 0,9 % ]. None of the patients required reoperation , we did not observed any lethal complication.
Conclusion : The small number of intraoperative and postoperative complications let us suggest, that LCl should replace the open cholecystectomy in the majority of cases.

PO110

### LAPAROSCOPIC VERSUS OPEN CHOLECYSTECTOMY - RETROSPECTIVE CONTROLLED STUDY

M. Krawczyk, K. Zieniewicz, B. Najnigier
W. Patkowski, P. Nyckowski
Dept. of General Surgery & Liver Diseases,
Medical Academy of Warsaw, Poland

The aim of the study was to compare the results of laparoscopic [ LCH ] and open cholecystectomy [ O Ch ] - effectiveness, indirect costs as well as complications.
The study comprises 225 patients [ group A ] after LCh and the same number - 225 patients after O Ch [ group B ]. The groups were not randomized. Clinical findings in both groups were similar [ A and B respectively ] : recurring biliary colic 59,6 % and 50,7 %, chronic cholecystitis - 28,0 % and 34,7 %, gallblader hydrops 7,6 % and 8,9 % and empyema 4,8 % and 5,7 %. None of the patients of both groups required reoperation. Perioperative antibiotics were administered in mean 1,4 doses in A group and in mean 11,8 doses of cefalosporins in B group. The bile ducts injury was noticed in 1 patient of A group [ 0,4 % ] - the site of connection of the cystic duct and common hepatic duct and in 1 patient of B group - common bile duct injury requireing hepatico - jejunal Roux - en - Y loop anastomosis. Postoperative bile leak from gallblader bed - group A - 1 patient [ 0,4 % ], group B - 4 [ 1,8 % ]. Wound infection occured in 2 patients of group A [ 0,9 % ] and in 9 of group B [ 4,0 % ]. Postoperative pulmonary complications - none in A group, 19 [ 8,5 % ] in B group. Average hospital stay lasted 2,1 days in A group versus 9,6 days in group B.
Conclusion : LCh is a safe procedure, essentialy providing functional benefit to the patients, has a low morbidity rate, decreases total costs of treatment. Thus it should replace O Ch in treatment of symptomatic gallstone diseases.

PO111 **ISOLATED INTRA-ABDOMINAL PANCREATIC TRAUMA**

L.Papastamatiou, P.Vrachnos, D.Xypolytas,

A.Kordonis

Department of Surgery "Apostle Paul" Hosp-KAT
Athens-GREECE

Pancreas being relatively protected in the retroperitoneum presents a low incidence of injury after blunt abdominal trauma. The vast majority of pancreatic traumata are associated with injuries to major vessels, solid and hollow viscera. Isolated pancreatic damage associated or not to extraperitoneal injuries is extremely rare.

Three such cases from a total of 34 pancreatic injuries (8.8%) in a 10-year period are presented. The general pancreatic trauma index in the same period was relatively low (2.8%).

**1st case:** Worker, 37, fall from 3m. No signs of injury. Release after 24h. Readdmission 4d. later. Acute abdomen-shock. He died 3h later during resuscitation-diagnostic procedures.
Autopsy: Necrotizing pancreatitis-total transection of the pancreas.

**2nd case:** Girl, 24, LV1 fracture. Operation 48h later, when clinical signs and CT suspicious. Distal pancreatectomy + splenectomy. Hospitalization 18d, no complications.

**3rd case:** Man, 42, traffic accident. Fracture r.femur. 5th day hypovolemic shock. R.Hypochondrium mass. CT: Haematoma. Operation: Necrotizing pancreatitis. Debridement-wide closed suction drainage. Pancreatic fistula 4 months.

In conclusion isolated intraperitoneal pancreatic injuries are very rare and there are usually diagnosed with delay. Due to this fact fulminant course of acute inflammation or other post-traumatic complications are responsible for the high mortality rates. It is obvious that the mechanism of injury may leed to the suspicion of a potential pancreatic injury which must be followed by an aggressive approach to diagnosis and early operative intervention.

PO112

### PERCUTANEOUS CATHETER DRAINAGE OF A TRAUMATIC COMMUNICATING LIVER CYST - A SUCCESSFUL TREATMENT

M Fuad, S Aman, R Sivanandan, F Abu-Zidan

Department of Surgery & Radiology, Mubarak Al-Kabeer Hospital, Kuwait

A previously healthy 30 year old male sustained blunt trauma to the right upper quadrant of the abdomen. Ten days later, he developed progressive obstructive jaundice with raised liver enzymes. Ultrasound and CT scan of the abdomen revealed a cystic lesion in the right liver lobe of 15 cm diameter with intrahepatic biliary ducts and dilatation of both lobes.

ERCP showed normal CBD with external compression on common hepatic duct and both hepatic ducts.

Cook's pig tail (10.2F) percutaneous catheter drain under ultrasound guidance was fixed. Mixed blood and bile was initially drained. Later on it became clear bile.

Repeat ERCP after 2 weeks revealed a normal biliary tree with a minute communication between right hepatic duct radicle and the cavity. The catheter was removed after 1 month. Repeat CT scan showed complete collapse of the cavity. Percutaneous catheter drainage was a simple successful procedure that obviated surgery.

PO113

### THE MANAGEMENT OF LIVER INJURIES IN A LOCAL SURGICAL DEPARTMENT

G Perpirakis, M Velegrakis, S Kandilakis, F Seferiadis, N Paraskakis

Surgical Department, of General Hospital of Rethimnon, Greece liver trauma is not frequent but its management is usually very difficult and its outcome is combined with high mortality.

Over a 7 year period from 1986, twenty-five patients with hepatic trauma were managed operatively in our department. Twenty patients had blunt and 5 penetrating liver trauma, including 2 gunshot wounds. 17 of 20 blunt traumas were caused by road traffic accidents, with direct hepatic damage and 3 were due to a fall. Three cases were pure hepatic ruptures and 22 were related to multiple injuries. The hepatic injuries were classified according to severity and regarded as being grade I in 3 cases, grade II in 14, grade III in 5 and grade IV in 3 cases according to the classification of Calne.

All the above patients required urgent laparotomy. The 3 cases of grade I,the 14 of grade II and 2 of 5 cases of grade III were treated with lavage of hepatic parenchyma with normal saline by suturing profound ruptures and by stable pressure of the liver to control haemorrhage. 2 cases of grade III required excision of the damaged part of liver and ligation of bleeding vessels. One patient of grade III and in 3 of grade IV required perihepatic packing for life saving bleeding control. Of those 4 cases, 1 died during the operation as a result of severe co-existing injuries, 3 were referred to a specialist centre. Of the 21 cases re-operation was required in 2 for haemorrhage and 2 for sepsis. Of the 25 patients, 5 died. Only 5 out of 25 (20%) died. Grade IV 2 patients, grade III 2, and 1 patient of grade II. The cause of death in 3 cases was uncontrollable haemorrhage, coagulopathy and in the other 2 cases the cause of death was severe co-existing injuries.

The successful outcome of these injuries depends on the resuscitation and the urgent laparotomy, in which conservative surgical procedures must be used. We conclude that those simple surgical procedures such as direct sutures, ligating bleeding vessels, excision of damaged hepatic parenchyma if necessary, perihepatic packing and selective hepaticartery ligation. However the morbidity and mortality from these injuries is still high in all centres specialised or not.

PO114

### INTRAPANCREATIC RUPTURE OF THE BILE DUCT

A Depolo, M Rubinić, N Irvaniš, N Petrošić.

University Surgical and Internal clinic, Rijeka, Croatia

A thirty-one year old patient was admitted to hospital after a car accident. The patient had multiple bone injuries and signs of head injury. During the first hours of hospitalisation he developed an abdominal pain in the supra-umbilical region. Ultrasound showed minimal free liquid in the abdominal cavity. There was no sign of an acute abdomen. Two days later ultrasound was normal. The patient was treated because of bone injury.

Seven days after admission the patient became jaundiced and liver function tests showed the evidence of a bile duct obstruction. On ultrasound we saw dilated bile ducts and ERCP showed amputation of the choledochal duct in the intrapancreatic portion and dilated pancreatic duct. CT scan showed enlarged head of the pancreas with necrotic changes and dilated bile ducts. Body and tail of the pancreas were normal and the pancreatic duct was dilated.

During the surgical procedure intra-operative cholangiography showed "stop" in the head of the pancreas and swelling and hardness of the head of the pancreas with multiple adhesions. Choledochojejunostomy with "Roux" loop was performed with good results. Six months after the operation the patient is without trouble and at this time we planned to perform a second operation (pancreaticojejunostomy) if the stenosis of the pancreatic duct in the head of the pancreas developed dilatation of Wirsung's duct, and chronic pancreatitis.

Two years after the operation the condition of the patient is satisfactory, and liver function tests are normal. On ultrasound there is no evidence of dilatation of the pancreatic duct.

PO115 LIVER TRAUMA

K.Kindylidis,F.Seferiadis,D.Bastas
N.Lambrinaki,G.Garinis,V.Mouzakis
G.Velmachos
"Sotiria" General Hospital,Surgical
Unit,Athens,Greece

Hepatic injuries,relativery rare in peacetime in the past,are becoming more common nowadays because of the enormous increase of traffic accidents.Injuries of the liver represent a very severe clinical condition,both from the point of view of immediate management of liver injuries which requires the taking of rapid,sound and correct decision as to the appropiate operative procedure to be followed in each specific case.
Open lesions of the liver are less difficult to handle,with the exception of those cases where we have associated lesions of a large vessel,e.g.the inferior vena cava of the aorta.
In contrast,several problems arise in the management of closed hepatic injuries which are still associated with high mortality and complication rates.
There were reviewed 48 cases with traumatic injuries of the liver. The over all mortality rate was 29.16%.Eleven from the patients who died during the operation or the postoperative period suffered from minor or moderate liver injuries treated by simple suture. Deaths were mainly due to multiple organ inguries or oligemic shock than to the hepatic injuru itself.The majority of liver injuries may be safely managed with present knowledges on hepatic surgery with exception injuries involving hepatic veins and retrohepatic vena caval area.

PO116

### OUR EXPERIENCE IN THE TREATMENT OF TRAUMA OF THE HEPATO-PANCREATO-BILARY SYSTEM

I. Viachky, N. Iaranov, Arc. Ivanov, A. Mitov

"Bialo more" str. No8 - Sofia; University Hospital
"Queen Joanna" - Centre of Urgent Medical Aid
Bulgaria

In Bulgaria trauma of the abdomen for the last 10 years rates 4th place as a reason for death. 50% of traumatic injuries are caused by transport vehicles and firearms. According to the data injuries of the liver are from 5 to 11% of abdominal trauma. Trauma of the extrahepatic bile ducts is comparatively rare, which is due to their anatomical location. Penetrating injuries of the pancreas are less frequent than blunt injuries.

For the period 1985-1992 in our surgical clinic we treated 164 patients with trauma of the abdominal system. 132 were blunt injuries and 42 were penetrating injuries. The male patients predominated in number - ie. 101. The age of the patients varied between 16 and 70 years. Trauma of the HPB system was mainly multiple. In 13 cases we observed single liver trauma with 15 cases of post traumatic pancreatitis. Single trauma of the extrahepatic bile ducts was found in 3 patients and injury of the gallbladder in 1 case. Diagnosis, re-animation and operation are done at the same time in our practice. Treatment is done on the basis of 2 principles - to obtain control of the intra-abdominal bleeding and abdominal sepsis.

Our data shows that trauma of the HPB system is 15, 1% of the patients with abdominal trauma with a mortality rate of 20, 3%.

PO117 COMPARISON OF THREE CLASSIFICATION SYSTEMS FOR LIVER TRAUMA

SS Hanna, S Rizoli

Division of General Surgery
Sunnybrook Health Science Centre
University of Toronto, Toronto, Canada

The aim of this study was to compare 3 widely accepted classification systems for liver trauma Moore (1), OIS-AAST (2), and Buechter (3) to see which was superior.

We retrospectively classified all 172 patients with liver trauma admitted to Sunnybrook Regional Trauma Unit from Jan 1, 1987 - June 30, 1992. 90% were due to blunt trauma and 87% underwent laparotomy. 34.5% had liver related complications and 6.4% had liver related death. We compared liver related mortality and morbidity (M & M). Morbidity was evaluated by measuring blood tranfusion requirement, liver complications and liver treatment using the 3 different classification systems. Statistical analysis revealed that each of the 3 systems was internally consistent and had a linear relationship to each variable in predictng M & M.

The Moore was the best predictor of liver related death and blood requirement, the OIS and the Buechter were able to predict major liver treatment better and the Buechter predicted liver complications well. Subjectively the Buechter is easier to use due to its simplicity; but it tends to cluster most of the patients into grade 2 and the choice of treatment affects the grade of injury. The OIS, although more complex, leads to a better separation of the different grades of liver trauma; thus allowing comparison between different institutions.

In conclusion, all 3 systems have merit and none proved superior in all aspects. The major advantage of Buechter is its simplicity and ease of recall. The Moore is closely related to the OIS which is more accurate and allows precise comparisons between different institutions.
1. Moore EE: Critical Decisions in the Management of Hepatic Trauma. American Journal of Surgery 148: 712, 1984.
2. Moore EE, Shackford SR, Paliter HC et al: Organ Injury Scaling (OIS) Spleen Liver and Kidney. Journal of Trauma 29: 1664, 1989.
3. Buechter KJ, Zeppa R, Gomez G: The Use of Segmental Anatomy for an Operative Classification of Liver Injuries: Ann Surg 211: 669, 1990.

### PO118 MORTALITY AND MORBIDITY OF PANCREATIC TRAUMA

L.Papastamatiou, L.Lapidakis, P.Vrachnos, A.Zarzali

Department of Surgery "Apostle Paul" Hosp-KAT Athens-GREECE

The high frequency of traffic accidents has contributed to an increasing incidence of pancreatic trauma. The surgical adage "the pancreas is not your friend" is apparent considering mortality and morbidity rate over than the one half of pancreatic trauma patients. As associated major vascular or solid organ injuries lead to early mortality due to exsanguination, mortality rate of pancreatic trauma due mostly to complications, is easily understood.

During the last decade 34 cases of pancreatic trauma after abdominal injuries were treated. The spectrum of injuries comprised contusion with haematomas, laceration, fracture and complete disruption of the gland, but the main point to determine trauma severity was the integrity of the pancreatic duct. Associated intra-abdominal trauma rate exceeded 2.5 per patient. Operative techniques used were wide closed suction drainage, debridement, suture, distal or hemipancreatectomy and pancreatoduodenectomy.

Hypovolemic shock was the cause of death in 10 patients due to associated injuries. In another 14 patients secondary haemorrhage, fistula, pseudocyst or intraabdominal abscess formation, and anastomotic leakage obliged to reoperations, but finally 8 patients died because of sepsis or multiple organ failure.

It is concluded that the final outcome after pancreatic trauma is in close relation with exact intraoperative evaluation of the injury, especially of the integrity or not of the major duct and is also depended on the degree of parenchymal damage and the anatomic location of the injury.

PO119

### BACTERIAL TRANSLOCATION FROM GUT IN ACUTE LIVER INJURY INDUCED BY D-GALACTOSAMINE

F B Kasravi, X Wang, W Quan, W Guo, G Molin, R Andersson, B Jeppsson, S Bengmark

Departs Surgery, Zoophysiology and Food Chemistry, Lund University, Lund, Sweden

Bacterial translocation from the gut is thought to be responsible for infectious complications in surgery and in different disease states. We studied the translocation of bacteria in galactosamine induced liver injury and compared the results to liver resection and control rats.

Material and methods: Liver injury was induced by administration of D-galactosamine. Results were compared to 70 % liver resection and control rats. Animals were studied 24 and 48 h after injury. The extent of liver injury was assessed by liver function tests and histology. Translocation to arterial and portal blood as well as solid organs was studied after administration of labeled bacteria. Intestinal permeability was also measured by sodium fluoroscein administration. Bacterial cultures of intestinal mucosa and content was performed.

Results: The incidence of bacterial translocation to arterial and portal blood was found to be 50% and 83% resp. after galactosamine administraion at 24h and remained elevated to a similar extent at 48h. The rate of translocation after liver resection was 100% to arterial and portal blood at 24h and dropped to 50% at 48h. There was no translocation to blood in control group. Bacterial translocation to liver, spleen and mesenteric lymph nodes was 100% after both galactosamine administration and liver resection at 24 and 48h. Intestinal permeability was increased similarly in both groups compared to control animals. Bacterial cultures did not show any differences between the groups.

Conclusion: Bacterial translocation and intestinal permeability are increased after severe hepatocellular damage to a similar extent as seen after liver resection. They may play a pathogenetic role in the infectious complications seen in these conditions.

PO120

### RISK OF CONTACT WITH HEPATITIS B AND C VIRUS AND HIV IN A SAMPLE OF NO RISK

J Alvarez, Maillo, R Lobato, J Fradejas, Marin, M Martin, M Moreno Azcoita

Hospital Universitario de Getafe, Madrid, Spain

The most important measure to prevent HIV and hepatitis transmission includes avoiding accidental needle puncture, exposure to injured mucous membrane, and patients' blood. But sometimes this is unavoidable so the estimation of risk for the surgical team needs to be known.

Objective. To determine the prevalence of hepatitis B, hepatitis C, and HIV infection in outpatients prior to surgical treatment.

Patients and Methods. We present preliminary results of a prospective study in patients aged between 15 and 60 years. The pre-operative studies included a serological control for hepatitis B, C, and HIV, with prior consent. Patients were asked for some identifying risk factor: drug-addict (DA), homosexual (HS) or blood transfusion (BT).

Results. We conclude that of the 262 patients screened, 73.5% male, mean age 38.1 ± 12.1. Group of risk: DA 10p. (3.9%), HS 4p. (1.6%), BT 10p. (3.9%). Serological results were as follows:

| | HBsAG | HBsAb | HBcAb | HBeAg | HBeAB | HC | HIV |
|---|---|---|---|---|---|---|---|
| N | 9 | 16 | 29 | 8 | 15 | 1 | 6 |
| % | 3.5% | 6.2% | 11.2% | 3.1% | 5.8% | 0.4% | 2.3% |

| Hepatitis B: | active | cured | Hepatitis C | HIV |
|---|---|---|---|---|
| DA | 1c. 10% | 1c. 10% | 0c. 0% | 3c. 30% |
| HS | 0c. 0% | 0c. 0% | 0c. 0% | 2c. 50% |
| NO RISK | 12c. 4.8% | 13c. 5.2% | 1c. 0.4% | 1c. 0.4% |

Conclusion. The risk of contact with hepatitis B and C virus, and HIV has shown to be higher than expected values, up to 4.8% of hepatitis B, 0.4% of hepatitis C, and 0.4% of HIV in the no-risk group.

PO121

### EARLY OPERATIVE VERSUS NONOPERATIVE TREATMENT OF SEVERE ACUTE PANCREATITIS

A Grubisic

Surgical Unit, General Hospital
Jesenice, Republic of Slovenia

A 12 year review of 76 patients with severe acute pancreatitis was performed. The severity of the disease was evaluated objectively by a modified scheme based on generally accepted criteria. An operation was performed upon 47 patients(62%) within three days form the onset of symptoms while the rest were managed conservatively only. Since strictly defined indications for the early operation were used, a prospective evaluation of results could be established. The standard operative procedure included debridement of demarcated necrosis, wide decompression and prolonged perfusion drainage of pancreatic bed and affected retroperitoneal spaces. No resections of the gland were done.
An overall mortality of 43,4% was observed. However, mortality was significantly higher (75,9%) in patients treated nonoperatively only compared to those who also underwent an early operation(23,4% mortality).
While vigorous and well-grounded conservative management undoubtedly remains of paramount importance, more agressive surgical approach in the early phase of severe acute pancreatitis is also suggested. The rationale for this is primarily peripancreatic compartment decompression with thorough and continuous elimination of toxic pancreatic exudate. Since survival rates were improved considerably by pursuing such strategy in selected cases, it is concluded that many otherwise doomed patients may benefit from early operation and thus be saved. The fact that in 73,7% of reviewed patients the necrotizing pancreatitis was found histologically, supports such conclusion.

PO122

### COMPARISON OF THE INSULINOTROPIC EFFECTS OF OMEGA-3 AND OMEGA-6 FATTY ACIDS

EC Opara, SK Lee, M Garfinkel, OE Akwari

Department of Surgery, Duke University Medical Center, Durham, North Carolina 27710, USA

Although there is a great deal of enthusiasm for the consumption of fish oil enriched with omega-3 ($\omega$-3) fatty acids, it is known that prolonged provision of marine oils to subjects with type II (adult-onset) diabetes mellitus may raise their daily insulin requirements. Also, it has been shown that acute exposure of pancreatic islets to high concentrations of polyunsaturated fatty acids will stimulate insulin secretion whereas chronic exposure to these fatty acids will desensitize the islets to glucose via a mechanism linked to fatty acid oxidation. However, it is not clear whether omega-3 ($\omega$-3) fatty acids present in fish oil have a direct effect on the release of insulin from pancreatic beta cells. AIM: The purpose of the present study was to examine the effects on insulin release of a dietary precursor of $\omega$ -3 fatty acids, linolenate (18:3, $\omega$3) and the main constituent $\omega$ -3 fatty acid of fish oil, eicosapentaenoic acid (EPA, 18:5 $\omega$ -3), and to compare these effects with those of omega-6 fatty acids. METHOD: In each experiment, a total of six islets microdissected from the pancreata of three female CD-1 albino mice were placed in a flow-through perifusion chamber and preperifused for 1 hour, at the rate of 1ml/min with a Krebs-Ringer bicarbonate buffer pH 7.4, containing 2% bovine albumin, 5.5mM (basal) glucose which was continuously gassed with 95%/5% $O_2/CO_2$. After basal samples were taken, the perifusion was continued in 20 mins random cycles of different fatty acids separated by 20 min "washout" periods of basal glucose perifusion. Solutions were changed using a stopcock and effluent perifusate samples collected on ice at 2 min intervals were stored frozen until radioimmunoassay for insulin. RESULTS: Insulin secretion in response to the different fatty acids was assessed as the mean integrated area under the curve (AUC/20 mins) above basal. Perifusion of islets with 5mM of each of linoleate (18:2, $\omega$ -6) and linolenate (18:3 $\omega$-3) showed that the latter was more potent insulin secretagogue as the AUC's respectively were 2842±417 and 10506±1490 pg/20 mins, $p<0.002$, n=5). Also, 1mM EPA (20:5, -3) was significantly more potent than 1mM arachidonic acid (20:4,$\omega$-6) in insulin stimulation (2168±452 vs 597±251 pg/20 min, respectively, $p<0.003$, n=5). CONCLUSION: These data demonstrate a direct effect of omega-3 fatty acids on insulin secretion by perifused islets and suggest that $\omega$ -3 fatty acids are more potent insulin secretagogues than the corresponding $\omega$ - 6 fatty acids.

PO123

### ANEURISMECTOMY WITH MICROSURGICAL RECONSTRUCTION OF SPLENIC HILUM

P.C. GIULIANOTTI, A. COSTA, T. BALESTRACCI, U. BOGGI, D. MAZZOTTA, A. CAROBBI, M. FERRARI, F. MOSCA
Istituto di Chirurgia Generale e Sperimentale
Pisa University Medical School, Italy

Aneurysms of the splenic hilum often require splectomy, which has been reported to be associated with a high incidence of septic complications (1-4%). Utilizing microsurgical techniques for vascular reconstruction, splenic preservation may be technically feasible even in case of hilar aneurysm.

CASE REPORT: B.P. 37-year-old-female with no past history of vascular disease, 2 regular pregnancies, came to our observation for an asyntomatic aneurysm of the splenic hilum (diameter 1.5 cm) incidentally detected at ecotomography. Because of the increasing volume of the aneurysm (diameter 2.1 cm) after one year of clinical follow-up the operation was decided.

Because of the overlapping of vessel images selective angiography was not diagnostic; ecotomography with pulse doppler confirmed the diagnosis of hilar aneurysm involving the bifurcation of the splenic artery (figure A).

Through a midline incision with self retractor the spleen was anteriorly displaced sectioning the posterior legament. The aneurysm was completely mobilized and resected working from the posterior aspect of the spleen. Using an operating microscope (Olympus.[illegible]) the two splenic efferent vessels were anastomosed end to end (7/0 prolene) and the afferent artery anastomosed to the loop end to side (figureB). The post-operative course was uneventful. A post-op. digital angiography showed the excellent splenic circulation with a normal vascular anatomy.

Microsurgical technique can be used also in general surgery for difficult vascular reconstructions.

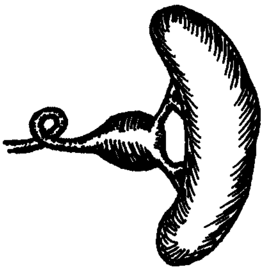

Figure A

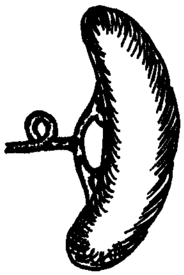

Figure B

PO124

### ACUTE NECROTIZING PANCREATITIS: EXPERIENCE OVER A SEVEN YEAR PERIOD

G Perpirakis, M Velegrakis, N Nikolakakis, B Chrisochoos, A Apalakis

1st Surgical Department, Venizelion Hospital, Surgical Department of General Hospital of Rethimnon, Greece

Survival after an acute pancreatitis attack, has improved in the early stage of the disease as a result of aggressive organ support, over the past two decades. But patients continue to die at a later stage from necrotic and septic complications. The natural history of necrotizing pancreatitis has been better elucidated, with appreciation of a spectrum of pancreatic necrosis and pancreatic abscess; infected pseudocyst and peripancreatic phlegmon are excluded from the latter term.

Over a 7 year period from 1986 to 1992, 196 patients with an acute pancreatitis attack were treated in our units, including 24 cases (12, 25%) with acute necrotising pancreatitis (18 men and 6 women). Pre-operatively the diagnosis in 22 cases (91,5%) was documented by combined estimation of the clinical course, laboratory evaluation and by findings on C/T. In all those patients high levels of serum CRP (>200mg/lt) leucocytosis and positive limulus amoebocyte lysate test for detecting endotoxinemia, were found. Intra-operatively findings were: 6(25%) cases with pancreatic necrosis, 10(41.6) cases with pancreatic abscess 3 on the pancreas head and 7 on the pancreas body and tail and 8 infected.

All patients with documented necrotising pancreatitis underwent operative management via a midline incision and trangastrocolic approach to the lesser sac. Our surgical method is based on atraumatic necrosectomy and lesser sac drainage using 4 large diameter (20 mm) silastic tubes, for continuous post-operative lavage. The average amount of fluid used was approximately 4 litres per day, for 12±2,3 days. Four of 24 patients (16,66%) developed a recurrent intra-abdominal abscess, requiring re-operation. Total mortality was 8 of 24 patients (33,33%), including 4 cases with pancreatic abscess, 3 with infected pancreatic necrosis. In 7 cases the death was the multiple organ failure syndrome and in another one it was massive pulmonary embolisation. Later complications were observed in 4 of 16 (25%) survivals, which analytically were: 2 pancreatocutaneous fistulae, 1 pseudocyst formation and 2 exocrine pancreatic insufficiency.

We conclude that the surgical management in patients with acute necrotising pancreatitis is a difficult decision and requires clinical judgment with a thorough consideration of the clinical situation and other non-operative means of supportive care. The proposed method is necrosectomy accompanied by post operative lavage to avoid severe operative complications, such as severe intra-abdominal bleeding and bowel necrosis, which increases morbidity and mortality in these patients.

PO125

### SURGICAL MANAGEMENT OF PANCREATIC PSEUDOCYSTS. A DECADE OF EXPERIENCE.

P.Alfaras, K.T.Delis, M.Stoccos,

S.Drakopoulos, E.J.Hadjiyannakis.

A'Surgical Department of the GH
"The Evangelismos of Athens".

Pseudocysts complicate ac.pancreatitis in 2-3% and at least 55% of these mandate surgical intervention. This work,on a retrospective basis,aims at presenting the material,results and experiencies gained through the surgical management of pancreatic pseudocysts,within the framework of General Surgery,over the last decade.

During this period,we managed a total of 24 patients presenting with surgical complications of ac.pancreatitis,their ages ranging from 34-83 years,yielding a mean age of 56 years.Of this total,in 19 cases (79,16%) the cause was pancreatic pseudocysts.Of this,in 13/19 patients (68,4%) only a single cyst was detected, the group of multiple cysts representing the rest (6/19 or 31,6%). Elective operations,following complete preparation,were registered in 16 cases,whereas emergency surgery was indicated in 3 subjects.

All elective cases (16),enabled us to perform internal drainage operations,either in the form of cystogastrostomy (15 patients), or in the form of cystojejunostomy (Roux en Y - 1 patient). The emergency cohort,was managed either through external drainage being exemplified in 2 cases with immature cyst walls and inflammation,or through internal drainage,as it occurred in one subject with a spacious double cyst which we drained in the stomach (cystogastrostomy),in the presence of a large subphrenic abscess which we drained externally.

Our operations had had a very satisfactory course,in the majority of cases,with early mobilization of the patients (2-3rd day) and oral feeding (8-10th day),following upper GI series.In the complications we should refer to 1/ an iatrogenic splenectomy, 2/ two cases of gastric dysfunction (one reoperation),3/one postoperative bleeding (reoperation),4/ an anterior gastrotomy rupture,effecting in a spontaneous external fistula (on grounds of an emergency cystogastrostomy),5/ one death caused by exacerbation of a concomitant hematological disease.

In conclusion A/Elective treatment of p.pseudocysts presents a very good postoperative course, B/Cystogastrostomies have been the most easily performed and frequent operations of internal drainage,C/ On grounds of inflammation,external drainage constitutes the treatment of choice,D/Emergency early operation of p. pseudo cysts seems to result in high morbodity and prolonged hospital stay,E/The type of internal drainage is determined by the volume and location of the cyst.

PO126

### THE USE OF ROUX-EN-Y JEJUNAL LOOP IN THE MANAGEMENT OF PANCREATIC PSEUDOCYSTS

Kr Daskalakis, Th Pavlis, V Christidis, G Diamantopoulos

3d Surgical Department, Evangelismos Hospital, Athens, Greece

Pseudocysts of the pancreas may be post-inflammatory or post-traumatic. Elective operation is indicated if the pseudocyst is bigger than 4 cm in diameter and after unsuccessful conservative treatment for 6 weeks.
The choice of operation depends on the size, nature, situation and type of the cyst.
In the 3d Surgical Department of "EVANGELISMOS" Hospital, 32 patients with pancreatic pseudocysts were operated on the last 10 years (1983-1992). Twenty of these were female and 12 male. Their age ranged between 25 to 65 years. The cause of the pseudocyst was acute pancreatitis in 24 patients, trauma in 2 and unknown in 5. Eight patients with spontaneous regression of the pseudocyst are not included in this study.
Internal drainage was performed in 27 patients: in 4 patients a cysto-gastrostomy, in 22 a cysto-jejunostomy using a Roux-en-Y jejunal loop and in 1 a cysto-jejunostomy with a simple jejunal loop with jejuno-jejunal anastomosis (Braun) was performed.
External drainage of the pseudocyst was done surgically in 3 patients because of infection. Two of them reoperated on after a few months using a Roux-en-Y jejunal loop and the other one has had spontaneous healing of the remaining cyst and the pancreatocutaneous fistulla.
Two distal pancreatectomies without splenectomy were performed. There were no postoperative deaths in this series. The early and late results are good.
It is concluded that in our opinion, if the removal of the pseudocyst is impossible, internal drainage using a Roux-en-Y jejunal loop is the treatment of choice.

PO127

### SCORING SYSTEMS FOR PREDICTION OF SEVERITY IN ACUTE PANCREATITIS

D.D. Tran, M.A. Cuesta

Department of Surgery, Free University Hospital, Amsterdam, The Netherlands.

Despite better understanding in the pathophysiology of acute pancreatitis and improvements in supportive care in the past decades, the mortality in patients with the severe form of this disease remains high. Early recognition of the high risk patients is thus mandatory in order to improve prognosis. We compared the multiple organ system failure (MOSF) score, the APACHE II, Ranson and Imrie scores for their predictive value in evaluating severity of acute pancreatitis. The clinical outcome was graded as mild if the course was uncomplicated, and as severe if there were local complications -i.e. (peri-) pancreatic necrosis, pseudocyst or abscess- or a lethal outcome.

Of the 259 patients, 73 (28%) had severe disease; of these 59 (23%) developed local complications and 45 (17%) died. Fifty-two (20%) had OSF (at least one OSF) on admission with substantial mortality (75% vs. 3% in those without OSF, $p<0.001$). Sixty-five percent of patients with local complications and 87% of nonsurvivors had OSF. On admission, only MOSF and APACHE II scores were available. MOSF score correctly predicted in 64% of patients with severe disease (and in 53% of patients with local complications and 87% of nonsurvivors). APACHE II correctly predicted in 60% of patients with severe disease (and in 51% of patients with local complications and 93% of nonsurvivors). After 48 hours, MOSF score was most sensitive and correctly predicted severity in 67% of patients, compared to about 60% for other scores (and in about 55% of patients with local complications and 90% of nonsurvivors). Of the four scoring systems, only MOSF and APACHE II scores allowed repetitive assessment to monitor the course of the disease.

In conclusion, 1) MOSF score is of similar value as the APACHE II score and the multiple-factor scoring systems in early identification of high risk patients with acute pancreatitis. 2) Only MOSF and APACHE II scores are available soon after admission and they allow close monitoring in these patients and may therefore help in deciding on therapy.

PO128

### NECROTIZING PANCREATITIS AND SECONDARY PANCREATIC INFECTION SURGERY

L García Flórez, JJ González,

JI Rodríguez, A Martínez

Departamento de Cirugía, Hospital
Central de Asturias, Oviedo, Spain.

Infected pancreatic necrosis (IPN), pancreatic abscesses (PA) and infected pseudocysts (IP) are not equivalent conditions although they represent different manifestations of the same disease. Depending on the microorganisms virulence and the patient s level of defenses an infection can remain localized (abscess) or extensively propagate throughout the devitalized tissue.

In this work, the results of necrotizing pancreatitis surgery in 30 consecutive patients admitted to our Department of Surgery between January 1988 and October 1992 are presented. Twenty patients were males and 10 females, with an average age of 58.2+/-14.6 years (range 28-87). The mean hospitalization period was 36.7+/-31.4 days. Most frecuent among the etiologies presented was alcohol with 14 cases, followed by biliary lithiasis with 9, idiopathic 4, postoperative 2 and hyperlipidemia 1. Twenty two patients presented pancreatic and/or peripancreatic infection either on admission or during hospitalization (13 PA, 7 IPN, and 2 IP). Surgical interventions (1 to 5 per patient) included operations over the pancreatic area in 39 cases (28 drainages and/or lavages, 9 necrosectomies, 2 distal resections with splenectomy), 15 on gallbladder and biliary tract (4 cholecystectomies, 7 cholecystostomies, 4 drainages of the common bile duct) and 6 on the digestive tract (3 colostomies, 2 jejunostomies and 1 gastrostomy). Seventeen patients died.

Clinical, bacteriological, radiological findings and the results of treatment are analyzed. It is concluded that pancreatic infection secondary to necrotizing pancreatitis is associated with a high morbidity and mortality related to its clinical presentation (localized vs. diffuse, p=0.04). The APACHE II severity of illness index is considered to be an accurate predictor of mortality (p=0.0005).

PO129 THE TREATMENT OF PANCREATIC FISTULAS USING BIOLOGICAL SEALANTS

V. Costantino, A.Alfano D'Andrea, C.Sperti,
F. Di Prima, S. Pedrazzoli.

Semeiotica Chirurgica, University of Padova,
Italy.

Fistulas are a frequent complication in parcreatic surgery. We think that their treatment must include an adequate drainage, the functional suppression of the pancreatic gland, a careful evaluation of all the nutritional parameters and surgical treatment in selected cases. We performed all the above conservative techniques in order to achieve a good healing of the fistulas we observed. In addition we used a human fibrin sealant to fill their tracts. Our overall experience in the treatment of fistulas with fibrin sealant includes 9 enteric, 2 biliary, 1 vaginal and 13 pancreatic fistulas. Since 1984, 13 pancreatic fistulas underwent fibrin sealing: 6 followed a pancreatico-duodenectomy (2 for cancer, 2 for papillary carcinoma, 2 for endocrine tumors); 2 after left pancreatectomy (1 for chronic pancreatitis, 1 for cystoadenoma); 1 followed a pancreaticojejunostomy for chronic pancreatitis; 1 after excision of an insulinoma in the pancreatic head; 3 after surgery for acute pancreatitis (1 necrosectomy and drainage, 1 percutaneous drainage of pseudocyst, 1 cysto-jejunostomy). All our patients received an adequate nutritional support and had their pancreatic secretions reduced by pharmacological treatment. Moreover, they were all submitted to repeated X-ray controls in order to position an accurate and proper drainage. As soon as a regular tract and a low outflow were achieved, the patient underwent the sealing treatment. We used a double lumen catheter under X-ray control which permitted a selective injection of the sealant at the origin of the fistula up to the skin. The tract was thereby completely filled. In 11 cases we obtained a good healing with a single injection; 2 patients required 2 treatments. The sealant is self-shaping and its pressure prevents the out-flow of secretions through the fistula, diverting them into their natural channels. Finally, the components of the sealant support the growth of a good scar tissue. The results we obtained by this technique can be considered satisfactory as some patients recovered without any surgical treatment which would have been otherwise required.

PO130 Treatment of "Acute Pancreatitis"

H.Wenk, I.Kunze, H.-P.Bruch

Klinik für Chirurgie der Med.Universität zu Lübeck, Germany

From 1.1.87 to 31.12.90 37,5% of 80 patients, admitted for acute pancreatitis, had an early operation (n=30), another 7,5% (n=6) had elective operation after improved clinical status. Biliary Pancreatitis was mainly treated by endoscopic sphincterotomy/cholecystectomy (81,3%), patients with nonbiliary pancreatitis had more conservative treatment (81,3%). Lethality of patients was 5%, 51,4% of the patients with necrotising pancreatitis had successful conservative treatment. Indications to operate included expected gallstones or non-sufficient endoscopic treatment of biliary pancreatitis as well as apparently infected necrosis of the pancreas. In this connection the clinical picture of the patients in correlation with the CT-diagnosis and the biochemical parameteres is the decision factor.

PO131

### STUDY OF THE ENDOGENOUS SYSTEMS FOR PROTEASES INHIBITORS IN ACUTE PANCREATITIS

S.Pierrakakis,P.Karydakis,A.Ninos,G.Sabalis,
N.Economou,G.Antsaklis.
Surgical Depatment,Sismanoglion General Hospital,
Athens-Greece.

The goal in our study is to determine the fluctuations of the endogenous antiproteasic activity of the patients' serum with acute pancreatitis in all the stages of the disease,as well to notify the corellation of these fluctuations to the special characteristics of acute pancreatitis.Motives in our study is the knowledge of the endogenous ability of the organism to inactivate proteolytic enzymes using special groups of proteases inactivators in the plasma as are a2-macrogloboulin and a1-antithrypsin systems.
We studied 30 patients with acute pancreatitis of different causes.We evaluated these systems in all stages of the disease according to the therapeutical protocol of the clinic (FFP, aprotinin, corticosteroids and in case surgical intervention).
An immunodiffusion method was used for the laboratory determination of the antiproteasic systems.The conclusions of our study are:a)The system of a2-macrogloboulin decreases to 65% in the acute phase b)The system of a1-antithrypsin rises at the first 72 hours c)The quantitative alterations of these systems are in accordance to the progression of the disease (evaluation made by Ranson's criteria) d)These fluctuations are markers of the therapeutical responsiveness.

PO132

### FACTORS INDUCING MULTIPLE-ORGAN -FAILURE AFTER OPERATION FOR ACUTE HAEMORRHAGIC-NECROTIZING PANCREATITIS

Yang Sen-hua, Yang Yi-cun, Sun Bian-sheng

Department of Surgery, Chengdu Third People's Hospital, Chengdu, Sichuan, China

The incidence of multiple-organ-failure (MOF) after emergency surgery (30-50% for intra-abdominal sepsis) was 7-22% with a mortality of 30-100%. According to Borzotta's criteria MOF occurred in 19/95 cases (20%) treated surgically in the last 10 years and 10(52.63%) died. We suppose some attributive factors inducing MOF were: (1) Acute haemorrhagic-necrotizing pancreatitis (AHNP) - a serious disease which is characterised by the enzyme "broth" of many components, formed in and around the pancreas. MOF in 11/19 cases (57.59%) occurred simultaneously and the average time of evolution was 3.5 days post-operatively, much earlier than that (M 30.5 days) after admission by Eiesman, suggesting AHNP has a serious systemic pathologic process with prompt influence on multi-organ functions. (2) Shock was common. 9/20 cases with shock had MOF resulting in 3 deaths. Copious ascites, myocardial inhibitory factor and endotoxin released and absorbed and the so-called "enzymatic" shock all contributed to cause abrupt reduction of blood volume, cardiac output and stress of peripheric blood vessels and hindrance of micro-circulation; thus disturbance and failure of organ function resulted. (3) Infection - pancreatic or peripancreatic abscess "enzymatic abscess" is the most frequent post operative local complication (50/95 - 52.63%), often co-existing with various complications and might be the cause of most of them. MOF happened in 10/50 patients with 3 deaths. (4) A major operation might be a promoting factor to MOF. The operation for AHNP should be simpler, if possible, to reduce stroke in the patients who are often poor risks. All the basic operations for the complicated and changeful AHNP could not be as effective as those for cholecystitis or appendicitis. Earlier timing of the operation seemed to decrease the incidence of MOF, though not the mortality. (5) Year 31 cases of old patients ($\geqslant$60) had 12 (38.71%) with MOF and 7/64 cases (10.94%) was so in the younger ($P<0.05$).

PO133

### PSEUDOCYSTS OF THE PANCREAS - PROBLEMS OF THE SURGICAL TREATMENT

N Iaramov, Arc Ivanov, A Mitov, P Gerzilov

"Bialo More" str. No 8 - Sofia; University Hospital "Queen Joanna" - Centre of Urgent Medical Aid, Bulgaria

The authors have studied 420 cases of acute biliary pancreatitis. They have discussed in detail the clinic, diagnosis and treatment of the pancreatic pseudocysts. The cystic formations of the pancreas are relatively seldom found in comparison with other surgical diseases of the pancreas.

According to certain authors (Iaeld, Scott etc) pancreatic cysts are found in 0.0I to 0.005% of all hospitalised patients. The increase in such cases recently can be explained on the one hand by the better results from treatment and the better chance of survival of patients with acute pancreatitis and on the other hand by the increase of latent abdominal trauma.

Pancreatic cysts are found most often in patients aged between 30 and 60. Both men and women are affected at almost the same degree. According to RB Cattel the inflammatory forms (16.2%) are most often found, followed by the retention and neoplastic forms. Furthermore, the pancreatic cysts are divided into real cysts and pseudocysts, according to whether or not they are within their own capsule and whether they have an epithelium inside cover.

They report their experience in the treatment of 10 patients. They consider Jurasch operation as a method of choice in pancreatic pseudocysts with retrogastric localisation, while in the case of pseudocysts of neoplastic origin the method is total extirpation.

PO134

### HAEMODYNAMIC CHANGES IN ACUTE PANCREATITIS

Th Pavlis[1], G Baltopoulos[2], K Daskalakis[1]

1. 3d Surgical Dep. Evangelismos Hospital, Athens, Greece
2. Agioi Anargiri Cancer Hospital ICU, Athens, Greece

In this study we examined the possibility of differentiation of the acute interstitial pancreatitis (AIP) from necrotizing pancreatitis (NP) by haemodynamic criteria. Applying the invasive haemodynamic monitoring, include use of Swan-Gauz catheter, we made a comparative evaluation of patients with NP (Group A, n=12) and patients with AIP (Group B, n=10). The diagnosis of AIP is based on clinical and laboratory data that of NP on histological examination of surgical specimen.
The haemodynamics of the two groups were:

| | Group A | Group B |
|---|---|---|
| BP | 110 ± 25 | 118 ± 16 |
| HR | 128 ± 12 | 76 ± 14* |
| Pw | 7.8 ± 0.5 | 7.6 ± 0.4 |
| SVR | 780 ± 98 | 1445 ± 128* |
| PVR | 80 ± 16 | 142 ± 12* |
| CI | 4.8 ± 0.4 | 3.2 ± 0.4* |
| Qs/Qt | 26 ± 5.2 | 14 ± 4.6* |

BP = Arterial blood pressure (mmHg), HR = Heart rate, Pw = Wedge pressure (mmHg), SVR, PVR = Systemic and pulmonary vascular resistance (dyn.s.cm-5), CI = Cardiac index (l/min.m$^2$), Qs/Qt = Pulmonary shunt (%), * = Statistical significant at the level of 0.05, Values are mean ± SD.

Comparing the above haemodynamics we conclude that the AIP can be differentiated from NP by haemodynamic data. These data can be one of the criteria of urgent laparotomy in patients with acute pancreatitis.

PO135

### IMMUNOCORRECTION IN THE COMPLEX OF CURATIVE MEASURES FOR ACUTE PANCREATITIS

B S Briskin, Z I Savchenko, N N Khachatrian

Department of Surgery, Institute Semashko, Hospital N50, Moscow, Russia

34 patients aged 18-75 years with acute necrotizing pancreatitis were studied in a prospective clinical and laboratory trial. The treatment which had been proposed for the management of patients with pancreonecrosis included a wide variety of medical and surgical measures i.e. biliary drainage, early operative drainage of the pancreas, thoracic duct drainage and drainage of the peritoneal cavity if peripancreatic infection was recognised. In 12 patients the gland was oedematous, usually with scattered areas of fat necrosis. In this group mortality was 16 per cent. In 14 patients there was gross retroperitoneal and peripancreatic haemorrhage, and in this group mortality was 50 per cent. In 8 patients the findings were classified as a pancreatic phlegmon, mortality was 32 per cent. Examination of lymphocyte function showed reduced cell percentages in peripheral blood and T cell percentage, a serum inhibitor of sheep red blood cell rosette formation by T cells.

Our results show significantly less T helper cell a day or three after operation. Proliferative reactions by T cell to PHA mitogen are decreased as compared to controls. Patients produce less antibody than controls. No statistical difference can be identified in IgA response between 2 groups of patients with pyogenic complications or without them. Neutrophil chemotaxis and bactericidal function are decreased as soon as pyogenic complications are developed.

We have examined the levels of IL 1 and receptors on the surface on the sensitized lymphocytes in patients with pancreonecrosis, interactions between helper T cells, B cells and cytokines. These findings correlate statistically with an increased risk of death or purulent complications. The inclusion of immunocorrection (tactivin, thymogen, thymalin, thymoptin) in the complex of therapeutic measures after operation for acute pancreatitis led to a fall of pancreonecrosis mortality from 33-50 to 29 per cent.

PO136

### ACUTE PANCREATITIS DIAGNOSTIC CRITERIA AND THERAPEUTIC OPTIONS

M Jekić, I Jekić, M Putniković

Surgical Service, Clinical Hospital Centre,
Zemun-Belgrade

We will present a clinical prospective study of one hundred cases of acute pancreatitis, admitted to our service during a period of 35 months, trying to evaluate, above all, the criteria of diagnosis and the therapeutic options according to the various clinical presentations.

The diagnosis on clinical and/or ultrasonographic bases was not very precise, having a high number of diagnostic laparotomies as a consequence. A better diagnostic accuracy was achieved with the paracentesis or peritoneal lavage. The evaluation by computed tomography was not possible because it was infrequently available at our emergency service.

Biliary surgery was the most common surgical treatment in acute pancreatitis and direct pancreatic surgery was performed only in cases of necrotizing pancreatitis or in the treatment of pancreatic complications.

Morbidity and mortality rates depended, above all, on the initial severity of the pancreatitis, but intensive medical care of the "severe pancreatitis" and early surgical treatment of the local complications, had a favourable influence on the final outcome.

The overall mortality rate was 4% in all cases as a result of pancreatic sepsis.

PO137 ACUTE EDEMATOUS PANCREATITIS:USE OF I.V. SOMATOSTATINE VS. S.C. LONGASTA TINE.CLINICAL RESULTS IN 24 CASES.

Sortini A.,Carrella G.,Zamboni P.,
Navarra G.,Santini M.,Donini I.

Clinica Chirurgica Generale-Università
degli studi-Ferrara-Italia.

The Aa. present their experience on 24 cases of edematous acute pancreatitis without hemorrhagic or necrotizing complications,at presentation.
The patients have been divised into 2 groups:group A (12 pt.,50%) was treated by i.v. continuous perfusion of somatostatine for 6 days using 3,5 mcg./Kg./h. per day;group B (12 pt.,50%) was treated by s.c. longastati ne for 6 days using 0,1 mgr. every 8 hours per day.
In group A complete recovery was obtained without any adjunctive therapeutic procedure in 11 cases (91,6%),3 of wich developed pancreatic pseudocyst during the follow-up (6 months), 1 case was operated on for hemorrha gic and necroziting pancreatitis and died in 14th post operatory day.Of the cases of pancreatic pseudocyst 2 have already been successfully operated on;the third is still waiting for operation.
in group B complete recovery was obtained in 10 cases (83,3%, n.s. in camparison with group A);the other two cases,in wich clinical evaluation showed an necrosing severity,theraphy was changed and i.v. continuous perfu sion of somastatine was used respectively in 4th and 5th day.these two cases had complete recovery,but devepo led pancreatic pseudocyst during the follow-up (one has already been successfully operated).
In conclusion, the treatment of acute edematous pancrea titis by means of i.v. somatostatine or s.c. longastatine had similar results.the incidence of late complications (psudocyst) wassimilar too. the preference accrded to longastatine in comparison with somastatine is based on an easier execution of the therapy and a higher cost containment.

PO138

### LONGITUDINAL PANCREATICOJEJUNOSTOMY ASSOCIATED TO THE FREY PROCEDURE

F Callejas Neto, J C Pareja, V F Pilla, L S Leonardi

Department of Gastroenterology Surgery, University of Campinas, Campinas-SP, Brazil

We retrospectively studied 18 patients with chronic pancreatitis associated with an enlarged fibrotic pancreatic head, who underwent longitudinal pancreaticojejunostomy. All patients were alcohol abusers presenting with abdominal pain and loss of weight. Jaundice was present in 10 patients, although alkaline phosphatase was elevated in 17 patients. Duodenal obstruction was present in 2 cases. During surgical procedure the pancreatic head was 7.0cm or more, with retention cysts in 14 cases.

All patients had common bile duct obstruction. The head of the pancreas was cored out and the cysts were drained as related by Frey. In 13 cases a carefull and meticulous dissection of the common bile duct in its intrapancreatic course was performed. However, in 2 patients with marked dilatation of choledocus and intense fibrosis we performed a separate biliary bypass. In the follow-up the patients were submited to a control E.R.C.P. which demonstrated a complete drainage of the pancreatic duct. The pain relief was considered excelent in all but two patients who persisted in alcohol consume.

PO139 OPEN TREATMENT FOR ACUTE NECROTIZING PANCREATITIS.

A.Chiappa°, A.Nespoli*, L.Dominioni°, O.Chiara*, F.Berizzi°, R.Dionigi°.
°Department of Surgery, University of Pavia-Varese, Ospedale Multizonale di Varese, Italy.
*Emergency Surgery Clinic, Univ. Milan, Italy

Mortality caused by generalized suppurative peritonitis and sepsis following acute necrotizing pancreatitis ranges from 30% to 80% depending upon the severity of disease. Reasons for this result primarily consist in the persistence of unrecognized and undrained intraabdominal foci leading to recurrent occult sepsis. Abdominal open treatment with the insertion of a Marlex mesh and zipper for daily exploration and lavage could reduce mortality and morbility.
PATIENTS AND METHODS: From January 1985 to June 1992 14 patients, 8 m. and 6 f. (age = 56±9 yr.) were treated in the Clinica Chirurgica of the University of Pavia, and in the Emergency Surgery Clinic of the University of Milan. All patients had a diagnosis of necrotizing pancreatitis and underwent open treatment for acute intraabdominal sepsis with insertion of a Marlex mesh and zipper for daily exploration and lavage. Preoperative ultrasonography and CT scan were performed. Postoperatively all patients underwent ICU treatment with Swan-Ganz catheter pulmonary artery pressure monitoring. At admission in ICU the average Goris score for multiorgan suffering was 5.1 for survived patients and 5.9 for non survivors.Basal caloric expenditure was measured with indirect calorimetry, using an analyzer of expiratory flow. When intestinal motility was present, a support enteral nutrition or mixed parenteral-enteral nutrition was performed. Serial cultures of blood, exudate from laparotomic wound and of other secretions were used to establish antibiotic prophylaxis or therapy when necessary.
Five patients died (31%); sepsis and multiorgan failure (MOF) were the main reasons for all deaths.
Postoperative complications were septic shock (4pts), splanchnic bleeding (3 pts), anastomosis leakage (1pt), MOF (1pt), pancreatic fistula (1pt), colic perforation (1 pt).
DISCUSSION AND CONCLUSION: Pancreatic sepsis following necrotizing pancreatitis is an unsolved problem related to frequent life-threatening complications and marked tendency toward recurrence, even in patients treated with traditional surgery. Poor results in this critical condition have suggested the development of more aggressive techniques.
The open treatment compared to the traditional closed techniques has the advantages that it permits a continuous, effective and complete drainage thus preventing abscess formation, sepsis recurrence, particularly in the most severe cases.

PO140 # COMPLICATIONS OF PANCREATIC NECROSIS

A Chaudhary, P Bhalkar, R C Aranya

Department of Gastrointestinal Surgery G.B. Pant Hospital, New Delhi-100 002, India

Ninety eight patients underwent surgery for pancreatic necrosis over a period of 7 years (1985 - 1992). Twenty-two (22%) of these had some complication because of the necrotic process. Nine patients haemorrhaged (intra-abdominal in 5, upper gastro-intestinal tract bleeding in 4); necrosis of colon was seen in 5 patients, necrosis of the medial wall of the duodenum in 3 and isolated bile duct necrosis was seen in 2 patients and in one patient the necrosis involved the spleen.

A total of 34 surgical procedures had to be done in these 22 patients. Five (22%) patients died, 3 because of haemorrhage and one each with duodenal and colonic necrosis. In contrast, in the 76 patients who had no complication, 4 (5%) patients died.

Pancreatic necrosis is a serious disease with a potential for causing varied complications each of which needs a planned surgical approach. Of all the complications of pancreatic necrosis haemorrhage seems to be the most lethal and difficult to treat.

**PO141** SURGICAL TREATMENT IN CHRONIC CALCIFIED NON-ALCOHOLIC PANCRETITIS

P. TENCHINI,T.BRUNI, G. CALEFFI, O. BRUNI
C. PULICA

1st Surgical Unit,Carlo Poma Hospital -
Mantova - Italy

The film presented the surgical treatment with pancreatico-jejunostomy in a 68 years old male patient suffering from chronic non-alcoholic pancreatitis with intractable pain,an alteration in his general condition and insulin-dependent diabetes for the last 8 years. The patient presents an obliterant arteriopathy affecting the lower limbs on an arteriosclerotic base.The CAT scan and pre-operative ERCP revealed a calcified chronic pancreatitis with dilatation of Wirsung's duct,occupied by stones situated,above all,in the head. The most interesting points of the technique qre to locate the Wirsung duct by puncture and to perform a direct Wirsung-graphy with exact morphological definition, extraction with Fogarty balloon catheter of the pancreatic calcifications found within the duct and finally, fashioning of a one-layer, wide side-to-side transmesocolic wirsung-jejunostomy on a Roux-en-Y loop, that guarantees adeguate pancreatic drainage, in the presence of a firm stenosis of the duct in its cephalic trajectory.
The patient was discharged on the 12th postoperative day, after 7 days on total parenteral nutrition.
In the long-term out-patient controls, the patient referred to complete relief of pain and weight gain.

PO142

### INFLUENCE OF DNA PLOIDY, PATHOLOGIC FACTORS, SERUM CA 19-9 ON PROGNOSIS OF RESECTED PANCREATIC CARCINOMA.

C. Sperti, C. Pasquali, # A. Piccoli,

Semeiotica Chirurgica, # Medicina Interna - University of Padua,

DNA content has been recently suggested to provide a significant prognostic information in pancreatic cancer Several histologic factors as well as serial Ca 19-9 assay have been studied and found to be related to prognosis after resective surgery. In this study we analyzed retrospectively, 26 specimen of consecutive resected pancreatic carcinomas to assess the rate of aneuploidy and the relationship between survival, DNA patterns and histopathologic features. All patients had serial CA 19-9 assay before and after surgery.

50 micrometer sections of primary tumors were prepared and analyzed by FACS cytometer. All patients had a ductal adenocarcinoma; 21 pancreaticoduodenectomy and 5 left pancreatectomy were performed. 15 patients had a radical surgery and 11 had microscopical residual of the tumor. 12 patients had a well differentiated tumor while 14 had a moderately or poorly differentiated tumor. Ten patients were in stage I, 4 in stage II and 12 in stage III according to UICC stage system. Twelve/25 had lymp-node metastases. In 8/25 (32 %) of cases the primary tumor was aneuploid. Excluding 3 pts with preoperative low CA 19-9 levels, 10 out 23 had fall of CA 19-9 levels to normal range after surgery. No relationship was found between size, stage, lymph-node status or tumor grading and DNA ploidy. Postoperative fall of CA 19 -9 is significantly related to survival (p = 0.004; median 19 mo. in low vs 8 mo. in high ); only 1 /10 patients with low postoperative CA 19-9 survived less than 12 months and only 2/13 with high CA 19-9 survived > 12 months. No relationship was found between ploidy and postoperative fall of CA 19-9. Lymph-node status (p = 0.02) and stage (I vs II-III; p= 0.04) were found correlate to survival Despite preliminary findings suggested that DNA ploidy may have prognostic value in pancreatic cancer, we are not able to confirm relationship between ploidy of the primary tumor and survival after surgical resection.

DNA ploidy is not related to metastatic potential of the tumor; CA 19-9, lymph node status and stage are the main factors we have found to influence prognosis.

PO143 PALLIATIVE SURGERY OF THE PANCREATIC CANCER

F. Seferiadis, A. Mystakidis, G. Svoronos,
G. Garinis, N. Lambrinaki, N. Kitromilis,
G. Velmachos
"Sotiria" General Hospital, Surgical Unit,
Athens, Greece

If the malignant tumor proves to irremovable, the majority of surgeons perform one of the many short-circuiting or decompressive operations.

The choise of these palliative procedures continues to be cholecysto-jejunostomy using the Roux-en-Y loop or the simple jejunal loop technique combined with side-to-side jejunojejunostomy or end-to-side hepaticojejunostomy is carried out to circumvent the possibility of subsequent duodenal obstruction.

Authors report a retrospective study of patients with primary pancreatic tumors who underwent palliative procedures in our department.

They were 32 male and 22 female who ranged in age from 48 to 82 years. Surgical treatment included laparotomy with biopsy alone in 8 patients, choledochojejunostomy in 31 and cholecystojejunostomy in 15 patients. The mortality rate was 9,2%. In 38 patients a side-to-side jejunojejunostomy was carried out. A number of surgeons consider that cholecystogastrostomy is the operation of choise for anatomical reasons. This procedure, however, has disadvantages.

The results of the palliative operations are disappointing, as the average mortality rate is about 10% and within 5-9 months of the operation nearly all such patients are dead. When survival exceeds 1 year the question always arises as to whether or not the primary lesion in the pancreas was in fact cancerous.

PO144

### SAFETY AND FUNCTION OF ISOLATED ROUX LOOP PANCREATICOJEJUNOSTOMY AFTER WHIPPLES RESECTION

A.N. Kingsnorth

University Department of Surgery,
Royal Liverpool University Hospital, Liverpool UK

A novel method of pancreatic anastomosis following proximal Whipple-type resection (classical PD or pylorus-preserving PPPD) has been evaluated over a five year period, 1987-92 in 52 patients.

Indicatations for resection included chronic pancreatitis (n = 9) and neoplasms (n = 43). Reconstruction involved a cephalad end to end duodeno-/gastro-jejunal anastomosis with a biliary anastomosis 6-8cm downstream. A separate isolated, defunctioned Roux loop was used to construct a duct-to-mucosa pancreaticojejunostomy.

Median postoperative stay was 18.0 days (11-32). Three deaths (operative mortality 5.8%) occurred due to sepsis (subhepatic abscess), profound hypoglycaemia and necrotising pancreatitis respectively. These deaths were not related to pancreatic fistula. There were no pancreatic leaks (defined as >50ml amylase-rich fluid for more than 7 days)

Twenty patients considered to have normal pancreatic remnants underwent a PABA excretion test at 3-18 months after operation. median PABA excretion index as 48% (24-100%). Only 4 of 43 (9.3%) patients resected for neoplasm required pancreatic supplements after operation.

Isolated defunctioned duct-to-mucosa pancreaticojejunostomy is a safe procedure offering good functional results after Whipple's PD or PPPD resection.

PO145

### EXTENDED EXCISION OF THE AMPULLA OF VATER. OUR FIRST SIX CASES EXPERIENCE

E Gadžijev, V Pegan

Department of Gastroenterologic surgery,
Medical Center, Ljubljana, Slovenia

The extended excision of the ampulla of Vater was first performed by author in 1989. It was published as a new operative technique in Hepato-Gastroenterology in 1992. The procedure comprises a pylorus-preserving resection of the descending segment of duodenum, excision of a large part of pancreatic head herewith concerning the anatomic pattern of common bile duct and pancreatic duct, which are running together up to the level of subpyloric region. Regional lymph nodes are removed and reconstruction with Roux-en-Y jejunal loop on the subpyloric part of the duodenum and both ducts sutured together, performed.

Our first six cases are presented, patients for the operation being strongly selected. Their basic data, pathology, operative variations, postoperative course, morbidity as well as short term survival time are described.
There were four patients with ampullary tumors, one with periampullary tumor and one with duodenal carcinoma. Four of them are alive and well, the first operated patient, who had had recidivant ampullary carcinoma died because of the progress of the disease two years after the extended excision, and one patient died 34 days after the operation from heart attack.

According to our first experience the advantages of the described procedure are: it is more radical operation than simple excision, it is less mutilant than Whipple operation, with few possibility for complications, and resection as well as reconstruction can be simply performed.
Disantvantages may be: questionable radicality for some cases, problems with reconstruction when pancreatic duct is not dilated, possibility of common poscibial difficulties after pylorus preserving resections.

We conclude that more operations should be done for better evaluation of the value of this operative procedure.

PO146

### EXPERIENCES OF PANCREATODUODENECTOMY FOR PANCREATIC AND PERIAMPULLARY CANCER

Dian-Chang Wang, Tou Sun

Tianjin Cancer Hospital, 300060 China

260 patients with cancer of pancreas and periampullary region were treated surgically in our hospital during the period March 1963 through December 1987. 60 cases underwent pancreatoduodenectomy. Operative rate was 23.07% (60/260). Operative mortality rate was 15% 99/60) but it' 6.06% in 1980'.

Morbidity rate of pancreatic fistula was 33.3% in 1960 and it was only 3.03% in 1980.

Stump of pancreatic remnant was invaginated into open end of jejunum in 47 cases and end to side pancreatojejunostomy was in 12 and simple ligation was only in one.

Extracorporeal pancreatic juice drainage by long silicon tube through jejunostomy was done in 32 cases with only one pancreatic fistula.

29 cases died during follow-up and 28 cases died within 2 years.

5 year survival rate was 36.6% in whole group.
5.8% for cancer of pancreas
42% for cancer of ampulla of Vater

PO147 TRUE CYSTS OF THE PANCREAS. CLINICAL ANALYSIS OF 20 CASES.

L Harsányi, T Winternitz, N Csoszánszky
L Flautner, T Tihanyi

1st Surgical Department, Semmelweis Medical School, Budapest, Hungary

M+M: True cysts of the pancreas are relatively uncommon (under 10% respectively).10 cystadenomas (CA) and 10 cystadenocarcinomas (CAC) were treated in our department from 1972 to 1992. Average age of the patients was 51 years. ♂/♀ = 5/15.

Results: The main initial symptoms of the cystic tumors were abdominal pain or dyscomfort, weight loss and palpable abdominal mass without any previous inflammatory episode. Laboratory investigations revealed nonspecific alterations only. CA 19-9 and CEA levels did not show any specific increasing. Size, cystic character and site of the lesions were clearly shown by sonography and computed tomography in all cases. Preoperative/intraoperative fine-needle-biopsy helped to distiguish and frozen-section confirmed the correct diagnosis in all cases. The exact diagnosis was only possible in 7 patients before the operation. The most frequent site was the tail of the pancreas (9/20). All CA and 5 of CAC patients underwent radical resections, they are still alive.

Conclusion: According to our data the radical exstirpation of the cystic tumors is the method of choice in CA/CAC patients.

PO148

### SOLID PAPILLARY CYSTIC EPITHELIAL NEOPLASM OF PANCREAS IMPROVED DETECTION DUE TO BETTER AWARENESS

P Jagannath, L J de souza, M Bhansali, S C Krishnamurthy, K Mohandas, V Shantiswaroop

Tata Memorial Hospital, Bombay 400 012, India

Solid Papillary cystic epithelial neoplasm of Pancreas (SPCENP) is now recognised as a distinct clinico-pathologic entity occurring in young females.

In the last year 3 young females were diagnosed pre-operatively as SPCENP due to better awareness of the condition. A retrospective analysis of pancreatic resection for cystic tumours of the pancreas over a five year period (1987-1991) was undertaken to identify SPCENP. Specific information was obtained regarding pre-operative diagnosis, imaging and the surgical approach. Only 4 more cases could be identified. This indicates referral pattern, as a large lesion in pancreas is generally associated with poor prognosis. In this series of seven female patients the age range was 16-40 years with two elderly patients aged 35 and 40, an unusual feature. Pre-operative diagnosis in earlier periods was adenocarcinoma of pancreas/cystic tumour of pancreas. Imaging errors included diagnosis of liver abscess, pseudocyst. One patient underwent cystogastrostomy elsewhere due to mistaken diagnosis of pancreatic pseudocyst. Pre-operative histology was obtained in recent three cases by fine needle aspiration cytology. 6/7 lesions were resected completely by distal pancreatectomy. One patient underwent Whipple's resection for SPCENP of head of pancreas. There was no detectable recurrence in this series with follow up ranging from 3-60 months.

It is important to identify SPCENP as a distinct entity as resectability is good, with better prognosis.

PO149

### PROGNOSIS OF THE ADENOCARCINOMA OF THE HEAD OF THE PANCREAS TREATED BY PANCREATICO-DUODENAL RESECTION (PDR) (37 CASES)

J M Andreu, I Sielezneff, B Sastre, M J Payan

Service de chirurgie (Pr Sarles)
Hopital Ste Marguerite, 270 bd de Ste Marguerite, 13009 Marseille, France

The purpose of this retrospective study was to assess the survival rate after PDR for adenocarcinoma of the head of the pancreas and to find out some survival predictive factors.
37 patients (pts) (23 men, 14 women, mean age : 62 years) were operated on between 1980 and 1990. Nine pts benefited of pyloric preservation, 5 of veinous resections. Digestive reconstruction always used a pancreato-jejunal anastomosis.The mean rate of blood units (BU) transfused was 4,7 (0 to 16). The pancreatic sections were always desease-free. Lymph node involvement was observed in 21 cases (57%) ; proximal lymph nodes (N1) invasion was present in 8 pts while distal spread (N2) was observed in 13 pts. The influence of age, sex, follow-up, pyloric preservation, lymph node involvement, blood transfusion on the survival rate was studied.
The survival rates were calculated by the Kaplan Meier technique and were compared by the Log-rank test. Multiple regression analysis was used to adjust for differences in prognostic variables.
The mortality was 5,3% and the specific morbidity (pancretic fistula) was 5,7%. The 5 years actuarial survival rate (after exclusion of the surgical deaths) reached 9%, the median survival was 15 months. The duration of the survival was not modified by age, sex, follow-up and pyloric preservation. The 5 years actuarial survival rate was either not influenced by lymph nodes invasion ($p=0,053$) ; the length of the survival was longer for pts with N1 (19% at 5 years)than those with N2 (no survival after 2 years). ($p<0,05$); pts No (17%) and pts N1 had the same actuarial survival rate ; blood transfusion worsened the survival rate ($r = -1,29$). These results therefore suggest that distal lymph node involvement and/or blood transfusion reduce the actuarial survival rate of pts who benefited of PDR for adenocarcinoma of the head of the pancreas.

PO150

### IS THERE A PLACE FOR GASTROENTEROSTOMY IN PATIENTS WITH ADVANCED CANCER OF THE HEAD OF THE PANCREAS?

G P van der Schelling, R P van den Bosch, J H G Klinkenbijl, P G H Mulder, J Jeekel

Department of General Surgery, Erasmus University Hospital, Rotterdam, the Netherlands

There remains doubt about the necessity for gastro-enterostomy in patients with advanced cancer of the pancreatic head, either performed prophylactically or when the passage of food becomes impossible. The records of 142 patients admitted for advanced pancreatic cancer to the Erasmus University Hospital over a period of 11 years were reviewed. We concentrated especially on the pre- and post-operative intake of food in cases involving gastro-enterostomy, and the morbidity and mortality associated with abdominal surgery in these patients. Of 129 patients without symptoms of gastric outlet obstruction at the time of diagnosis, 31 underwent prophylactic gastro-enterostomy. This could not prevent gastric outlet obstruction in 4 patients. Of the remaining 98 patients, 15 developed gastric outlet obstruction. Cox proportional hazards analysis showed no significant difference in the interval to the occurrence of a symptomatic obstruction between these two groups, taking into account other co-variables. Post-operative complications and mortality regarding a gastro-enterostomy were high, ranging from 9-41% and 11-33% respectively.

Our results do not significantly indicate that prophylactic gastro-enterostomy may prevent future gastric outlet obstruction; and therefore, as it also increases morbidity, it should not be performed. A gastro-enterostomy for symptomatic reasons should be considered carefully as the success rate is low and is accompanied by a considerable incidence of morbidity and mortality.

### PO151 CT STAGING OF PANCREATIC TUMORS

MS van Leeuwen[1], MAM Feldberg[1], H Obertop[2], AH Hennipman[2], CD Kooyman[3]. Departments of Radiology[1], Surgery[2] and Pathology[3], University Hospital Utrecht, the Netherlands.

MATERIAL and METHODS

In 40 patients who were referred for a possible curative resection of a pancreatic tumor pre-operative CT scanning was performed in order to assess the presence and extent of tumor. A detailed CT staging system was used wherein the tumor extent with regard to the splanchnic vessels was precisely described. Also, TNM staging was performed and the feasibility of a resection was predicted.

These CT findings were correlated with the findings at laparotomy and, if resection was performed, with the resected specimen, using micro- and macro-pathology. In order to correlate the CT images with the pathology findings the entire resected specimen was examined in the transverse plane.

RESULTS

The extrapancreatic direct extent of tumor was accurately predicted in 89 % of patients. However, the sensitivity for liver metastases and peritoneal implants was only 61 and 29 %. The assessment of nodal involvement was poor, with equally low sensitivity and specificity.

CONCLUSION

The direct extent of pancreatic tumor can be well visualized with CT, performed as an out patient procedure. However, the low sensitivity for distant metastases is a strong argument for staging laparoscopy, in patients who seem to have resectable disease on CT.

PO152 MALIGNANT TUMOUR OF THE BODY OF THE PANCREAS: CORPO CAUDAL RESECTION

T. BRUNI, C. PULICA, A. VIOTTO, O. BRUNI
P. TENCHINI

1st Surgical Unit, Carlo Poma Hospital -
Mantova - Italy

Distal pancreatic resection with splenectomy is often indicated in the neoplasms of the body and tail of pancreas. Fortner in 1977 indicated total extended pancreatectomy as the most appropriate intervention in adenocarcinoma of pancreas; we believe in accordance with japanese authors that a real advantage is obtained by a careful linfoadenectomy.
In our video is shown a body and tail pancreatic resection extended with a splenectomy and total gastrectomy for an adenocarcinoma of the pancreatic body and tail with an infiltration of the posterior wall gastric body with previous bleeding.
The patient was a male 59 years old with a weight of 72 Kg.in a very good state of health.
Very important by a technical point of view were the loco-regional linfectomy and the substitution of retropancreatic portal vein a gore-tex prothesis.
In our opinion such an extended resection is justified in a young patient to obtain a radical intervention and a careful linfoadenectomy.

PO153 DUODENOPANCREATECTOMY FOR HEAD ADENOCARCINOMA

M.BUSANI, C. PULICA, O. BRUNI, P. TENCHINI

L.FRANCIA

1st Surgical Unit,Carlo Poma Hospital -
Mantova - Italy

In this video a duodenopancreatectomy is shown. It was an adenocarci noma of the pancreatic head in a 62 years old male.
First of all the diagnostic approach pre and intraoperative with remark to ultrasonography and intraoperative fine needle aspiration.
A careful estimation of operability is performed, especially as regards connections with vessels.
It is to enphasize the loco-regional lynphoadenectomy and the reconstruction which we performe with a double intestinal loop:end-to-side gastro-jejunal anastomosis; end-to-side choledocal-jejunal anastomosis and end-to-end pancreatis jejunal anastomosis.

PO154

### PYLORUS PRESERVING PANCREATODUODENECTOMY WITH EXTENDED LYMPHATIC CLEARANCE IN THE TREATMENT OF PANCREATIC CANCER.

P.C. GIULIANOTTI, A. CAROBBI, U. BOGGI, G. SARTONI, T. BALESTRACCI, A. COSTA, F. MOSCA

Istituto di Chirurgia Generale e Sperimentale
Pisa University Medical School, Italy

In recent experiences, mainly from Japanese Authors, the extension of the lymphatic clearance beyond regional nodes, either alone or associated with intraoperative radiation therapy, has been reported to improve survival especially in small carcinomas.

Pylorus preserving pancreatoduodenectomy, initially proposed in the treatment of benign diseases, has increasingly been applied also to the treatment of pancreatic cancer. An extended lymphatic clearance including, in addition to regional, iuxta-regional (second level: porta hepatis, common hepatic, retroportal, celiac) and third level nodes (from the diafragmatic cruras above to the origin of the inferior mesenteric artery below, from the right renal hilum to the preaortic nodes on the mid-line, and from there to the left renal hilum), as well as an en bloc resection of portal vein, can be easily carried out also during this procedure. The unresected perigastric nodes would be unlikely to influence survival because their involvement occurs only at an advanced stage when prognosis is very poor regardless of the treatment.

Since February 1991 all patients with histologically proven diagnosis of ductal adenocarcinoma have been included in a perspective randomized trial designed to evaluate survival rates, recurrence patterns and post-operative mortality and morbidity rates in two groups of patients undergoing pancreatoduodenectomy with either standard or extended lymphatic clearance. Out of ten patients included in the study five have received an extended lymphadenectomy, with an additional en bloc resection of the portal vein in two cases.

In this video the surgical techinique of the extended lymphatic clearance during total pancreatectomy with pylorus preservation is illustrated in detail. Operative morbidity, mortality and preliminary survival rates are also reported.

PO155

### EVALUATION OF GASTRIC EMPTYING OF SOLID MEAL IN THE EARLY FOLLOW UP OF PYLORUS PRESERVING PANCREATODUODENECTOMY

N Mazzuca*, PC Giulianotti, T Balestracci, D Mazzotta,
A Carobbi, A Costa, I Zappelli, V Morini*, F Mosca

Institute of General and Experimental Surgery University of Pisa
*Department of Nuclear Medicine, Livorno Italy

Delayed gastric emptying in the early postoperative course of pancreatic resection with gastric preservation has often been reported. Hormones, neurological impairment as well as electromechanical factors have been related to this phenomenon. After the full recovery of gastrointestinal function, which takes place in 10-15 days, gastric emptying seems to be clinically normal. The aim of this study was to evaluate the gastrointestinal motility after ingestion of a solid meal (technetium-99m labeled eggs) in the early follow up of pylorus preserving pancreatoduodenectomy.
24 patients (18 M 6 F, mean age 58.54 +/- 9.9 yrs) were included in the study. The indications for surgery were in 12 cases the presence of a pancreatic cancer, in 6 a periampullary neoplasm, in 5 a chronic pancreatitis and in 1 pt. a neuroendocrin non secreting neoplasm. The mean time of nasogastric suction was 12.1 +/- 3.9 days. The morbidity rate was 37.5% and the mean post operative time was 17.6 +/- 6.9 days.
The scintigraphic technique was performed with a computerized gamma camera with the head centered upon the patient's epigastrium. The test was conducted in a mean time after surgery of 45.4 +/- 22.6 days, all the patients were clinically asymptomatic with a free diet.
The data collection consisted of 12 frames of one minute duration each every 15 minutes after ingestion of the solid technetium meal (boiled egg). Images were elaborated by means of ROI (Region Of Interest) delineated over the epigastrium.
The gastric emptying was then calculated as decrease rate of the epigastric activity. A 50% reduction of the initial activity (T 1/2) was chosen as standard value (between 70 and 120 minutes in normal subjects).
18 patients showed an accelerated gastric emptying (T1/2 = 30.83 +/- 12.72 min.) and 6 a normal one (T1/2 95 +/- 11.28 min.). In no instance a delayed gastric emptying was found (T1/2 > 120 min.). No patient had symptoms referable to an accelerated gastric emptying.

PO156

### NON ENDOCRINE PANCREATIC AND PERIAMPULLARY MALIGNANCIES IN YOUNG ADULTS

P.C. GIULIANOTTI, U. BOGGI, A. GUADAGNI, G. CARAVAGLIOS, D. GIARDINO F. MOSCA

Istituto di Chirurgia Generale e Sperimentale
Pisa University Medical School, Italy

Although rare, pancreatic neoplasms are not exceptional even in patients under age 40 years, and a slightly better survival rate has been reported for this age group.

Medical records of 373 consecutive patients (mean age 64.06 $^{+}/_{-}$ 11.15 yrs.; range 17-90 yrs.) with non-endocrine and periampullary malignancies admitted to the Department of General and Experimental Surgery of Pisa University in the period between January 1982 and November 1992 were reviewed. Eight patients (3 m; 5 f) less than 40 years old (2.14%) were identified. The mean age was 30.25 $^{+}/_{-}$ 7.7 yrs. (range 17-38 yrs.).

No relevant data emerged from either family or patient's past medical history. No patient had Peutz-Jeghers syndrome, which seems to be associated with a greater incidence of pancreatic cancer in young adults.

Presentation symptoms included primarly jaundice (50%), dyspeptic syndrome (50%), weight loss (more than 5 kg in the three months before the diagnosis) (37.5%) and abdominal pain (37.5%). In one case the only complaint was a palpable epigastric mass and in another the tumor was an incidental finding during cholecystectomy.

Tumors (4 pancreatic cancers, 2 papillary-cystic neoplasms, 1 CBD cancer, 1 papillary cancer) were mainly located in the head of the pancreas or in the periampullary region (75%). Resections (5 pylorus preserving pancreatoduodenectomies and 2 distal pancreatectomies with en bloc splenectomy) were performed in all but one patient, with a pancreatic cacner with bilateral liver metastasis, who underwent a biliary drainage (resectability rate: 87.5%). No hospital deaths occured and the post-operative course was uneventful in all but 2 cases (25%) in whom two fluid collections (one peripancreatic and the other subfrenic) were successfully treated by percutaneous drainage.

Three out of four patients with pancreatic carcinoma died of cancer : mean survival time in this group was 7.86 $^{+}/_{-}$ 6.1 months. All the other patients are still alive: papillary-cystic neoplasms 3 and 18 months, pancreatic cancer 51, CBD cancer 72, papillary cancer 114 after surgery respectively.

Although these numbers are too small to allow any significant conclusion or comparison, the dismal outcome of young adults with pancreatic adenocarcinoma seems to be similar to that of the older patients.

Two papillary-cystic tumors, an unusual clinico-pathologic entity, were reported. These neoplasms, tipically found in females in the second and third decades of life, are considered malignant because of their potential for invasion of the fibrovascular capsule as well as their ability for metastatic spread. However, complete surgical resection is usually curative, with no long term recurrence in most cases. Even metastatic spread of this particular tumor is often amenable to surgical treatment with valuable long term results.

PO157

### CLINICAL VALIDATION OF TUMOR-ASSOCIATED ANTIGEN ASSAYS IN THE PREOPERATIVE DIAGNOSIS AND FOLLOW-UP OF PATIENTS WITH PANCREATIC CANCER

M Ferdeghini, PC Giulianotti, A Costa, J Romagnoli, C Annicchiarico, C Prontera, R Bianchi, F Mosca, G Mariani.

Nuclear Medicine Center and Institute of General and Experimental Surgery of the University of Pisa Medical School; Pisa (Italy).

Although at immunohistochemistry the newly identified tumor-associated antigen CAR-3, a mucin-like molecule non-cross-reactive with CEA or the CA19.9 antigen, is expressed in a large fraction of pancreatic cancers (>85%), preliminary studies on circulating CAR-3 in such patients yielded a sensitivity of only 44-51%. The aim of this study was to comparatively assess, in a larger series of patients, the clinical usefulness of a serum assay for CAR-3 as a tumor marker for the diagnosis and follow-up of pancreatic cancer.

The serum levels of CAR-3 were measured by a solid-phase RIA kit (kindly provided by SORIN Biomedica, Saluggia, Italy) in a total of 231 patients with various diseases of the GI tract: 61 with pancreatic cancer (18 stage I, 3 stage II, 10 stage III, 30 stage IV, by TNM classification) and 160 with non-neoplastic disease of the GI tract, including 22 with pancreatitis (acute in 6, chronic in 16) and 6 with benign pancreatic tumor. Serum samples from the follow-up of 21 pancreatic cancer patients were also assayed. The results of the CAR-3 assay were compared with those of the CEA, CA19.9 and CA195 antigens.

At the cut-off level of 6.2 U/mL (based on non-parametric estimate of a 90% specificity), the sensitivity of CAR-3 was 62.3% (*versus* 75.4% for CA19.9, 23% for CEA and 73.8% for CA195, respectively). Increasing the specificity to 95% (cut-off 13.7 U/mL) reduced senstivity to 47.5% for CAR-3 (*versus* 70.5% for CA19.9, 14.8% for CEA and 67.2% for CA195, respectively). No significant differences were observed for the markers between patients in stages I-II and those in stages II-IV, with the following positivity results (at the 90% specificity level) for stages I-II: CAR-3 8/21, CA19.9 14/21, CEA 2/21 and CA195 14/21. In stages III-IV patients: CAR-3 26/40, CA19.9 32/40, CEA 12/40 and CA195 31/40. No significant differences were also found between patients with poor prognosis (42 patients with survival <1 year) and the 14 patients surviving more than 1 year. The preliminary results obtained in the follow-up phase indicate the similar reliability of CA19.9 and CA195 in predicting tumor recurrences, with satisfactory results for CAR-3, and worst of all for CEA. Finally, the results obtained in some patients with cholestasis confirm earlier reports of high CAR-3 serum levels in this clinical condition.

The results obtained in this study appear to confirm that the CAR-3 antigen is shed in circulating blood in a much lower proportion of the cases that that observed for antigen expression at immunohistochemistry. The better diagnostic and follow-up properties are exhibited in patients with pancreatic cancer by the CA19.9 and CA195 antigens.

PO158 SCREENING AND PHARMACOKINETICS OF THE MoAb CAR-3 FOR IMMUNOSCINTIGRAPHY IN PATIENTS WITH PANCREATIC CANCER

G Mariani, PC Giulianotti, D Bacciardi, N Molea, T Balestracci, C Bonino, M Ferdeghini, U Boggi, A Costa, R Bianchi, F Mosca, L Ferrante.

CNR Institute of Clinical Physiology, and University of Pisa Medical School; Pisa. SORIN Biomedica; Saluggia. University of Ancona Medical School; Ancona (Italy).

The tumor-associated antigen CAR-3, a mucin-like molecule non-cross-reactive with CEA or the CA19.9 antigen, is expressed in a large fraction of pancreatic cancers, as shown by immunohistochemistry. On the basis of favourable localizing properties shown in the experimental animal model of tumor xenograft, this study was aimed at assessing the potential usefulness of the murine monoclonal antibody (MoAb) AR-3, an $IgG_1$ specifically raised against the CAR-3 antigen, as an immunoscintigraphy agent in patients with pancreatic cancer. To this purpose, the MoAb was radiolabeled with $^{131}I$ for human tissue biodistribution studies.

$^{131}I$-AR-3 was injected i.v. as a single bolus into 5 patients with suspected pancreatic cancer. A computerized gamma-camera (GE Starcam 400) was utilized to record dynamic scintigraphic maps over the chest and abdomen from 0-30 minutes post injection. Static whole body maps were then recorded at daily intervals from 0.5-144 hours. Blood samples were also taken from 0.1-144 hours, and the fractions of circulating undegraded $^{131}I$-AR-3 and free $^{131}I^-$ were measured by Sephadex G25 chromatography. Specific ROIs for the heart, liver and spleen helped to define the kinetics of activity in these organs both in the dynamic sequence (0-30 minutes) and in the later phase (0.5-144 hours).

At surgery, pancreatic cancer was confirmed in 3 of the patients. Despite the favourable immunostaining properties of the AR-3 MoAb *in vitro* and the fact that in at least one of these patients the radioimmunoassay for circulating CAR-3 resulted to be positive, in none of the patients with pancreatic cancer did the labeled MoAb localize in their tumors in sufficient amounts as to result in positive immunoscintigraphy. Whereas, some radioactivity accumulation was detected in the overall pancreatic region, thus resulting in a weak scintigraphic outline of the pancreas itself. Nonspecific uptake of $^{131}I$-AR-3 in the liver and spleen was very low, while pharmacokinetic modeling identified a saturation-like, nonlinear zero-order process for activity accrual due to binding in these organs. Doubling times were 1.83 ± 0.17 hours and 2.13 ± 0.31 hours, respectively, after subtracting radioactive contributions from blood circulating in these organs. This data compares with late removal from these sites having half-lives of 65-75 hours. Integration of these kinetic parameters into a complex multicompartmental model helped to define the exchange and removal rates of $^{131}I$-AR-3 with greater accuracy than conventional pharmacokinetic modeling based solely on linear, first-order kinetics.

The results obtained in this study confirm that the preliminary screening in humans of potential immunoscintigraphy agents is an esential requirement, even with MoAb tracers showing favourable *in vivo* targeting properties in the animal model.

PO159 NESIDIOBLASTOSIS IN THE ADULT SURGICAL MANAGEMENT

J.Palla Garcia, J.Pedroso, C.Cardoso

Hospital de Santa Marta, Rua de St.Marta

1100 - Lisboa - Portugal

The nesidioblastosis in the adult it's one excceedingly rare occurence and when it appears it is part of $MEA_1$ syndrome.

We present one case of nesidioblastosis in a young women without associated endocrine pathology, meanwhile we discuss specialy the diagnostic and therapeutic problems of this condition.

The histopathologic and imunohistochemic features of the ressected pancreas are analysed in detail.

The treatment was by sub-total pancreatectomy (70 - 80%) showing the histopathologic and imunohistochemic examination of the specimen: "Nesidiodisplasia with pancreatic cells and with insulin secreting in excess - nesidioblastosis".

After the pos-operative recover, during the following year, two hypoglicemic crises recur, and then we choose to practice adjuvant therapy with somatostatin in perfusion (five days) two cycles with total remission of symptoms, without hypoglicemic crisis since then - one year.

The patiént is in follow-up for determination of the need of reoperation and performance of near total pancreatectmy or the continuation of the sucess with the somatostatin treatment.

PO160

### ENDOCRINE TUMOURS OF THE HEAD OF THE PANCREAS AND PERIAMPULLARY AREA

C Pasquali, C Sperti, *M Guido, A Alfano D'Andrea, C Filipponi, S Pedrazzoli

Semeiotica Chirurgica - University of Padua, *Pathology, Cittadella, Italy

Endocrine tumours of the periampullary area and the head of the pancreas (PET) may require an extended surgical procedure for cure. Since 1968 we observed 37 patients with proven PET: 19 cases of insulinomas, 9 Zollinger-Ellison Syndrome and 9 "non functioning" tumours. 18/19 insulinomas were typical single adenomas of the head of the pancreas ranging in size from 1.0 to 4.0 cm. 14 were treated by local excision 2 with pancreato- duodenectomy (PD) and two with subtotal head resection; all patients were cured by surgery. One patient had a multiple endocrine neoplasia (MEN 1) and 4 adenomas were excised from the head of the pancreas. The patient is still normoglycaemic 8 years later. Five/9 patients with ZES had multiple tumours (2 had MEN 1) and 3 patients had microadenomas (size < 0.5 cm). Five cases were treated with a PD, 4 had an excision of duodenal adenomas. In two cases ZES was not cured by surgery and one recurred 14 years after surgery.

Five patients had normal gastrinaemia after 5 - 16 years follow-up. Six/8 patients with non-functioning tumours underwent resective surgery. Two PD, 1 partial head and body resection for double adenoma, 2 local excisions and 1 partial duodenal resection. One of them died 6 months later for tumour progression, and 4 are disease free after 1 to 11 years follow-up. One/3 patients with liver metastases was still living 5 years after diagnosis.

While preservation of the disease free pancreas is the rule in insulinomas and extended procedures are usually due to technical problems, in our series 4/19 (21%) had a pancreatic head resection at least subtotal.

Since long term survival in non beta PET is a frequent event these tumours, even if advanced, require an aggressive surgical approach, as a curative resection or a long term palliation can be frequently achieved.

PO161 *LEFT PANCREATECTOMY WITH PRESERVATION OF THE SPLEEN*

B. PRADERE, E. BLOOM, CH. JULIO, J.L GOUZI.

C.H.U. PURPAN - Place du Dr BAYLAC

31059 - TOULOUSE Cedex - FRANCE

The septic risk after splenectomy, although a matter of controversy, must become a surgeon's concern in left pancreas resections for benign or traumatic diseases.

Twelve patients with benign tumors of the tail of the pancreas and one with a traumatic rupture of the neck of the pancreas underwent a left pancreatectomy with preservation of the spleen. The splenic pedicle was always resected along with the left pancreas.

The pancreas is exposed by separating the omentum from the transverse colon taking care not to harm the short gastric vessels. The tail of the pancreas is mobilized and the splenic vessels are ligated and divided proximal to the splenic hilum. Left pancreatectomy is completed by division of the neck of the pancreas and of the medial segment of the splenic pedicle.

Residual blood flow to the spleen originates from short gastric, gastroepiploic and left gastric arteries.

This technique was described for pancreas donation from a living relative and is anatomically possible in most cases. It must become the standard in patients undergoing left pancreatectomy for a benign disease.

PO162 DIAGNOSIS AND TREATMENT OF HYPERINSULINISM IN PEDIATRIC PATIENTS.

I.Fontana, V.Arcuri. G.V.Tommasi, V.Casolino, U.Valente

Dept. of Surgery, Transplant Unit, University of Genoa- San Martino H.Genoa, Italy.

Hypoglycemic disorders, due to an absolute or relative increase of insulin secretion, may represent a complex diagnostic and therapeutic problem in pediatric patients by presenting possible cerebral lesion due to the lack of glucose and ketons.
Our statistic includes 7 patients aging from 3 months to 14 years, with stable or recurrent non ketogenetic hypoglycemia with convulsive crises beginning sometimes in the first hours of life.
The operative sequence was:

1- Assess the presence of hyperinsulinemic state (insulin plasma level > 150nu/ml, or lack of insulin secretion'suppression during hypoglycemiaor inadeguate secretion) by means of insulin/glucose index and also by looking at the metabolic effects of hyperinsulinism ( inibition of lipolisis, increase of cellular incorporation of aminoacids) or using stimulation testwith leucine or glucagone. We don't use suppression test.
2- Medical theraphy- We use parenteral administration of glucose to mantein blood glycemia over 40 mg/dl. The use of diazoxide doesn't give very good results. We don't use chlorpromazine or phenytoin or streptozocinand somatostatinfor the inibitor effect on other hormones.
3- Differential diagnosis between nesidioblastosis and B-cell adenoma: the study of proinsulin as biochemical markerwas an inconclusive diagnosis. Only in two cases (28,5%) we localized pre-operatively a B-cell adenoma( one by CAT and the other by arteriography).
4- Surgical theraphy: it was the ultimate theraphyin all cases. We found 3 β-cell adenoma ( one in the tail, one in the head and one in the mesentery). In the other patients we performed a subtotal pancreatectomy for nesidioblastosis. The surgical procedure was done without removing the spleen.In all patients the follow-up shows a good metabolic control.

P0163 **EMERGENCY RETRANSPLANTATION YIELDS HIGHER MORTALITY RATE THAN ELECTIVE RETRANSPLANTATION IN PAEDIATRIC LIVER TRANSPLANTATION.**

T Yandza, F Gauthier, J Valayer.
Surgical unit, Department of Pediatrics, Bicetre Hospital, Kremlin Bicetre, France.

Survival rates have been reported to be lower after orthotopic liver retransplantation (re-OLT) than primary liver transplantation (OLT). The aim of this retrospective study was to determine whether mortality rate was similar in cases of re-OLT compared to elective re-OLT. Between January 1988 and October 1991, 100 liver transplantations were performed in 85 children (mean age: 44.4 months). Fourteen of these patients (16%) underwent re-OLT. Five patients were retransplanted in elective conditions (group 1): four patients had a second graft and one had two new grafts. Indications for retransplantation were four hepatic artery thrombosis (HAT) and two vanishing bile duct syndromes. In this group, all patients but one who had an elective re-OLT for HAT had had an attempt for immediate surgical revision. In group 2, nine children had a second graft in emergency for primary liver non function (n=5), HAT (n=3), portal vein thrombosis (n=1). In this group, no patient retransplanted for HAT had had an attempt for revascularization. The overall survival of patients retransplanted was 71%. In group 1, there was no death, while in group 2, 5 deaths (55%) occured. Our results suggest that, in paediatric liver transplantation, retransplantation might be performed in elective conditions when possible, as emergency retransplantation yields higher mortality rate. As HAT was the main cause for retransplantation, this emphasizes the interest of immediate surgical revision which might delay retransplantation until recipient state allows retransplantation in better conditions.

P0164

### A PROSPECTIVE EVALUATION OF LONG-TERM INJECTION SCLEROTHERAPY FOR BLEEDING OESOPHAGEAL VARICES

JEJ Krige, PA Goldberg, GN Stapleton, PC Bornman, J Terblanche.
Surgical Gastroenterology, Department of Surgery and MRC Liver Research Centre, University of Cape Town and Groote Schuur Hospital, Cape Town, South Africa.

The efficacy of long-term injection sclerotherapy (IST) in eradicating oesophageal varices after endoscopically proven variceal bleeding was assessed prospectively in 204 patients during a 66 month period between 1984 and 1989. Data were analyzed in July 1992 to allow a minimum 30 month follow-up. The 204 patients (127 men, 77 women; mean age 50.3, range 16-82 years) underwent 1022 emergency and elective injection treatments with 5% ethanolamine oleate using a combined intra and paravariceal technique during 1860 endoscopy sessions during the study period. The majority (167;82%) had cirrhosis, mainly due to alcohol (131;64%). The modified Pugh-Child's risk grades were A:26, B:94, C:84. Seventy-five (37%) of the 204 patients had a total of 130 bleeding episodes after the first hospital admission before eradication of varices (0.03 bleeding episodes per patient month of follow-up). Rebleeding was markedly reduced after eradication of varices. Long-term assessment in the 100 (87%) of 114 patients who survived >3 months, varices were eradicated after a mean of 5 injections and remained eradicated in 47 [mean followup: 41.5 months; range:5-90 months]. Varices recurred in 53 patients and rebled in 18 of whom only 8 rebled from oesophageal varices. Cumulative survival by life table analysis was 55%, 42%, and 32% at 1,3 and 5 years. Prognosis was influenced by Childs grade and aetiology of portal hypertension. One hundred and thirteen patients (55.4%) died during follow-up. Liver failure was the most common cause of death. Of the 236 complications which occurred in 139 (68.1%) patients, mucosal slough (137 patients) was the most common. A localised injection-site leak occurred in 9 patients and oesophageal stenosis developed in 23 patients of whom 14 required dilatation (mean:4; range: 1-7 dilatations). Free oesophageal perforation occurred in 5 patients, 4 of whom died.
Repeated fibreoptic.IST eradicates oesophageal varices in the majority of patients with a reduction in rebleeding. Complications related to IST were mostly of a minor nature but became cumulative with time.

PO165 PORTAL-SYSTEMIC ENCEPHALOPATHY: ALTERED BRAIN INDOLE- OR CATECHOLAMINE SYNTHESIS?

B Alexander, M Aslam, The Late A Nobin, IS Benjamin.

Department of Surgery, King's College School of Medicine, London, England.

The pathogenesis of portal-systemic encephalopathy (PSE) is complex but may be divided into 2 broad sub-components of portal-systemic diversion and hepatic dysfunction: differentiation between these may rationalise effective clinical therapy. Both these sub-components are expressed in the portacaval shunted rat (PCS) but only portal-systemic diversion is observed in the portacaval transposed (PCT) rat with little or no pathological cerebral lesions (Benjamin et al 1984, Doyle et al 1978). Abnormal brain amine metabolism may be a contributory factor in the pathogenesis of PSE (Bengtsson et al 1988). This study was conducted to determine the contribution that altered brain indoleamine and brain catecholamine metabolism may make towards experimentally-produced PSE in PCS rats, achieved by comparison to the changes measured in the brains of PCT rats.

80 male Sprague-Dawley rats were subject to PCS, PCT or sham operation and studied at 1, 3, 9 and 15 weeks following the operations. Injection of the monoamine oxidase inhibitor NSD 10/15 (m-hydroxy benzylhydrazine) (100mg/kg i.p.) permitted measurements of the indole amines 5-hydroxytryptophan (5-HTP), 5-hydroxytryptamine and 5-hydroxyindole acetic acid (5-HIAA) and also of the catecholamines noradrenaline, adrenaline and dopamine to be made in the midbrain and cerebrum of the three groups of rats. Only sustained changes were measured after 15 weeks in brainstem levels of 5-HIAA (0.68 ± 0.01 PCS vs 0.37 ± 0.04 PCT $\mu$g/g brain) and 5-HTP (0.24 ± 0.05 PCS vs 0.22 ± 0.02 PCT $\mu$g/g brain). No consistent changes were measured in any of the catechol amines measured in the brainstem or cerebrum and it is suggested that previous reports regarding the efficacy of L-DOPA in providing transient amelioration from PSE is via a mechanism other than elevations in brain dopamine levels alone. These data suggest that an alteration in brain indole metabolism may contribute towards the changes seen during PSE.

Bengtsson F., Nobin A., Falk B., Gage FH., Jeppsson B. (1988) World J Surg 12: 246-254.

Benjamin IS., Ryan CJ., Englebrecht GHC., et al. (1984) Hepatology 4: 704-708.

PO166 SURGICAL TREATMENT OF SEVERE CHRONIC HEPATIC ENCEPHALOPATHY

MJ Boudet, JS Dermesropian, G Zeitoun, JM Hay, Y Flamant

Service de Chirurgie, Hôpital Louis Mourier
178, rue des Renouillers 92701 Colombes Cedex, France

Hepatic encephalopathy (HE) is one of the main complications of surgical portocaval shunts. Symptoms are most of time moderate but can be severe in 5 percent of cases, leading to major neurophychiatric features. No medical treatment has been satisfactory for patients suffering of severe chronic hepatic encephalopathy (SCHE). Low hepatic inflow has been incriminated in the physiopathology of SCHE. Surgical procedures to restore a good hepatic inflow have been advocated : 1/ portal vein arterialization (PVA) ; 2/ suppression of the portocaval shunt. Results of these two surgical procedures are reported herein.

**Patients**

From january 1976 to september 1992, seven patients were operated on for severe chronic hepatic encephalopathy (SCHE). All of them were males with a mean age of 56 ± 3 yrs (range : 49 to 58 yrs). All patients had cirrhosis (alcoholic : 4, cryptogenic : 3), and had been shunted for recurrent bleeding varices. Shunts were as follows : 3 end-to-side portocaval shunts,1 side-to-side splenorenal shunt, 3 Warren shunts. Onset of SCHE appeared with a mean of 16 months (range : 1 to 78 months) after shunt procedure.

**Preoperative evaluation**

All patients had routinely : 1/ neurological examination ; 2/ Electroencephalography (EEG) ; 3/ liver needle biopsy ; 4/ hepatic arteriography ; 5/ hepatic vein angiography with measurement of wedged and free hepatic veinous pressures ; 6/ measurement of hepatic vein outflow.

**Surgical treatment**

Warren shunts (3 cases) were ligated. Recurrence of esophageal varices were prevented by two total gastrectomies and one upper partial gastrectomy with an interposed small bowel graft. The four remaining patients had a PVA.

**Results**

Shunt ligation : 1 patient died on postoperative day 68 with no clinical nor EEG improvement Two patients are alive (6 and 52 months follow-up) with clinical and EEG improvement. Portal vein arterialization : all patients died : 3 postoperatively (days 3, 18 and 19), and one 30 months after surgery, with no clinical nor EEG improvement.

**Conclusions**

Surgical portocaval shunt may lead to SCHE. PVA does not improve SCHE and has a high mortality rate. Shunt ligation seems to lead to clinical and electroencephalographic improvement for patients suffering of SCHE. Recurrence of variceal bleeding must be prevented. Liver transplantation should be evaluated.

PO167

### REDUCED UPPER URINARY TRACT DRAINAGE AFTER INJECTION SCLEROTHERAPY: INDIRECT EVIDENCE OF VAGAL INJURY?

**H. Kynaston, S. Grime, J. Yates, N. Davies. B. Taylor, K. Parsons & S.A. Jenkins**

**Department of Surgery, Royal Liverpool University Hospital, Liverpool, U.K.**

It is well known that injection sclerotherapy (IS) of oesophageal varices with sclerosing agents such as ethanolamine oleate (EO) may give rise to local and systemic side effects. Recent evidence has suggested a vagal component in the reflex drainage of the upper urinary tract. Since IS is thought to adversely influence vagal function the aim of this study was to assess its effect on upper urinary tract drainage.

Twenty-five patients undergoing gastroscopy with a view to perform IS were studied. Dynamic scintigraphy using Mag 3, a renal tubular agent, was performed 24 hours pre and 24 hours post gastroscopy.

Twenty patients underwent IS, 10 of whom received > 10 ml of EO. The time activity curves in those patients who received EO were prolonged being more so on the right. The percentage of the injected dose remaining in the kidney at 16 minutes was significantly increased in both kidneys (mean increase on right = 2.3%; 95% CI 0.45,3.9%, mean increase on left = 1.6%; 95% CI 0.18,3%, $p < 0.05$). The subgroup of patients receiving > 10ml of EO had an even greater retention if isotope at 16 minutes (mean increase on right = 3.8%; 95% CI 0.28,7.3%, mean increase on left = 2.0%; 95% CI 0.35,3.6%, $p < 0.05$). No such changes were observed in the 5 patients who did not receive sclerotherapy.

Upper urinary tract drainage following IS is significantly prolonged and may be secondary to vagal injury at the time of IS. Injury may be due to local inflammation and oedema and thus be transient or may be due to extravasation of sclerosant which could be permanent and possibly cumulative.

PO168

### RESULTS OF PARTIAL PORTAL DIVERSION IN CIRRHOTIC PATIENTS

D Grange, C Vons, A Karaa, D Mariette, C Smadja, D Franco.

Services de Chirurgie, Hôpital Louise Michel, Evry, et Hôpital Antoine Béclère, Clamart, France.

Between december 1989 and December 1992, 22 patients with cirrhosis and previous variceal bleeding were electively treated by a partial portal diversion. There were 13 men and 9 women. Mean age was 55 years (range : 34 to 68 years). Ten patients were in Child-Pugh's group A and twelve patients in group B. Calibrated portacaval shunts were performed using a 10 mm PTFE graft. Portal flow was assessed on the 6th postoperative month by angiography and doppler pulsed ultrasonography and thereafter every six months by doppler pulsed ultrasonography. There were no postoperative death. Three patients had an obstructed shunt on postoperative control angiography (13.6 %). Two of them subsequently had a mesocaval shunt. One patient developed late thrombosis of the prosthetic graft on the 6th postoperative month by a neoplastic thrombus complicating a hepatocellular carcinoma. Among the 18 other patients portal diversion was partial in 11 (61 %) and total in 7 (39 %). Four patients developed encephalopathy (22 %). Two among 11 patients (18 %) with a partial shunt had acute self-limited bouts of encephalopathy. None en them had chronic encephalopathy. Two among 7 patients with a total shunt (28 %) had moderate chronic encephalopathy. One-year survival was 89.5 %. These results suggest that 10 mm H-graft portacaval shunt results in partial portal diversion in more than half the patients. They confirm that in patients with partial diversion of portal blood the rate of chronic encephalopathy is low.

PO169

### SMALL PORTAL VEINS AND PAEDIATRIC ORTHOTOPIC LIVER TRANSPLANTATION.

T. Yandza, F. Gauthier, J. Valayer.
Surgical unit, Department of Pediatrics, Hopital Bicetre, Kremlin Bicetre, France.

Considered previously to be a contraindication for orthotopic liver transplantation (OLT), small portal veins are no longer a reason to reject paediatric patients who are candidates for OLT. The aim of this study was to determine the frequency of small portal veins and their effect on the outcome of paediatric OLT. Of 85 chidren (mean age: 44.4 months; range: 3 to 156 months) receiving 100 liver transplants, two groups were studied according to portal vein diameter: in group 1 (n=12), portal vein diameter was <4mm, and in group 2 (n=73), portal vein diameter was >4mm. The main indication for OLT was biliary atresia in both groups with a higher percentage in group 1 (9/12=75%) compared to group 2 (39/73=53%) (NS). There was no significant difference between the two groups in terms of age and weight. In group 1, the mean portal vein diameter was 3 mm versus 6 mm in group 2 ($p<0.001$). Three children (25%) presented with portal vein thrombosis in group 1, leading to variceal bleeding episodes in two cases, and to death in one. There was no portal vein thrombosis in group 2 ($p<0.002$). In conclusion, small portal veins are encountered in 14% of paediatric OLT and are associated with a higher risk of portal vein thrombosis. Progressive decrease in portal vein diameter observed in children awaiting OLT should be an indication of rapid transplantation.

PO170

### HAS LIVER TRANSPLANTATION MODIFIED THE PLACE OF PORTAL-SYSTEMIC SHUNTING FOR THE MANAGEMENT OF RECURRENT VARICEAL BLEEDING IN ALCOHOLIC LIVER DISEASE?

**B Meunier, J F Bretagne, S Landen, M Messner**
**B Launois**

**Department of Digestive Surgery and transplantation Unit, Pontchaillou Hospital, Rennes, France**

Parallel to the development of sclerotherapy, the indications for portal-systemic shunting have sharply decreased.
Nevertheless, they retain a place in the treatment of recurrent variceal bleeding.

Between January 1972 and December 1988, 125 patients with alcoholic liver cirrhosis and at least two episodes of bleeding varices underwent a shunting procedure. The patients'mean age was 51 $\pm$ 10 years (range 18 - 71 years).
64 patients were Child A, 47 were Child B, 13 were Child C and 1 was undetermined. The types of shunts performed were : 112 end-to-side portal-caval anatomoses (89.6 %), 1 side-to-side portal caval anastomosis, 3 meso-caval anastomoses, 6 proximal spleno-renal anastomoses, and 3 distal spleno-renal anastomoses. The end to side portal-caval anastomosis was the standard procedure, with the proximal spleno-renal shunt being preferred in the presence of hypersplenism, and the meso-caval shunt being performed prior to liver transplantation. Operative mortality was 8 % (n = 10) and was correlated with the Child classification. Postoperative complications included ascites, variceal bleeding and encephalopathy in 5, 4 and 11 patients respectively. Survival (Kaplan-Meier curve) at 1,5 and 10 years was 77 %, 40 %, and 18 % (operative mortality included). Survival was not related to the Child status but improved significantly in patients abstaining from alcohol. Late complications (13 %) included chronic ascites (n = 3) and encephalopathy (n = 13 ).

Despite success in the early follow-up period, the results of portal-systemic shunting are poor in the longer term, with high rates of late comlications. These are to be compared to the stable results obtained with liver transplantation. A randomized prospective trial could be considered.

PO171 **EMERGENCY SHUNT SURGERY AS RESCUE TREATMENT OF CONSERVATIVE MEASURES FAILURE IN PATIENTS WITH VARICEAL BLEEDING.**

R.Santambrogio, E.Opocher, M.Intra, B.Ongari, and G.P. Spina.

Istituto di Scienze Biomediche S.Paolo - Università di Milano

Patients with acute bleeding from esophageal varices must be treated by non-operative measures and emergency sclerotherapy seems to guarantee a better early control than other therapies. When they fail to arrest bleeding or prevent early recurrence, emergency porto-systemic shunts (EPSS) should be undertaken without further procrastination. Forty-four patients (A:5; B:25; C:14 according to Pugh-Child's classification) out of 104 bleeders required EPSS,that permanently controlled the variceal bleeding in all but 1 patients (with patent shunt as demonstrated by angiography). Esophageal varices disappared in 21 patients (53%) and were reduced in 19 (47%). Four patients (9%) died within one month of the operation before the endoscopic examination: the causes of death were hepatic failure in 3 and bleeding ulcerations of the gastric fundus in the other patients. Twenty-one patients out of the 40 discharged alive subsequently died during a mean follow-up period of $33 \pm 32$ months.Eight died of hepatic failure, 3 of hepatomas, 2 of other neoplasia, 5 of peptic ulcer and 3 of other extrahepatic causes. Analysis of actuarial survival curves showed that the 5-year survival rates were fewer for Child's C patients than for A and B combined (18% vs 46%; $p<0.05$). During the follow-up period none of the patients had variceal bleeding. Chronic encephalopathy developed in 7 (18%) which was mild in 3 and severe in 4 instances. Thus, when conservative treatment fails to arrest variceal bleeding, EPSS should be performed to guarantee definitive control of hemorrhage and prolong the survival period.

PO172

### PAEDIATRIC LIVER TRANSPLANTATION: A SINGLE CENTRE EXPERIENCE.

T Yandza, F Gauthier, J Valayer.
Surgical unit, Department of Pediatrics, Bicetre Hospital, Kremlin Bicetre, France.

We report our experience with the first 100 liver transplantations (OLT) in children at Bicetre Hospital. From 1988 to 1991, 85 children received 100 liver grafts (mean age: 44.4 months). The indications for OLT were cholestatic diseases in 63 children, metabolic diseases in 14, and miscellaneous in 8 cases. Fifty-four per cent of the grafts were reduced-size grafts. The immunosuppression associated cyclosporine, steroids, and azathioprine. Actuarial survival rate at 4 years is 86%. Retransplantation was performed in 14 children (16%). Forty-four patients (49%) were reoperated. Biliary complications (17%), hepatic artery thrombosis (HAT) (14%), and haemoperitoneum (14%) were the most frequent surgical complications. Immediate surgical revision for HAT allowed to avoid retransplantation in 50% of cases. ABO incompatible liver grafts seemed to be more tolerated in children free of alloantibodies at the time of transplantation. Survival rates of ABO identical, compatible, and incompatible liver grafts did not differ (61%, 50%, and 57% respectively). Our results suggest that an agressive policy seems to be a necessary condition in order to achieve a satisfactory survival rate and quality of life. Children lacking ABO alloantibodies at the time of transplantation might better tolerate ABO incompatible liver grafts.

PO173 **THE IMMUNOMODULATORY EFFECTS OF LASER IRRADIATION IN HUMAN MIXED LYMPHOCYTE CULTURES (PANCREATIC ISLETS TRANSPLANT PROGRAM)**

A.Severtsev*, Y.Mullen**, P.-Y.Benhamou**, V.Lebedjuk*
* Pancreatic Research Lab,Surgical Dpt., Hosp.#51, Moscow, Russia; ** Diabetes Research Centre, UCLA, Los Angeles,USA

This study was made in the frameworks of the new Pancreatic islets transplant program in treatment of type I diabetes in Russia. It was started at the UCLA (CA, USA) and continued in Moscow (Russia). Treatment of type I diabetes by transplantation of pancreatic islets has met with only limited success thus far, due in part to the inability to prevent graft rejection by conventional immunosuppressants. Suggestion for solving this problem have included pre-treatment of recipients or of islets (UV irradiation, gamma irradiation, prolonged or reduced temperature culture and anti MHC class II antibody treatment). Very good idea is to use some wavelengths of laser irradiation spectrum for these purposes.

Long-term goal of the study: to investigate the effectiveness of laser light (different wavelengths) as a means to change the immunogenicity of human adult islets, and to modulate the immune response of the recipient.

Specific aim of the study: to evaluate the effectiveness of He-Ne laser irradiation (632.8 nm) of human lymphocytes to change the immunogenicity of these in vitro (mixed lymphocyte culture - MLC).

Methods: Heparinized blood (25 cc) was collected from 5 volunteers. Lymphocytes were separated on a Ficoll-hypaque gradient (LSM, Organon), washed and prepared for laser irradiation and MLC.

Laser irradiation of lymphocytes: Laser light source - HeNe/20mW, red, 632.8 nm, d spot=1 cm, Uniphase 1096,model 1135P; lense:MH-2P,model VPH2, Newport Corp. Lymphocytes were irradiated for 1, 10 and 60 minutes.

MLC was usual procedures. The 3H-thymidine and $\gamma$-irradiation (3000 rads) were used for MLC (reaction). After 6 hrs cultures were harvested, and counted with a liquid scintillation counter. Each experiment was made in triplicate.

Results:Results were expressed as a stimulation index (SI), defined as the ration of experimental cpm to control cpm. The results showed that HeNe-laser irradiation stimulated (in 3 cases) the human immunogenicity up to 3 times over control or didn't change it in other 2 cases.

